# ﻿A taxonomic revision of the Malagasy endemic subgenus Mayria of the genus *Camponotus* (Hymenoptera, Formicidae) based on qualitative morphology and quantitative morphometric analyses

**DOI:** 10.3897/zookeys.1081.71872

**Published:** 2022-01-17

**Authors:** Nicole Rasoamanana, Brian L. Fisher

**Affiliations:** 1 Madagascar Biodiversity Center, BP 6257, Parc Botanique et Zoologique de Tsimbazaza, Antananarivo, Madagascar Madagascar Biodiversity Center Antananarivo Madagascar; 2 Entomology, California Academy of Sciences, 55 Music Concourse Drive, San Francisco, CA 94118, USA California Academy of Sciences San Francisco United States of America

**Keywords:** Madagascar, morphometry, NC-Clustering, subgenus *Mayria*, taxonomy

## Abstract

The subgenus Mayria of the genus *Camponotus* (Hymenoptera: Formicidae) is revised. The subgenus is endemic to Madagascar where it occupies a broad range of habitats, from deciduous and dry forest to rainforest. A taxonomic review is provided of this subgenus, integrating multiples lines of evidence including qualitative morphology and quantitative morphometry. Species hypotheses are formed by Nest Centroid clustering. In total, 36 species are treated, of which eleven are newly described: *Camponotusandrianjaka***sp. nov.**, *Camponotusantsaraingy***sp. nov.**, *Camponotuschrislaini***sp. nov.**, *Camponotusclaveri***sp. nov.**, *Camponotusivadia***sp. nov.**, *Camponotusjjacquia***sp. nov.**, *Camponotusmaintilany***sp. nov.**, *Camponotusnorvigi***sp. nov.**, *Camponotusihazofotsy***sp. nov.**, *Camponotustsimelahy***sp. nov.**, *Camponotuszoro***sp. nov.** Five species are redescribed. *Camponotusthemistocles* Forel **stat. nov.**, is raised to species. In addition, the subgenus is redefined to include 39 species. Twenty-two previously described species are transferred to this subgenus and thirteen species previously placed in the subgenus are transferred out of the subgenus. Nine morphologically consistent species groups are delineated to facilitate species identification within the subgenus. This revision includes a classification, a key to species groups, and an updated key to species based on the minor worker caste.

## ﻿Introduction

The genus *Camponotus* Mayr, 1861 is one of the most speciose and widespread genera within the Formicidae, comprising more than 90 described species and subspecies in the Malagasy region. This genus has received renewed attention over the last five years. In 2016, the *Camponotusedmondi* species group was revised by Rakotonirina et al. (2016). Later, *Camponotusgrandidieri* and *niveosetosus* species groups, the subgenera *Myrmopytia* and *Mayria* have been revised (Rakotonirina et al. 2017; Rasoamanana et al. 2017; Rakotonirina and Fisher 2018). In total, 41 species were included in these studies, which combined qualitative morphological analysis, multivariate morphometry, and ecological data. Here we continue to explore the taxonomy of Malagasy *Camponotus* and revisit the subgenus Mayria. We redefine the subgenus and describe additional species using morphological features combined with morphometry.

In the 2018 revision by Rakotonirina et al., 14 species were recognized in the subgenus. However, our further studies of Malagasy *Camponotus* species have revealed additional morphological traits leading to new groupings. This revision increases the size of subgenus Mayria with the transfer of 15 species from the *edmondi* species group (Rakotonirina et al. 2016) and six species from *niveosetosus* species group (Rakotonirina et al. 2017), and the description of eleven new species and the redescription of six previously described species. Together, these changes increase the total number of species in this subgenus to 39. We divide this subgenus into nine species groups: *alamaina* group, *antsaraingy* group, *darwinii* group, *edmondi* group, *efitra* group, *ellioti* group, *madagascarensis* group, *repens* group, and *robustus* group.

## ﻿Materials and methods

This study is based on specimens collected from the ant inventory of Madagascar project as part of a joint collaboration between CAS and Parc Botanique et Zoologique Tsimbazaza under the aegis of the Ministry of Higher Education in Madagascar. After morphological examination, all workers were separated into morphospecies, and measurements of the 19 characters were recorded for specimens in good condition. The data were subjected to a multivariate analysis, which utilized the Nest Centroid (NC)–Clustering method to determine whether differences existed among workers within the same species. The degree of inclination of pilosity is particularly important for diagnosing groups or species. In this context, we use the terms erect, suberect, subdecumbent, decumbent, and appressed following Wilson (1955).

### ﻿Abbreviations of depositories

Specimens were deposited in the following collections:

**CAS** California Academy of Sciences, San Francisco, CA, USA

**MHNG** Muséum d’Histoire Naturelle, Geneva, Switzerland

**MNHN** Muséum national d’Histoire naturelle, Paris, France

**MSNG** Museo Civico di Storia Naturale di Genova “Giacomo Doria”, Genova, Italy

**NHMB** Naturhistorisches Museum, Basel, Switzerland

**PBZT** Parc Botanique et Zoologique de Tsimbazaza, Antananarivo, Madagascar

**PSWC** P. S. Ward Collection, University of California at Davis, CA, USA

**ZMHB** Museum für Naturkunde, Humboldt-Universität, Berlin, Germany

### ﻿Morphological study

All morphological observations were made with a Leica MZ9.5 stereomicroscope. We morphometrically investigated 307 individual workers belonging to 215 collecting events for the eleven newly described species and six redescribed species. *Camponotus* samples were collected across Madagascar by BLF and the Madagascar Biodiversity Center team (Table 1). The material is deposited in the California Academy of Sciences (CAS), San Francisco, USA. Data for all pinned specimens examined in this study are available on the web portal AntWeb (https://www.antweb.org) and can be accessed using their unique identifying specimen code (e.g., CASENT0188388). Images are linked to their specimens via the unique specimen code affixed to each pin. Type material and samples used in the morphometric analysis are given for each species after "Additional material examined" in the following order: Province, locality name, latitude, longitude, elevation (m), habitat, collector and abbreviation of depository. The most common collectors are abbreviated as follows:

**Table 1.** Ratios of morphometric data for minor and major workers.

**Species**	**Caste**	**CS**	**PoOC/CL**	**PrAC/CL**	**ClyL/CL**	**ClyL/GPD**	**CW/CL**	**CWb/CL**
*andrianjaka*	minor (N=9)	0.84±0.07	0.08±0.01	0.20±0.01	0.10±0.01	0.24±0.01	0.33±0.02	0.31±0.01
		[0.81, 0.88]	[0.08, 0.09]	[0.19, 0.21]	[0.09, 0.10]	[0.23, 0.25]	[0.32, 0.34]	[0.30, 0.32]
	major (N=3)	1.15±0.05	0.26±0.03	0.55±0.00	0.34±0.02	0.89±0.05	0.81±0.01	0.84±0.01
		[1.12, 1.20]	[0.23, 0.27]	[0.54, 0.55]	[0.32, 0.35]	[0.83, 0.92]	[0.79, 0.82]	[0.84, 0.85]
*antsaraingy*	minor (N=6)	2.14±0.38	0.08±0.02	0.22±0.02	0.11±0.02	0.25±0.05	0.32±0.01	0.34±0.03
		[2.00, 2.33]	[0.07, 0.09]	[0.21, 0.23]	[0.10, 0.12]	[0.22, 0.28]	[0.31, 0.32]	[0.32, 0.35]
	major (N=6)	3.32±0.21	0.26±0.00	0.54±0.01	0.27±0.05	0.74±0.14	0.78±0.01	0.96±0.01
		[2.94, 3.55]	[0.26, 0.27]	[0.52, 0.56]	[0.16, 0.30]	[0.46, 0.80]	[0.77, 0.81]	[0.95, 0.99]
*chrislaini*	minor (N=5)	0.95±0.17	0.12±0.03	0.18±0.01	0.11±0.02	0.26±0.03	0.36±0.03	0.34±0.03
		[0.87, 1.02]	[0.11, 0.13]	[0.18, 0.19]	[0.10, 0.12]	[0.25, 0.29]	[0.36, 0.38]	[0.32, 0.35]
	major (N=5)	1.67±0.06	0.29±0.02	0.51±0.02	0.34±0.00	0.92±0.03	0.90±0.02	0.92±0.03
		[1.57, 1.74]	[0.27, 0.31]	[0.49, 0.53]	[0.34, 0.35]	[0.86, 0.94]	[0.88, 0.93]	[0.90, 0.96]
*claveri*	minor (N=8)	0.89±0.08	0.10±0.02	0.18±0.01	0.10±0.03	0.23±0.07	0.35±0.02	0.33±0.02
		[0.84, 0.93]	[0.08, 0.10]	[0.18, 0.20]	[0.08, 0.11]	[0.20, 0.26]	[0.33, 0.35]	[0.32, 0.34]
	major (N=2)	1.18±0.09	0.26±0.00	0.54±0.00	0.33±0.01	0.88±0.05	0.84±0.01	0.86±0.01
		[1.12, 1.24]	[0.25, 0.26]	[0.54, 0.54]	[0.33, 0.34]	[0.85, 0.91]	[0.83, 0.84]	[0.85, 0.87]
*darwinii*	minor (N=37)	1.45±0.28	0.08±0.02	0.22±0.02	0.12±0.02	0.25±0.04	0.35±0.02	0.37±0.03
		[1.22, 1.63]	[0.06, 0.09]	[0.20, 0.23]	[0.10, 0.13]	[0.23, 0.28]	[0.33, 0.37]	[0.35, 0.41]
	major (N=14)	2.14±0.26	0.22±0.01	0.56±0.02	0.29±0.03	0.72±0.09	0.88±0.02	1.04±0.06
		[1.70, 2.47]	[0.19, 0.24]	[0.54, 0.59]	[0.20, 0.31]	[0.48, 0.81]	[0.85, 0.93]	[0.94, 1.20]
*ellioti*	minor (N=42)	1.85±0.55	0.08±0.01	0.22±0.01	0.12±0.01	0.28±0.03	0.33±0.02	0.36±0.03
		[1.45, 2.42]	[0.07, 0.09]	[0.21, 0.23]	[0.11, 0.13]	[0.25, 0.30]	[0.31, 0.34]	[0.33, 0.39]
	major (N=7)	4.13±0.16	0.28±0.01	0.56±0.01	0.31±0.01	0.87±0.05	0.80±0.01	1.04±0.02
		[3.90, 4.33]	[0.26, 0.29]	[0.55, 0.57]	[0.28, 0.32]	[0.79, 0.92]	[0.78, 0.82]	[1.01, 1.07]
*maintilany*	minor (N=14)	0.84±0.05	0.09±0.01	0.19±0.02	0.10±0.01	0.24±0.01	0.35±0.02	0.33±0.02
		[0.81, 0.86]	[0.08, 0.10]	[0.18, 0.20]	[0.10, 0.10]	[0.22, 0.25]	[0.34, 0.37]	[0.32, 0.34]
	major (N=3)	1.13±0.05	0.26±0.01	0.51±0.01	0.34±0.00	0.83±0.00	0.85±0.01	0.90±0.02
		[1.09, 1.18]	[0.25, 0.27]	[0.50, 0.52]	[0.33, 0.34]	[0.83, 0.83]	[0.83, 0.86]	[0.88, 0.91]
**Species**	**Caste**	**FR/CS**	**TCD/CS**	**SL/CS**	**MW/ML**	**PEW/CS**	**MPD/ML**	**NOH/CS**
*andrianjaka*	minor (N=9)	0.13±0.01	0.10±0.02	0.36±0.04	0.20±0.01	0.12±0.01	0.26±0.03	0.07±0.01
		[0.12, 0.13]	[0.09, 0.11]	[0.35, 0.38]	[0.19, 0.20]	[0.10, 0.12]	[0.24, 0.28]	[0.07, 0.08]
	major (N=3)	0.36±0.01	0.26±0.02	0.70±0.02	0.53±0.02	0.34±0.02	0.64±0.01	0.23±0.01
		[0.35, 0.37]	[0.25, 0.28]	[0.69, 0.72]	[0.50, 0.55]	[0.32, 0.35]	[0.63, 0.65]	[0.22, 0.25]
*antsaraingy*	minor (N=6)	0.15±0.01	0.10±0.01	0.44±0.04	0.27±0.02	0.17±0.03	0.22±0.01	0.13±0.03
		[0.14, 0.15]	[0.09, 0.10]	[0.42, 0.45]	[0.26, 0.28]	[0.16, 0.19]	[0.22, 0.23]	[0.11, 0.15]
	major (N=6)	0.35±0.01	0.24±0.01	0.81±0.04	0.66±0.03	0.31±0.02	0.55±0.03	0.21±0.02
		[0.35, 0.36]	[0.23, 0.24]	[0.76, 0.88]	[0.61, 0.71]	[0.27, 0.34]	[0.52, 0.60]	[0.19, 0.25]
*chrislaini*	minor (N=5)	0.17±0.01	0.10±0.01	0.46±0.03	0.22±0.02	0.15±0.02	0.26±0.06[	0.09±0.04
		[0.17, 0.18]	[0.10, 0.10]	[0.45, 0.48]	[0.21, 0.23]	[0.14, 0.15]	[0.24, 0.29]	[0.07, 0.11]
	major (N=5)	0.46±0.01	0.27±0.01	0.77±0.04	0.55±0.02	0.35±0.01	0.63±0.03	0.21±0.02
		[0.44, 0.47]	[0.26, 0.28]	[0.74, 0.83]	[0.54, 0.58]	[0.34, 0.36]	[0.59, 0.67]	[0.20, 0.23]
*claveri*	minor (N=8)	0.15±0.02	0.11±0.02	0.39±0.03	0.18±0.02	0.13±0.01	0.26±0.03	0.10±0.01
		[0.14, 0.16]	[0.10, 0.12]	[0.37, 0.40]	[0.18, 0.19]	[0.13, 0.14]	[0.24, 0.27]	[0.10, 0.11]
	major (N=2)	0.41±0.02	0.28±0.01	0.75±0.04	0.49±0.01	0.33±0.01	0.62±0.03	0.28±0.00
		[0.40, 0.42]	[0.27, 0.28]	[0.73, 0.78]	[0.49, 0.50]	[0.32, 0.33]	[0.60, 0.64]	[0.28, 0.28]
*darwinii*	minor (N=37)	0.14±0.01	0.11±0.01	0.40±0.04	0.21±0.02	0.13±0.03	0.24±0.03	0.11±0.02
		[0.13, 0.15]	[0.10, 0.12]	[0.37, 0.43]	[0.19, 0.23]	[0.11, 0.16]	[0.20, 0.26]	[0.09, 0.13]
	major (N=14)	0.35±0.01	0.27±0.01	0.83±0.08	0.56±0.02	0.30±0.03	0.59±0.03	0.25±0.03
		[0.33, 0.37]	[0.25, 0.28]	[0.75, 0.99]	[0.53, 0.58]	[0.23, 0.36]	[0.53, 0.67]	[0.21, 0.29]
*ellioti*	minor (N=42)	0.11±0.01	0.09±0.01	0.41±0.07	0.23±0.03	0.12±0.02	0.24±0.04	0.10±0.03
		[0.11, 0.12]	[0.08, 0.10]	[0.34, 0.46]	[0.21, 0.25]	[0.11, 0.14]	[0.20, 0.28]	[0.06, 0.12]
	major (N=7)	0.29±0.01	0.23±0.01	0.61±0.02	0.59±0.02	0.26±0.01	0.63±0.02	0.17±0.02
		[0.27, 0.30]	[0.22, 0.23]	[0.58, 0.65]	[0.56, 0.61]	[0.24, 0.28]	[0.61, 0.66]	[0.14, 0.19]
*maintilany*	minor (N=14)	0.15±0.02	0.10±0.01	0.40±0.03	0.19±0.02	0.13±0.03	0.25±0.02	0.10±0.01
		[0.13, 0.16]	[0.10, 0.11]	[0.39, 0.43]	[0.18, 0.21]	[0.11, 0.15]	[0.23, 0.26]	[0.09, 0.10]
	major (N=3)	0.36±0.01	0.25±0.01	0.77±0.03	0.51±0.01	0.30±0.03	0.66±0.03	0.25±0.01
		[0.35, 0.36]	[0.25, 0.27]	[0.74, 0.79]	[0.50, 0.52]	[0.28, 0.33]	[0.63, 0.68]	[0.24, 0.25]
**Species**	**Caste**	**HTL/CS**	**OMD/CS**	**EL/CS**	**MPH/ML**			
*andrianjaka*	minor (N=9)	0.39±0.02	0.17±0.03	0.11±0.01	0.14±0.03	–	–	–
		[0.38, 0.40]	[0.16, 0.19]	[0.10, 0.11]	[0.12, 0.15]	–	–	–
	major (N=3)	0.84±0.04	0.46±0.01	0.21±0.01	0.39±0.01	–	–	–
		[0.81, 0.88]	[0.44, 0.46]	[0.20, 0.21]	[0.38, 0.40]	–	–	–
*antsaraingy*	minor (N=6)	0.52±0.07	0.17±0.01	0.10±0.01	0.21±0.02	–	–	–
		[0.49, 0.55]	[0.16, 0.18]	[0.09, 0.11]	[0.20, 0.22]	–	–	–
	major (N=6)	0.94±0.04	0.43±0.01	0.20±0.01	0.51±0.01	–	–	–
		[0.89, 0.99]	[0.42, 0.44]	[0.20, 0.21]	[0.49, 0.53]	–	–	–
*chrislaini*	minor (N=5)	0.41±0.04	0.17±0.03	0.10±0.01	0.17±0.02	–	–	–
		[0.38, 0.42]	[0.16, 0.19]	[0.09, 0.10]	[0.17, 0.18]	–	–	–
	major (N=5)	0.82±0.03	0.49±0.02	0.20±0.01	0.43±0.01	–	–	–
		[0.80, 0.86]	[0.47, 0.52]	[0.19, 0.21]	[0.41, 0.45]	–	–	–
*claveri*	minor (N=8)	0.39±0.03	0.17±0.02	0.11±0.01	0.14±0.01	–	–	–
		[0.38, 0.42]	[0.16, 0.18]	[0.10, 0.12]	[0.14, 0.15]	–	–	–
	major (N=2)	0.84±0.01	0.45±0.01	0.22±0.01	0.40±0.00	–	–	–
		[0.83, 0.85]	[0.44, 0.46]	[0.21, 0.22]	[0.40, 0.40]	–	–	–
*darwinii*	minor (N=37)	0.46±0.05	0.18±0.02	0.10±0.02	0.18±0.03	–	–	–
		[0.41, 0.50]	[0.16, 0.20]	[0.09, 0.11]	[0.16, 0.20]	–	–	–
	major (N=14)	0.98±0.12	0.47±0.02	0.21±0.02	0.50±0.01	–	–	–
		[0.84, 1.28]	[0.43, 0.51]	[0.18, 0.23]	[0.47, 0.52]	–	–	–
*ellioti*	minor (N=42)	0.51±0.07	0.18±0.02	0.09±0.02	0.18±0.03	–	–	–
		[0.43, 0.56]	[0.16, 0.21]	[0.08, 0.10]	[0.16, 0.20]	–	–	–
	major (N=7)	0.75±0.07	0.43±0.02	0.15±0.02	0.46±0.02	–	–	–
		[0.61, 0.81]	[0.40, 0.46]	[0.11, 0.16]	[0.42, 0.50]	–	–	–
*maintilany*	minor (N=14)	0.41±0.02	0.17±0.01	0.11±0.01	0.15±0.01	–	–	–
		[0.40, 0.42]	[0.16, 0.18]	[0.11, 0.12]	[0.14, 0.15]	–	–	–
	major (N=3)	0.83±0.03	0.40±0.02	0.24±0.01	0.39±0.01	–	–	–
		[0.81, 0.86]	[0.39, 0.43]	[0.23, 0.25]	[0.38, 0.40]	–	–	–
**Species**	**Caste**	**CS**	**PoOC/CL**	**PrAC/CL**	**ClyL/CL**	**ClyL/GPD**	**CW/CL**	**CWb/CL**
*ivadia*	minor (N=4)	1.21±0.19	0.10±0.01	0.19±0.03	0.10±0.01	0.21±0.03	0.33±0.02	0.35±0.03
		[1.13, 1.30]	[0.10, 0.11]	[0.17, 0.20]	[0.09, 0.10]	[0.19, 0.22]	[0.32, 0.34]	[0.33, 0.36]
	major (N=1)	1.93	0.28	0.52	0.33	0.84	0.82	0.93
*jjacquia*	minor (N=10)	0.99±0.14	0.10±0.02	0.20±0.01	0.09±0.02	0.20±0.06	0.34±0.02	0.32±0.02
		[0.91, 1.09]	[0.09, 0.12]	[0.19, 0.20]	[0.07, 0.10]	[0.16, 0.23]	[0.33, 0.36]	[0.31, 0.34]
	major (N=4)	1.68±0.07	0.30±0.00	0.50±0.02	0.30±0.01	0.85±0.03	0.83±0.02	0.91±0.02
		[1.61, 1.76]	[0.29, 0.30]	[0.49, 0.53]	[0.29, 0.30]	[0.81, 0.88]	[0.82, 0.85]	[0.89, 0.92]
*norvigi*	minor (N=17)	1.45±0.24	0.08±0.01	0.23±0.01	0.12±0.01	0.26±0.03	0.33±0.02	0.36±0.02
		[1.31, 1.66]	[0.07, 0.09]	[0.21, 0.23]	[0.11, 0.13]	[0.23, 0.28]	[0.32, 0.34]	[0.35, 0.38]
	major (N=3)	1.98±0.30	0.23±0.01	0.56±0.01	0.30±0.01	0.72±0.02	0.88±0.02	0.99±0.01
		[1.78, 2.32]	[0.22, 0.23]	[0.56, 0.57]	[0.28, 0.31]	[0.69, 0.73]	[0.85, 0.89]	[0.98, 1.00]
*nossibeensis*	minor (N=50)	2.24±0.46	0.08±0.02	0.22±0.02	0.12±0.02	0.28±0.05	0.33±0.02	0.37±0.03
		[1.92, 2.70]	[0.06, 0.09]	[0.21, 0.24]	[0.11, 0.13]	[0.24, 0.32]	[0.32, 0.36]	[0.35, 0.42]
	major (N=18)	3.05±0.41	0.23±0.02	0.56±0.02	0.32±0.02	0.84±0.05	0.85±0.03	1.02±0.04
		[2.35, 3.67]	[0.18, 0.27]	[0.53, 0.61]	[0.29, 0.36]	[0.73, 0.91]	[0.81, 0.90]	[0.96, 1.08]
*radovae*	minor (N=17)	1.59±0.22	0.08±0.01	0.22±0.01	0.11±0.01	0.26±0.04	0.34±0.03	0.37±0.03
		[1.43, 1.77]	[0.07, 0.09]	[0.21, 0.23]	[0.10, 0.12]	[0.23, 0.29]	[0.33, 0.37]	[0.35, 0.40]
	major (N=4)	2.76±0.12	0.24±0.01	0.55±0.02	0.29±0.04	0.76±0.13	0.86±0.03	1.05±0.02
		[2.59, 2.85]	[0.23, 0.25]	[0.53, 0.59]	[0.23, 0.32]	[0.57, 0.86]	[0.82, 0.89]	[1.02, 1.07]
*ihazofotsy*	minor (N=2)	0.78±0.12	0.09±0.03	0.20±0.04	0.10±0.02	0.25±0.07	0.31±0.00	0.34±0.01
		[0.75, 0.81]	[0.08, 0.10]	[0.19, 0.21]	[0.10, 0.11]	[0.23, 0.27]	[0.31, 0.31]	[0.34, 0.35]
	major unknown							
*themistocles*	minor (N=11)	1.82±0.37	0.07±0.01	0.22±0.01	0.11±0.01	0.27±0.04	0.33±0.02	0.36±0.03
		[1.53, 1.98]	[0.07, 0.08]	[0.22, 0.23]	[0.10, 0.12]	[0.25, 0.30]	[0.31, 0.34]	[0.34, 0.38]
	major (N=6)	2.68±0.36	0.23±0.01	0.56±0.01	0.31±0.02	0.82±0.07	0.85±0.02	1.05±0.04
		[2.26, 3.15]	[0.22, 0.25]	[0.55, 0.58]	[0.28, 0.34]	[0.73, 0.93]	[0.82, 0.87]	[0.99, 1.11]
*tsimelahy*	minor (N=5)	0.79±0.04	0.09±0.00	0.19±0.01	0.09±0.02	0.23±0.04	0.33±0.02	0.29±0.02
		[0.77, 0.81]	[0.08, 0.09]	[0.19, 0.19]	[0.09, 0.10]	[0.21, 0.25]	[0.31, 0.34]	[0.28, 0.29]
	major unknown							
**Species**	**Caste**	**FR/CS**	**TCD/CS**	**SL/CS**	**MW/ML**	**PEW/CS**	**MPD/ML**	**NOH/CS**
*ivadia*	minor (N=4)	0.15±0.00	0.11±0.01	0.39±0.02	0.21±0.03	0.15±0.01	0.27±0.04	0.10±0.01
		[0.15, 0.16]	[0.11, 0.12]	[0.38, 0.40]	[0.19, 0.22]	[0.14, 0.15]	[0.25, 0.28]	[0.10, 0.11]
	major (N=1)	0.4	0.29	0.69	0.54	0.34	0.64	0.24
*jjacquia*	minor (N=10)	0.17±0.01	0.13±0.01	0.34±0.02	0.19±0.02	0.13±0.03	0.26±0.02	0.08±0.02
		[0.16, 0.18]	[0.12, 0.14]	[0.32, 0.34]	[0.18, 0.20]	[0.11, 0.14]	[0.24, 0.26]	[0.07, 0.09]
	major (N=4)	0.43±0.01	0.29±0.01	0.57±0.02	0.54±0.02	0.28±0.02	0.61±0.01	0.20±0.02
		[0.42, 0.44]	[0.27, 0.30]	[0.56, 0.59]	[0.51, 0.56]	[0.25, 0.30]	[0.60, 0.62]	[0.18, 0.23]
*norvigi*	minor (N=17)	0.15±0.01	0.12±0.01	0.40±0.03	0.22±0.02	0.12±0.01	0.23±0.01	0.11±0.02
		[0.14, 0.15]	[0.11, 0.13]	[0.37, 0.42]	[0.21, 0.23]	[0.11, 0.13]	[0.22, 0.24]	[0.10, 0.13]
	major (N=3)	0.35±0.01	0.28±0.01	0.90±0.14	0.55±0.02	0.30±0.03	0.60±0.05	0.26±0.03
		[0.34, 0.36]	[0.27, 0.29]	[0.75, 1.00]	[0.54, 0.57]	[0.27, 0.33]	[0.57, 0.65]	[0.22, 0.28]
*nossibeensis*	minor (N=50)	0.14±0.01	0.11±0.01	0.39±0.05	0.22±0.03	0.14±0.02	0.24±0.04	0.12±0.02
		[0.13, 0.15]	[0.10, 0.12]	[0.33, 0.41]	[0.20, 0.24]	[0.12, 0.17]	[0.21, 0.28]	[0.10, 0.14]
	major (N=18)	0.34±0.01	0.27±0.01	0.77±0.08	0.55±0.02	0.33±0.02	0.64±0.04	0.26±0.04
		[0.33, 0.35]	[0.25, 0.28]	[0.68, 0.91]	[0.52, 0.57]	[0.30, 0.39]	[0.54, 0.70]	[0.21, 0.34]
*radovae*	minor (N=17)	0.14±0.01	0.11±0.01	0.39±0.03	0.21±0.02	0.13±0.02	0.24±0.02	0.11±0.03
		[0.13, 0.15]	[0.10, 0.11]	[0.37, 0.41]	[0.20, 0.23]	[0.11, 0.14]	[0.22, 0.26]	[0.10, 0.13]
	major (N=4)	0.33±0.00	0.26±0.01	0.74±0.03	0.55±0.02	0.32±0.01	0.65±0.04	0.27±0.02
		[0.33, 0.33]	[0.24, 0.27]	[0.72, 0.79]	[0.52, 0.58]	[0.31, 0.33]	[0.61, 0.69]	[0.25, 0.29]
*ihazofotsy*	minor (N=2)	0.15±0.02	0.10±0.00	0.35±0.03	0.18±0.01	0.09±0.01	0.25±0.01	0.06±0.00
		[0.14, 0.15]	[0.10, 0.10]	[0.34, 0.36]	[0.17, 0.18]	[0.09, 0.09]	[0.25, 0.26]	[0.06, 0.06]
	major unknown							
*themistocles*	minor (N=11)	0.13±0.01	0.10±0.01	0.41±0.05	0.21±0.02	0.14±0.02	0.24±0.03	0.13±0.03
		[0.12, 0.14]	[0.09, 0.10]	[0.38, 0.44]	[0.20, 0.22]	[0.13, 0.15]	[0.22, 0.26]	[0.11, 0.15]
	major (N=6)	0.30±0.06	0.25±0.01	0.75±0.03	0.55±0.01	0.33±0.02	0.63±0.05	0.25±0.02
		[0.19, 0.34]	[0.23, 0.27]	[0.71, 0.79]	[0.52, 0.56]	[0.30, 0.36]	[0.59, 0.70]	[0.22, 0.28]
*tsimelahy*	minor (N=5)	0.14±0.02	0.10±0.01	0.41±0.03	0.19±0.02	0.12±0.01	0.27±0.01	0.08±0.01
		[0.13, 0.15]	[0.09, 0.10]	[0.40, 0.42]	[0.17, 0.20]	[0.12, 0.13]	[0.26, 0.27]	[0.07, 0.08]
	major unknown							
**Species**	**Caste**	**HTL/CS**	**OMD/CS**	**EL/CS**	**MPH/ML**			
*ivadia*	minor (N=4)	0.39±0.03	0.17±0.02	0.10±0.02	0.16±0.01	–	–	–
		[0.38, 0.40]	[0.16, 0.18]	[0.09, 0.11]	[0.16, 0.17]	–	–	–
	major (N=1)	0.86	0.44	0.2	0.45	–	–	–
*jjacquia*	minor (N=10)	0.35±0.05	0.18±0.03	0.10±0.02	0.13±0.02	–	–	–
		[0.31, 0.38]	[0.16, 0.19]	[0.09, 0.11]	[0.12, 0.14]	–	–	–
	major (N=4)	0.69±0.03	0.42±0.02	0.19±0.00	0.35±0.01	–	–	–
		[0.67, 0.73]	[0.40, 0.44]	[0.19, 0.20]	[0.34, 0.36]	–	–	–
*norvigi*	minor (N=17)	0.48±0.04	0.19±0.02	0.09±0.01	0.18±0.01	–	–	–
		[0.45, 0.51]	[0.18, 0.21]	[0.09, 0.10]	[0.18, 0.20]	–	–	–
	major (N=3)	1.12±0.13	0.51±0.03	0.21±0.01	0.46±0.01	–	–	–
		[0.98, 1.20]	[0.49, 0.54]	[0.19, 0.22]	[0.44, 0.46]	–	–	–
*nossibeensis*	minor (N=50)	0.51±0.06	0.19±0.02	: 0.09±0.02	0.21±0.05	–	–	–
		[0.46, 0.56]	[0.17, 0.21]	[0.07, 0.10]	[0.18, 0.24]	–	–	–
	major (N=18)	1.03±0.11	0.48±0.02	0.20±0.01	0.53±0.03	–	–	–
		[0.90, 1.23]	[0.45, 0.50]	[0.18, 0.23]	[0.49, 0.57]	–	–	–
*radovae*	minor (N=17)	0.53±0.05	0.19±0.02	0.09±0.01	0.18±0.02	–	–	–
		[0.50, 0.57]	[0.18, 0.20]	[0.09, 0.10]	[0.17, 0.20]	–	–	–
	major (N=4)	1.00±0.02	0.51±0.02	0.20±0.01	0.47±0.01	–	–	–
		[0.98, 1.03]	[0.49, 0.53]	[0.19, 0.21]	[0.46, 0.48]	–	–	–
*ihazofotsy*	minor (N=2)	0.37±0.01	0.16±0.01	0.11±0.00	0.10±0.01	–	–	–
		[0.37, 0.38]	[0.16, 0.17]	[0.11, 0.11]	[0.10, 0.10]	–	–	–
	major unknown					–	–	–
*themistocles*	minor (N=11)	0.50±0.04	0.19±0.03	0.09±0.01	0.19±0.02	–	–	–
		[0.47, 0.54]	[0.17, 0.21]	[0.09, 0.11]	[0.18, 0.20]	–	–	–
	major (N=6)	0.91±0.04	0.46±0.03	0.20±0.01	0.51±0.03	–	–	–
		[0.86, 0.98]	[0.44, 0.50]	[0.19, 0.21]	[0.48, 0.55]	–	–	–
*tsimelahy*	minor (N=5)	0.48±0.01	0.19±0.01	0.12±0.01	0.13±0.01	–	–	–
		[0.48, 0.49]	[0.18, 0.19]	[0.12, 0.13]	[0.13, 0.14]	–	–	–
	major unknown							
**Species**	**Caste**	**CS**	**PoOC/CL**	**PrAC/CL**	**ClyL/CL**	**ClyL/GPD**	**CW/CL**	**CWb/CL**
*ursus*	minor (N=5)	1.54±0.19	0.07±0.02	0.23±0.02	0.11±0.02	0.25±0.03	0.34±0.02	0.37±0.02
		[1.43, 1.64]	[0.06, 0.08]	[0.22, 0.24]	[0.10, 0.12]	[0.22, 0.26]	[0.33, 0.35]	[0.36, 0.38]
	major (N=6)	2.14±0.15	0.23±0.01	0.57±0.01	0.39±0.16	1.03±0.41	1.01±0.08	0.89±0.07
		[2.00, 2.39]	[0.21, 0.24]	[0.56, 0.59]	[0.29, 0.69]	[0.73, 1.78]	[0.88, 1.12]	[0.84, 1.03]
*zoro*	minor (N=3)	1.02±0.28	0.09±0.01	0.22±0.02	0.11±0.00	0.24±0.01	0.38±0.00	0.39±0.01
		[0.92, 1.14]	[0.08, 0.09]	[0.22, 0.23]	[0.11, 0.11]	[0.24, 0.24]	[0.38, 0.38]	[0.39, 0.40]
	major (N=2)	1.74±0.04	0.26±0.01	0.53±0.01	0.29±0.04	0.74±0.05	0.92±0.02	0.98±0.02
		[1.71, 1.77]	[0.25, 0.26]	[0.53, 0.54]	[0.26, 0.31]	[0.70, 0.77]	[0.90, 0.93]	[0.97, 1.00]
**Species**	**Caste**	**FR/CS**	**TCD/CS**	**SL/CS**	**MW/ML**	**PEW/CS**	**MPD/ML**	**NOH/CS**
*ursus*	minor (N=5)	0.14±0.01	0.11±0.01	0.38±0.02	0.21±0.02	0.13±0.01	0.24±0.07	0.12±0.02
		[0.13, 0.15]	[0.11, 0.12]	[0.37, 0.39]	[0.20, 0.22]	[0.13, 0.13]	[0.21, 0.29]	[0.11, 0.13]
	major (N=6)	0.37±0.02	0.29±0.02	0.82±0.03	0.56±0.02	0.32±0.03	0.60±0.03	0.29±0.03
		[0.34, 0.40]	[0.26, 0.30]	[0.78, 0.86]	[0.54, 0.58]	[0.27, 0.37]	[0.56, 0.64]	[0.26, 0.33]
*zoro*	minor (N=3)	0.15±0.01	0.12±0.00	0.34±0.04	0.21±0.01	0.13±0.01	0.25±0.02	0.10±0.03
		[0.15, 0.16]	[0.12, 0.12]	[0.33, 0.36]	[0.21, 0.22]	[0.13, 0.14]	[0.24, 0.25]	[0.08, 0.11]
	major (N=2)	0.38±0.00	0.31±0.01	0.66±0.02	0.54±0.00	0.27±0.00	0.64±0.02	0.23±0.01
		[0.38, 0.38]	[0.30, 0.31]	[0.65, 0.68]	[0.54, 0.54]	[0.27, 0.28]	[0.63, 0.65]	[0.22, 0.23]
**Species**	**Caste**	**HTL/CS**	**OMD/CS**	**EL/CS**	**MPH/ML**			
*ursus*	minor (N=5)	0.46±0.03	0.19±0.02	0.09±0.01	0.18±0.02	–	–	–
		[0.45, 0.48]	[0.18, 0.20]	[0.09, 0.10]	[0.16, 0.19]	–	–	–
	major (N=6)	1.01±0.06	0.51±0.02	0.21±0.01	0.46±0.03	–	–	–
		[0.91, 1.06]	[0.47, 0.54]	[0.19, 0.22]	[0.41, 0.50]	–	–	–
*zoro*	minor (N=3)	0.36±0.02	0.19±0.01	0.08±0.01	0.18±0.02	–	–	–
		[0.35, 0.37]	[0.19, 0.19]	[0.08, 0.09]	[0.17, 0.18]	–	–	–
	major (N=2)	0.79±0.01	0.45±0.01	0.21±0.01	0.42±0.01	–	–	–
		[0.78, 0.80]	[0.44, 0.46]	[0.21, 0.22]	[0.42, 0.42]	–	–	–

**BLF** BL Fisher and the Madagascar Biodiversity Center team

**FGAT** Fisher-Griswold Arthropod Team

**ARA** Andrianjaka Ravelomanana

**PSW** Phil Ward

**MG** Madagascar Malaise trap program by Mike Irwin and Rasolondalao Harin’hala Hasinjaka

### ﻿Imaging

Digital color montage images were created using a JVC KY-F75 digital camera and syncroscopy Auto-Montage software (version 5.0), or a Leica DFC 425 camera in combination with the Leica Application Suite software (version 3.8). Distribution maps were generated by using QGIS 2.4.0 software (QGIS Development Team 2014).

### ﻿Morphometric character recording and terminology

**Figure 1.**
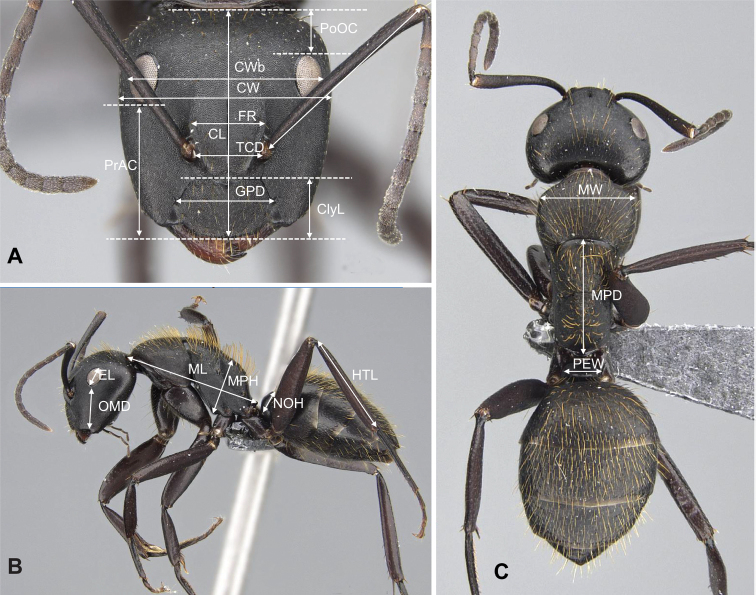
Illustration of measurements of ants in the subgenus Mayria**A** head in full-face view **B** body in lateral view **C** body in dorsal view.

Measurements were taken with a Leica MZ 9.5 stereomicroscope equipped with a cross-scaled ocular micrometer. Each worker was evaluated using 19 continuous morphometric traits, measured as in Rakotonirina et al. (2016) for the *Camponotusedmondi* group. The morphometric data are expressed in mm. The following measurements were used (see Figure 1):

**CL** Maximum cephalic length. The maximum median length of the head in full-face view, measured from the midpoint of the posterior margin of head to the midpoint of the anterior margin of the clypeus (Fig. 1A).

**ClyL** Clypeal length. The maximum median length of the clypeus measured from the posterior margin to the anterior margin in frontal view, in which the anterior and posterior clypeal margins are aligned to the same focus. Median concavity on either or both margins reduces the length of the clypeus (Fig. 1A).

**CS** Cephalic size. This derived character is used as a body size indicator and is calculated from the arithmetic mean of head length (CL) and maximum head capsule width (CWb).

**CW** Maximum cephalic width. The longest distance between the lateral margins of the compound eyes in full-face view (Fig. 1A).

**CWb** Maximum head capsule width. The maximum width of the head capsule excluding the compound eyes (Fig. 1A).

**EL** Eye length. Maximum diameter of the compound eye (Fig. 1B).

**FR** Frontal carina distance. Longest distance between the frontal carina (Fig. 1A).

**GPD** Maximum tentorial pit distance. The longest distance between the centers of the fossae located at or very close to the posterolateral margin of the clypeus (Fig. 1A).

**HTL** Maximum hind tibia length. Straight line length of the hind tibia measured from the constriction immediately before its proximal insertion to its distalmost point, excluding the bristles or spines (Fig. 1B).

**ML** Mesosoma length. The longest anatomical line that connects the posteriormost point of the propodeal lobe with the anteriormost point of the pronotal collar; preferentially measured in lateral view, but if one of the reference points is not visible, dorsal view may help (Fig. 1B).

**MPD** Mesothoracico-propodeal distance. With the promesonotal suture and the anterior petiolar foramen margin in the same plane of focus in dorsal view, the maximum length between the promesonotal suture and the posteriormost point of the propodeal process dorsal to the petiolar insertion (Fig. 1C).

**MPH** Mesothoracico-propodeal height. Measured in lateral view, using as a reference the diagonal line that connects the anteriormost point of the pronotal shield and the posteriormost point of the propodeal process dorsal to the petiolar insertion. MPH is the perpendicular distance between two lines parallel to this line, one of which just touches the anteroventral corner of the mesopleuron, dorsal to the insertion of the mesocoxa, and the other which touches the dorsalmost point of the propodeum (Fig. 1B).

**MW** Mesosoma width. Maximum width of the pronotum in dorsal view (Fig. 1C).

**NOH** Petiolar node height. The maximum distance between the petiolar spiracle and the dorsalmost point of the petiolar node (Fig. 1B).

**OMD** Oculo-mandibular distance. The minimum distance between the anterior margin of the compound eye and the mandibular insertion to the head (Fig. 1B).

**PEW** Petiolar width. The maximum width of the petiole in dorsal view (Fig. 1C).

**PoOC** Postocular distance. The longest distance between the posteromedian margin of the head and theposterior margins of the two compound eyes. Measured at a right angle to the reference line in full-face view (Fig. 1A).

**PrAC** Preocular distance. The longest distance between the anteromedian margin of the clypeus and the level of the anterior margin of the compound eyes as a reference line. Measured at a right angle to the reference line in full-face view (Fig. 1A).

**SL** Scape length. Straight line length of the first antennal segment excluding the basal condyle (Fig. 1B).

**TCD** Torular carina distance. The minimum distance between the torular arches that surround the antennal insertion (Fig. 1A).

### ﻿Morphometric data analysis

After measuring workers (240 minors and 67 majors), multivariate statistical analysis based on the protocol described by Seifert et al. (2014) and extended by Csősz and Fisher (2016) was performed with data from minor workers only, since *Camponotus* workers exhibit different forms of allometry. Species hypotheses were generated using the NC-clustering technique (Seifert et al. 2014) via the packages CLUSTER (Maechler et al. 2014) and MASS (Venables and Ripley 2002). The procedure followed Csősz and Fisher (2016) and Rakotonirina et al. (2017, 2018).

## ﻿Results and discussion

### The subgenus Mayria Forel

*Mayria* Forel, 1878: 369. [As subgenus of *Camponotus* Forel, 1894: 227; Bolton, 1994: 50]. Type species: *Camponotusrepens* Forel, 1897: 187, replacement name for *Mayriamadagascarensis* Forel, 1886c: 4, a junior homonym of *Camponotusniveosetosusmadagascarensis* Forel, 1886c: 4].

*Mayria* as genus Dalle Torre, 1893: 219; Asheamed, 1905c: 384.

*Mayria* as subgenus of *Camponotus* Forel, 1894e: 227; Forel, 1914a: 262; Forel, 1927: 251; Wheeler, 1922: 706; Emery, 1925d: 1121; subsequent authors: Bolton, 1995b: 34; Bolton, 2003: 113.

*Mayria* as junior synonym of *Myrmosaga* Forel, 1912.

*Myrmepinotus* Santschi, 1921: 312. [As subgenus of *Camponotus*]. Type species: *Camponotusechinoploides* Forel, 189. Syn. nov.

**Figure 2.**
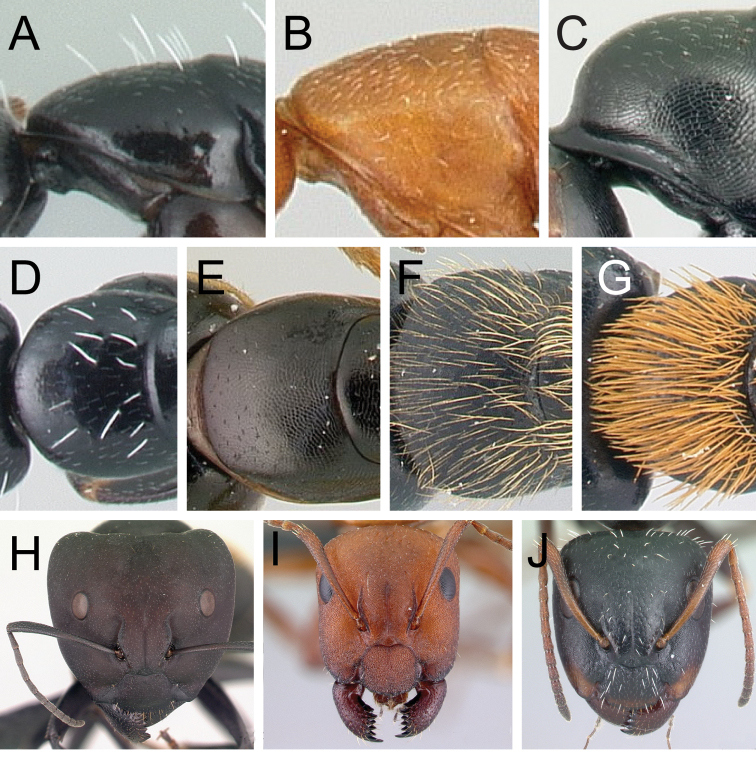
Morphological characters of workers of the subgenus Mayria. Pronotum in lateral view **A***C.repens* (CASENT0217300) **B***C.maintikibo* (CASENT0062631) **C***C.alamaina* (CASENT0481799) Pronotal disc in dorsal view **D***C.repens* (CASENT0217300) **E***C.bevohitra* (CASENT0437238) **F***C.nossibeensis* (CASENT0191657) **G***C.ursus* (CASENT0217284) head of major in full-face view **H***C.ellioti* (CASENT0450893) **I***C.andrianjaka* (CASENT0159208) **J***C.repens* (CASENT0491956).

**Diagnosis.** Workers of the subgenus Mayria are easily distinguished from the other subgenera in Madagascar by the following: postocular distance shorter (PoOC/CL< 0.12); clypeus with short median lobe, its anterolateral corner rounded; anterior margin of pronotum strongly convex in lateral view, forming a rounded flange and extended laterally to form an obtuse humeral angle (Fig. 2A–C); in dorsal view, pronotal disc rectangular with distinct lateral margin (Fig. 2D–F); propodeum dorsum never concave; posterior margin of petiolar node relatively sharp and always surmounted with standing hairs. Body integument opaque, sculptured, and generally matte in color. Standing setae consist of straplike hairs, at least thickened basally (Fig. 2F, G).

**Diagnosis of minor worker.** Head of minor trapezoidal, broadened posteriorly, lateral cephalic margins slightly convex to straight, occipital margin almost straight to evenly convex (Fig. 2H–J). Mandible short and triangular, armed with six teeth, basal margin forming a smooth curve, not forming a right angle with the mandible insertion. Palp formula: 6, 4. Maxillary palp long. Clypeus transversely trapezoidal with rounded to evenly triangular or rectangular anterior margin, anterolateral portion always rounded, posterior margin of clypeus straight. Antennae 12-segmented: moderately long, scape gradually broadened apically. Frontal lobe narrowed anteriorly, wider behind the antennal insertion; frontal carina V-shaped. Compound eye moderately sized, protruding, breaking the outline of the lateral cephalic margin in *alamaina* species group, located posterior to the midlength of the head.

Mesosoma in lateral view, dorsal outline of mesosoma continuous (*darwinii* group) to complex (other groups). Promesonotal suture visible. Pronotum: humeral angle tuberculate (*C.ellioti* species group), marginate in the remaining species group; in dorsal view, pronotal disc usually rectangular. Mesonotum in dorsal view, wider than long; laterally marginate in *C.edmondi* species group. Mesopleuron and propodeal surface together distinctly longer than lateral portion of pronotum in lateral view. Propodeal lobe reduced. Metapleural gland absent. Procoxa of normal to moderate size for *C.edmondi* species group. Middle and hind tibiae with single pectinate spur.

Petiolar node: broad and massive, ranging from squamiform to nodiform, posterodorsal edge of petiole always marginate and ornamented with thick, erect hairs.

Integument opaque and sculptured, ranging from finely and densely reticulate-punctate to finely and densely imbricate.

Pilosity: standing setae thick at least at the base, whitish to pale colored, erect to appressed hairs, filiform to spatulate hairs, moderately distributed on entire dorsum of head and body; ground pilosity always present and conspicuous.

**Diagnosis of major worker.** Most of the morphological traits cited above for minor workers are also characteristics of major workers. However, major workers exhibit the following characters: head more subquadrate to rectangular; malar area thimble-like with weak impression at the base of each seta (Fig. 2K–M); lateral portion of head sculptured as finely and densely reticulate-punctate, imbricate, or areolate, and superimposed with two to seven smaller areoles embedded in scattered large punctures, from which an appressed hair arises medially; antennal scape shorter, its apex barely surpassing or not extending beyond posterior cephalic margin; dorsal outline of mesosoma with the same structure as minor worker.

**Note.** The type species *Camponotusechinoploides* of the subgenus Myrmepinotus shares the characters of the *Mayria* diagnosis and therefore *Myrmepinotus* is synonymized with *Mayria.* The following characters place *C.echinoploides* within the *Mayria* subgenus: in dorsal view, mesosoma relatively short and distinctly marginate anteriorly and laterally; pronotum and mesonotum widened; promesonotal and metanotal sutures well marked; in profile, petiolar node thick with convex anterior and posterior margins, and body covered with whitish, thick hairs. In fact, the lectotype of *C.repens* and the holotype of *C.echinoploides* share a suite of morphological characters: in full-face view, head elongate, broadened posteriorly with slightly convex sides; clypeus with short median lobe; in profile, anterior margin of pronotum strongly convex; pronotal disc subrectangular, marginate laterally; petiolar node as high as long; standing setae consist of thick, whitish hairs; body integument dull. These morphological characters are observed also in specimens belonging to the *edmondi* species group but absent for species that are removed from the subgenus Mayria (Table 2). Accordingly, we have treated *Myrmepinotus* as a synonym of *Mayria*.

**Table 2.** Summary of taxonomic changes within the subgenus Mayria.

Taxon	Change	Notes	Proposed classification
*Myrmepinotus*	syn. nov.		Junior synonym of *Mayria*
*Camponotusechinoploides*	subgenus	Previously in *Myrmepinotus*	Placed in *Mayria* under *edmondi* species group
*Camponotusedmondi*			
*Camponotusethicus*			
*Camponotusrobustus*			
*Camponotusdarwinii*	subgenus	Previously in *Myrmopiromis*	Placed in *Mayria* under *darwinii* species group
*Camponotusdescarpentriesi*			
*Camponotusellioti*			
*Camponotusmadagascarensis*			
*Camponotusnossibeensis*			
*Camponotusradovae*			
*Camponotusthemistocles*			
*Camponotusursus*			
*Camponotusvoeltzkowii*			
*Camponotuschristi*	subgenus	Previously in *Mayria*	Placed in *Myrmonesites*
*Camponotusdromedaries*			
*Camponotusfoersteri*			
*Camponotuslamosy*			
*Camponotusmaculiventris*			
*Camponotusmainty*			
*Camponotusmanabo*			
*Camponotuspulcher*			
*Camponotusraina*			
*Camponotussada*			
*Camponotustanosy*			
*Camponotuslubbocki*	subgenus	Previously in *Mayria*	Placed in *Myrmosaga*

**Table 3.** Identification matrix of species showing the identification success (percentage), the observed identification (rows), and the predicted identification (columns) based on Leave One Out Cross Validation LDA. Numbers in the matrix are specimen counts.

Observed species	predicted species																	
	*nossibeensis*	*jjacquia*	*themistocles*	*darwinii*	*ellioti*	*claveri*	*zoro*	*radovae*	*ihazofotsy*	*tsimelahy*	*norvigi*	*ursus*	*chrislaini*	*maintilany*	*antsaraingy*	*ivadia*	*andrianjaka*	Identification success (%)
*nossibeensis*	45	–	–	–	–	–	–	–	–	–	–	–	–	–	–	–	–	100
*jjacquia*	–	1	–	–	–	–	–	–	–	–	–	–	–	–	–	–	–	100
*themistocles*	–	–	11	–	–	–	–	–	–	–	–	–	–	–	–	–	–	100
*darwinii*	–	–	–	35	–	–	–	–	–	–	–	–	–	–	–	–	–	100
*ellioti*	–	–	–	–	42	–	–	–	–	–	–	–	–	–	–	–	–	100
*claveri*	–	–	–	–	–	8	–	–	–	–	–	–	–	–	–	–	–	100
*zoro*	–	–	–	–	–	–	3	–	–	–	–	–	–	–	–	–	–	100
*radovae*	–	–	–	–	–	–	–	17	–	–	–	–	–	–	–	–	–	100
*ihazofotsy*	–	–	–	–	–	–	–	–	2	–	–	–	–	–	–	–	–	100
*tsimelahy*	–	–	–	–	–	–	–	–	–	5	–	–	–	–	–	–	–	100
*norvigi*	–	–	–	–	–	–	–	–	–	–	17	–	–	–	–	–	–	100
*ursus*	–	–	–	–	–	–	–	–	–	–	–	7	–	–	–	–	–	100
*chrislaini*	–	–	–	–	–	–	–	–	–	–	–	–	5	–	–	–	–	100
*maintilany*	–	–	–	–	–	–	–	–	–	–	–	–	–	14	–	–	–	100
*antsaraingy*	–	–	–	–	–	–	–	–	–	–	–	–	–	–	6	–	–	100
*ivadia*	–	–	–	–	–	–	–	–	–	–	–	–	–	–	–	4	–	100
*andrianjaka*	–	–	–	–	–	–	–	–	–	–	–	–	–	–	–	–	9	100
Total	45	1	11	35	42	8	3	17	2	5	17	7	5	14	6	4	9	100

### Synoptic list of the 39 species of CamponotussubgenusMayria

*alamaina* species group:

*alamaina* Rakotonirina, Csősz & Fisher, 2016

*androy* Rakotonirina, Csősz & Fisher, 2016

*bevohitra* Rakotonirina, Csősz & Fisher, 2016

*antsaraingy* species group:

*antsaraingy* sp. nov.

*darwinii* species group:

*darwinii* Forel, 1886

= Camponotusdarwiniivar.rubropilosus Forel, 1891: 44. syn. nov.

= Camponotusdarwiniir.rubropilosusvar.robustior Forel, 1905: 165. syn. nov.

*norvigi* sp. nov.

*nossibeensis* André, 1887

*radovae* Forel, 1886

= *Camponotusradovaedarwinii* Forel, 1891: 46. syn. nov.

*themistocles* Forel, 1910, stat. nov.

*ursus* Forel, 1886

*edmondi* species group:

*echinoploides* Forel, 1891

*edmondi* André, 1887

= *edmondi* var. ernesti Forel, 1891 syn. nov.

*galoko* Rakotonirina, Csősz & Fisher, 2016

*matsilo* Rakotonirina, Csősz & Fisher, 2016

*mifaka* Rakotonirina, Csősz & Fisher, 2016

*orombe* Rakotonirina, Csősz & Fisher, 2016

*tafo* Rakotonirina, Csősz & Fisher, 2016

*tratra* Rakotonirina, Csősz & Fisher, 2016

*varatra* Rakotonirina, Csősz & Fisher, 2016

*zavo* Rakotonirina, Csősz & Fisher, 2016

*efitra* species group:

*chrislaini* sp. nov.

*efitra* Rakotonirina, Csősz & Fisher, 2017

*ellioti* species group:

*andrianjaka* sp. nov.

*ellioti* Forel, 1891

*maintikibo* Rakotonirina, Csősz & Fisher, 2017

*maintilany* sp. nov.

*madagascarensis* species group:

*descarpentriesi* Santschi, 1926

*ivadia* sp. nov.

*madagascarensis* Forel, 1886

*mita* Rakotonirina, Csősz & Fisher, 2017

*voeltzkowii* Forel, 1894

*repens* species group:

*jjacquia* sp. nov.

*claveri* sp. nov.

*repens* Forel, 1897

*ihazofotsy* sp. nov.

*tsimelahy* sp. nov.

*robustus* species group:

*ethicus* Forel, 1897

*robustus* Roger, 1863

*zoro* sp. nov.

### Definition of the *Camponotusalamaina* species group

**Figure 3.**
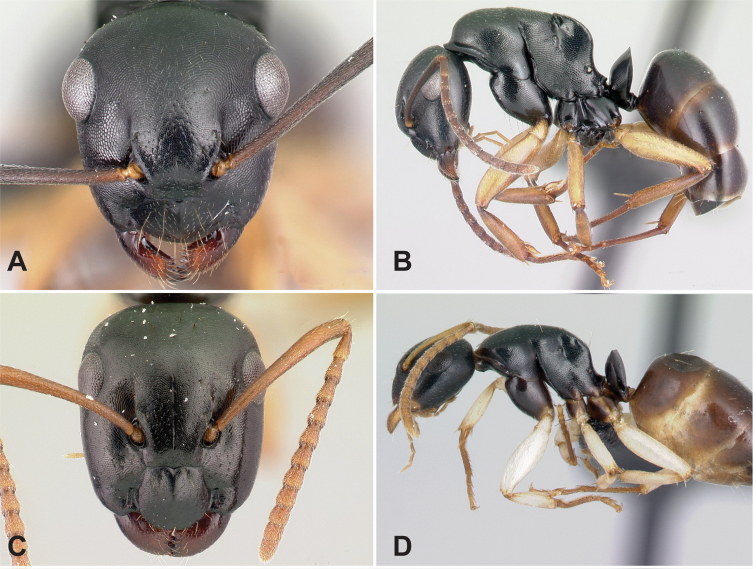
Morphological character of minor and major workers of *C.alamaina* species group **A, B** head in full-face view and body in lateral view of *C.alamaina* (CASENT0481799) **C** head in full-face view of major worker of *C.bevohitra* (CASENT0437238) **D** body in lateral view of *C.androy* (CASENT0453723).

**Minor worker**: in full-face view, head subovate, distinctly longer than broad, lateral margin more or less straight and rounding to the broadly convex posterior margin (Fig. 3A). Anteromedian margin of clypeus generally convex; posteromedian margin slightly notched.

**Major worker**: in full-face view, head subquadrate, posterior margin approximately straight and rounding to lateral margins. Clypeus hexagonal, its anterior border straight and medially excised, frontal triangle distinct (Fig. 3C).

**Minor and major workers**: Mesosomal profile almost flattened and interrupted by the impressed metanotal suture. Promesonotal dorsum flattened; anterodorsal angle of pronotum and dorsolateral portion of mesosoma bluntly marginate; posterolateral margin of propodeum rounding to declivitous surface (Fig. 3B–D). In dorsal view, mesonotum as long as broad, but sides converging posteriorly; mesonotum and propodeum laterally compressed at their junction; metanotal groove vestigial, represented by a transverse line. In profile, petiolar node anteroposteriorly flattened and tapered dorsally; anterior margin slightly convex and posterior margin more or less straight; dorsal margin straight or weakly excised medially (Fig. 3D). Dorsum of head, mesosoma, and petiole with imbricate sculpture; gaster with finer imbrication; mandible coriarious-puncticulate. Pairs of erect hairs transversely arranged on mesosomal dorsum and in a row on gastral tergite, pubescence short and scattered on dorsum of body.

**Remarks.** The group is distinguishable from all other members of the subgenus Mayria by the combination of the following characters: mesosoma distinctly marked with promesonotal and metanotal sutures; petiole with sharp edge, its anterior margin convex and its posterior margin more or less straight; propodeal spiracle placed anterior to posterolateral margin of propodeum; head and mesosoma black to dark brown, gaster and appendages dark brown to yellow or depigmented yellow; cervical shield joining pronotal dorsum directly; junction of dorsal face to lateral face of propodeum without sharp carina, posterolateral margin present.

Even though Rakotonirina et al. (2016) described these three species as members of *C.edmondi* species group, these character states justify a different grouping. In addition, species of this group prefer arid habitats such as dry forest, spiny bush and thicket, and gallery forest, with the exception of *Camponotusbevohitra*, which can be found in montane rainforest.

### Definition of the *Camponotusantsaraingy* species group

**Figure 4.**
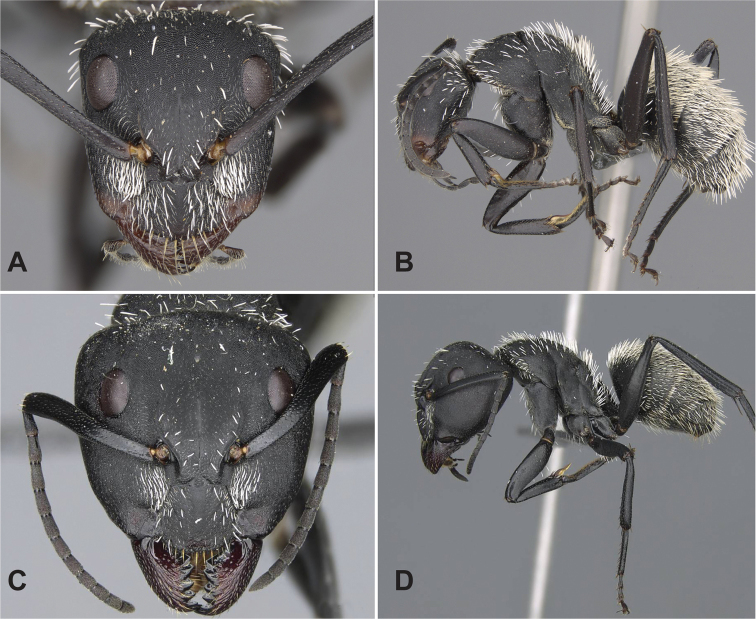
Morphological character of minor and major workers of *C.antsaraingy* species group *Camponotusantsaraingy***A, B** head in full-face view and mesosoma in lateral view of minor worker (CASENT0371032) **C, D** head in full-face view and mesosoma in lateral view of major worker (CASENT0840640).

**Minor worker**: in full-face view, head subtriangular, longer than wide, with arched posterior margin and straight lateral sides. Clypeus with projecting triangular lobe (Fig. 4A).

**Major worker**: in full-face view, head large, about as broad as long, with slightly convex posterior and lateral borders. Anterior margin of clypeus forming a rounded lobe (Fig. 4C).

**Minor and major workers**: antennal scape entirely flattened longitudinally. In lateral view, mesosoma short and high, propodeal dorsum relatively shorter than declivitous face. Suberect spatulate hairs present on occipital portion until anterior margin of eyes and on clypeal dorsum, pubescence long and distinct, moderately abundant next to antennal insertion. Body massive, black throughout. In lateral view, petiole cuneiform, with sharp dorsal margin. Body integument matte black, finely and densely reticulate-punctate. Dorsum of mesosoma and gaster covered with numerous spatulate, whitish hairs (Fig. 4B, D).

**Remarks.***Camponotusantsaraingy* is placed in its own group, based mainly on the following characters: its large size, the shape of its antennal scape, propodeum distinctly reduced, its dorsum covered with erect spatulate setae, and having only three gastral tergites visible in dorsal view. It is one of the groups in the subgenus Mayria with white pilosity. It differs from the *madagascarensis* species group by the structure of its mesosoma and its larger size.

### Definition of the *Camponotusdarwinii* species group

**Figure 5.**
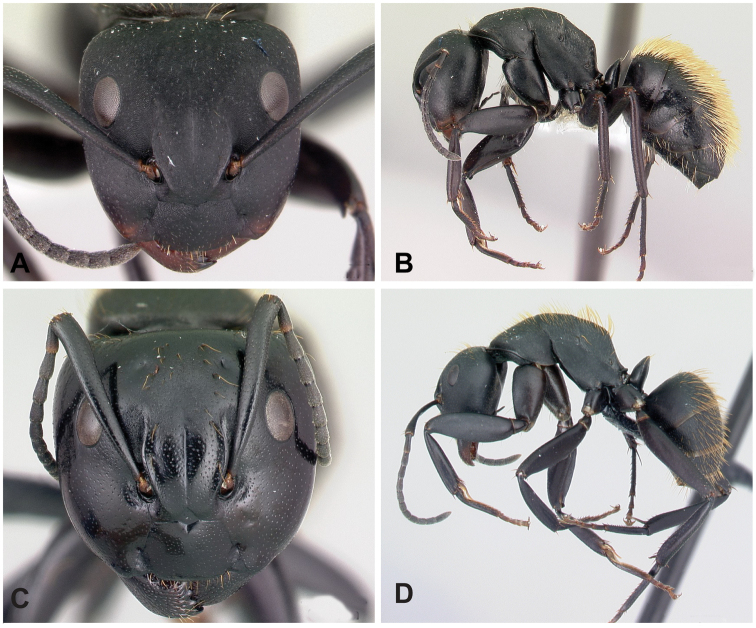
Morphological character of minor and major workers of *C.darwinii* species group **A** head of minor worker in full-face view of *C.darwinii* (CASENT0179460) **B** body in lateral view of the same species **C** head of major worker in full-face view *C.nossibeensis* (CASENT0493593) **D** body in lateral view of *C.themistocles* (CASENT0217297).

**Minor worker**: in full-face view, head more or less rectangular, with convex lateral sides and evenly straight occipital margin. Setae on dorsum of head sparsely distributed on occipital portion, arranged in longitudinal row along frontal carina (Fig. 5A).

**Major worker**: in full-face view, head somewhat longer than broad, a little narrower in front than broad (Fig. 5C).

**Minor and major worker**: clypeus with short, truncated, or rounded rectangular median lobe, its dorsum covered with randomly spaced setae. Mesosomal profile continuous, promesonotal and metanotal suture not impressed, sometimes forming a shiny line. Petiole scale usually not thick, but thin or sharp along margins. Integument opaque, matte black or brown, distinctly reticulate-punctate throughout. Body mostly covered with coarse yellow, red, or white setae, sometimes forming a dense fur coating the gaster or mesosoma (Fig. 5B, D).

**Remarks.***Camponotusdarwinii* species group has consistently been considered a distinctive group because of the presence of coarser and denser pubescence on its dorsal margin; integument dull and sculptured; dorsal outline of mesosoma continuous. This group is distinguishable from all other members of the subgenus Mayria by the presence of dense, whitish pilosity on its dorsum and the arcuate profile of its mesosoma. Species in this group are traditionally classified under subgenus Myrmopiromis, type species *Camponotusfulvopilosus*, type location South Africa. The subgeneric definition by Emery (1925) and the revised definition of *Camponotusfulvopilosus* species group by Robertson (1990, 1997) are quite different from *Camponotusdarwinii* species group. In addition to the species distribution, the number of mandibular teeth is a good character for distinguishing these two species groups: six for *C.darwinii* group and seven or eight for *C.fulvopilosus* group.

### Definition of the *Camponotusedmondi* species group

**Figure 6.**
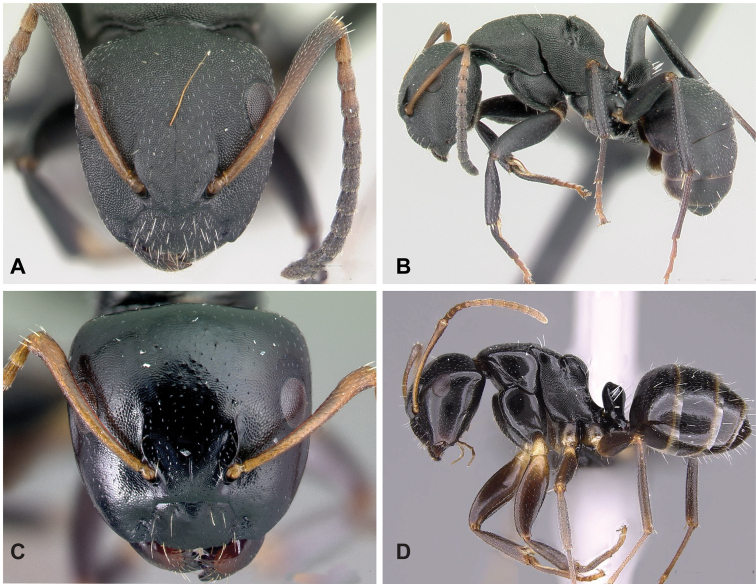
Morphological character of minor and major workers of *C.edmondi* species group **A** head of minor worker of *C.edmondi* (CASENT0178917) **B** body in lateral view of the same species **C** head of major worker of *C.varatra* (CASENT0217289) **D** body in lateral view of *C.zavo* (CASENT0060041).

**Minor worker**: in full-face view, head as long as broad, posterior margin broadly convex, lateral margins roughly straight (Fig. 6A).

**Major worker**: in full-face view, head subquadrate, posterior and lateral margins more or less straight to convex (Fig. 6C).

**Minor and major**: clypeus with broadly convex anterior margin and medially notched posterior margin. In profile, dorsal outline of mesosoma interrupted by metanotal suture, promesonotal suture distinct but not impressed. In profile, petiole always biconvex. Body integument opaque, densely reticulate-punctate (Fig. 6B–D). Few scattered hairs present on mesosomal dorsum, pubescence abundant to reduced.

**Remarks.** The *Camponotusedmondi* species group can be distinguished by the combination of the following characters: dorsolateral margin of propodeum marginate or extending into a sharp ridge, propodeal declivity usually concave, anterolateral corner of pronotum most often marginate, forecoxa larger than the width of mesopleuron, and propodeal dorsum abruptly sloping down to the insertion of the petiole.

### Definition of the *Camponotusefitra* species group

**Figure 7.**
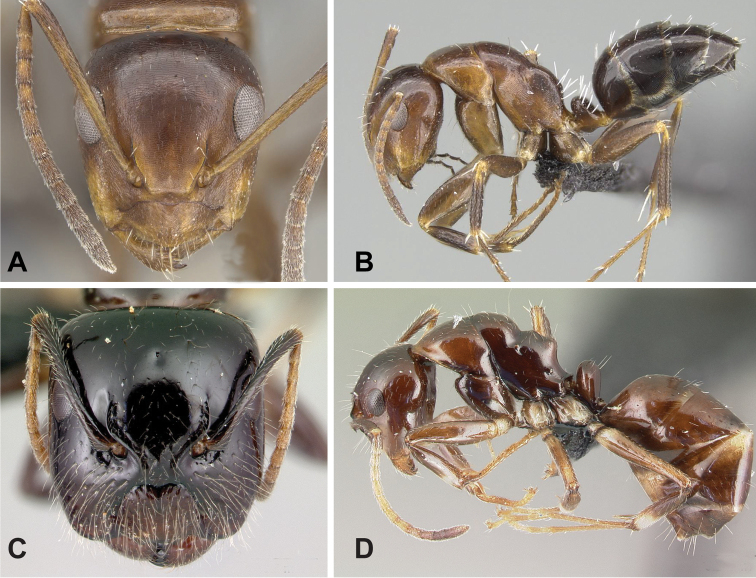
Morphological character of minor and major workers of *C.efitra* species group **A, B***Camponotusefitra* (CASENT0453926) **A** head of minor worker in full-face view **B** body in lateral view **C** head of major worker of *Camponotuschrislaini* (CASENT0499002) **D** minor worker of *Camponotuschrislaini* (CASENT0498999) in lateral view.

**Minor worker**: in full-face view, head subovate with strongly convex posterior and lateral sides. Anterior clypeal margin broadly rounded (Fig. 7A).

**Major worker**: in full-face view, head roughly quadrate or rectangular, head sides evenly convex (as in *C.efitra*) to straight (as in *C.chrislaini*) (Fig. 7C).

**Minor and major**: in profile, metanotal suture impressed, the propodeal dorsum forming a separate convexity from a domelike promesonotum, dorsum of propodeum below level of promesonotum. Clypeus evenly convex to straight in profile, its anterior margin convex to broadly triangular, not projecting, posterior margin weakly notched medially, major worker with massive mandible, occipital portion asetose, few standing setae present between frontal carinae but moderately present on clypeal dorsum and malar area. Petiole nodiform. Color brown. Integument sculpture finely imbricate to glabrous, apparently shiny (Fig. 7B, D).

**Remarks.** The *efitra* group is characterized by the color and sculpture of its integument; and a convex promesonotum separated from the propodeum by a weak metanotal suture. In Rakotonirina, Csősz & Fisher, 2017, *C.efitra* was considered a member of the *grandidieri* species group. Here, this species is separated from this group by the form of the mesosoma and the petiolar scale, the type of integument, and the general sculpture of major workers.

### Definition of the *Camponotusellioti* species group

**Figure 8.**
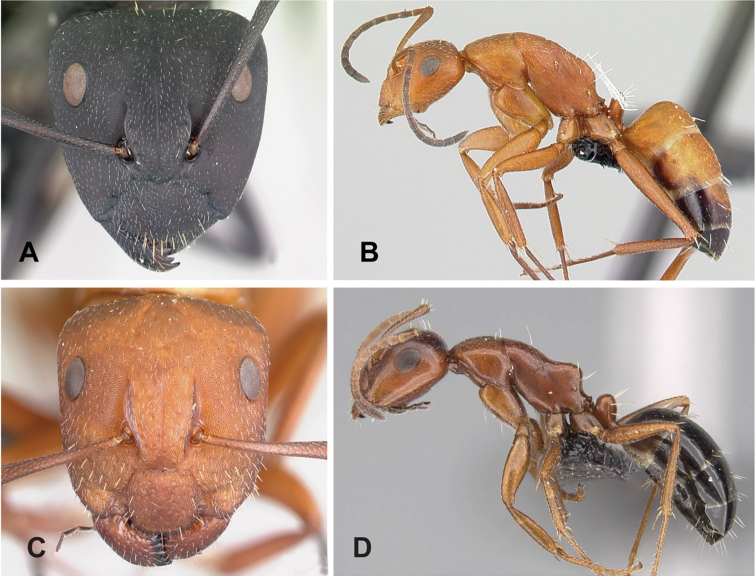
Morphological character of minor and major workers of *C.ellioti* species group **A** head of minor worker of *C.ellioti* (CASENT0450893) **B** minor worker of *C.maintikibo* in lateral view (CASENT0062631) **C** head of major worker of *C.maintikibo* (CASENT0062173) **D** minor worker of *C.andrianjaka* in lateral view (CASENT0243690).

**Minor worker**: in full-face view, head subtriangular, posterior margin evenly convex, head sides anterior to eyes subparallel and diverging posteriorly (Fig. 8A).

**Major worker**: in full-face view, head longer than broad, subtriangular to rectangular; with weakly convex sides (Fig. 8C).

**Minor and major**: anterior margin of clypeus of minor worker rounded and triangular, with anterolateral corner right-angled for *C.maintikibo*. Pronotum sometimes narrowly rounded or shouldered (*C.ellioti*, *C.maintikibo*) (Fig. 8B). In profile, dorsal outline of mesosoma evenly convex (*C.ellioti*, *C.maintikibo*) or slightly concave at the level of metanotal suture (*C.andrianjaka*) (Fig. 8D) or with impressed metanotal suture (*C.maintilany*). Humeral angles of pronotum well-marked. Petiole squamiform to nodiform. Integument finely imbricate to coarsely reticulate-foveolate. Pubescence long and distinct, few pairs of standing hairs on vertex.

**Remarks.** The *Camponotusellioti* species group is characterized by the following morphological features: mesosoma reddish, gaster dark brown to black; anterior clypeal margin projecting into broadly convex lobe; frontal carina short and diverging posteriorly, median carina absent; clypeus with short, rounded lobe; mandible subtriangular, apical tooth long; eyes large, elliptical, and break the lateral margin of head; in minor, head and occipital margin rounded, lateral head sides parallel before eyes.

*Camponotusellioti* has been considered a member of subgenus Myrmopiromis mainly because of the dense pilosity on its gastral dorsum and the humeral angle, but after morphological investigation, we find that the color of the integument can be a good indicator for separating Malagasy *Camponotus* species.

### Definition of the *Camponotusmadagascarensis* species group

**Figure 9.**
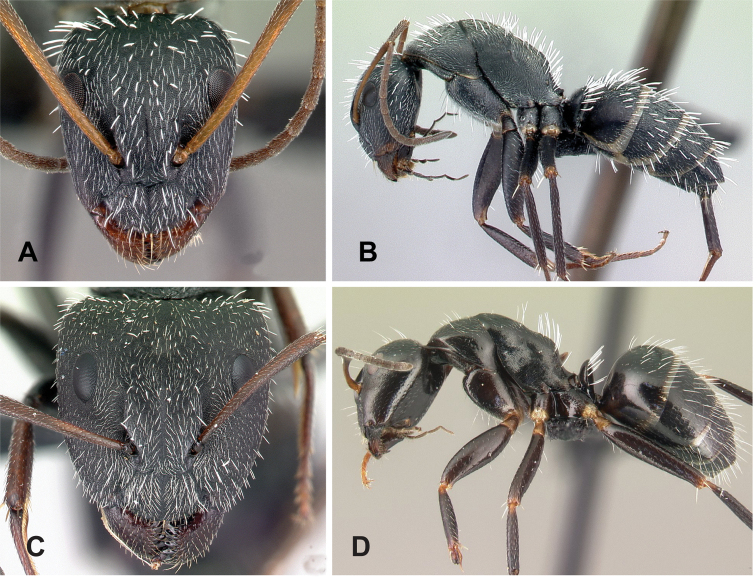
Morphological character of minor and major workers of *C.madagascarensis* species group **A, B** head in full-face view of minor worker of *C.madagascarensis* (CASENT0125551) **C** head of major worker of *C.voeltzkowii* (CASENT0217295) **D** minor worker of *C.ivadia* (CASENT0498634).

**Minor worker**: in full-face view, head elongate, lateral borders straight, feebly diverging posteriorly; posterior margin more or less convex (Fig. 9A).

**Major worker**: in full-face view, head rectangular, posterior and lateral borders slightly convex (Fig. 9D).

**Minor and major worker**: with the head in full-face view, anterior clypeal margin with an angular lobe, triangular or rectangular with rounded anterolateral angle; clypeus with median carina. In profile, dorsal line of mesosoma forms a continuous line, anterolateral corner of pronotum rounded. Petiolar node compressed anteroposteriorly and tapering dorsally; anterior face generally convex and posterior face straight. Posterior portion of the head, mesosomal dorsum, and the lateral margin of the propodeal declivity with scattered, whitish, erect hairs (Fig. 9B, D).

**Remarks.** The *Camponotusmadagascarensis* species group possesses the same characters as the *Camponotusniveosetosus* group, described in Rakotonirina et al. (2017); the name has been changed because these species are endemic to Madagascar. This group is morphologically well defined by the distinctive disposition of hairs on the gastral segment. Four species of this group, with the exception of *C.ivadia*, are historically recognized as members of subgenus Myrmopiromis. In this study, we conclude that *C.madagascarensis* are endemic to the Malagasy region, while *C.niveosetosus*, the most similar species, is from South Africa and possesses the following suite of different morphological characters: occipital margin almost straight and not distinctly convex so that the anterior margin of pronotum is distinctly roof-like in dorsal view, cuticle of the dorsum of head of major worker with distinctive sculpture pattern, mandible of major worker with seven teeth.

### Definition of the *Camponotusrepens* species group

**Figure 10.**
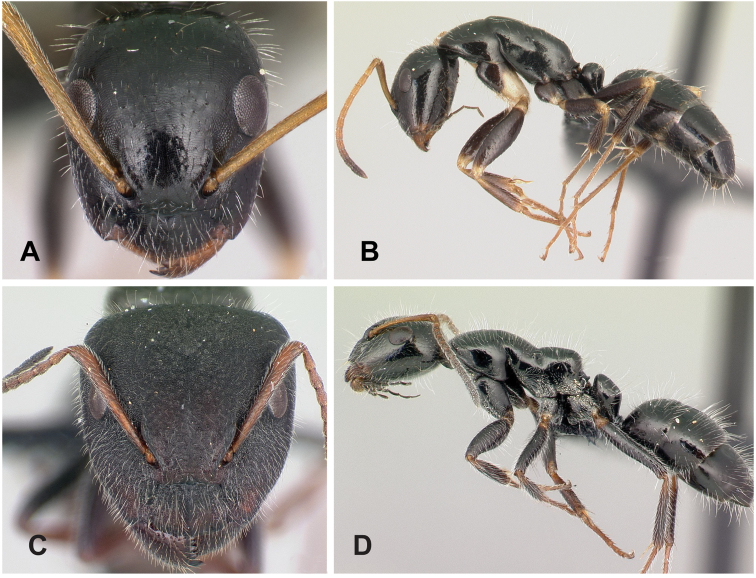
Morphological character of minor and major workers of *C.repens* species group **A, B** minor worker of *Camponotusrepens* (CASENT0217300), head in full-face view and body in lateral view **C** head of major worker of *C.jjacquia* (CASENT0217295) **D** minor worker of *C.jjacquia* (CASENT0445276).

**Minor worker**: in full-face view, head oval, with broadly convex posterior margin and lateral sides almost straight (Fig. 10A).

**Major worker**: in full-face view, head rectangular, posterior and lateral margins weakly convex (Fig. 10C).

**Minor and major**: with head in full-face view, clypeus carinate medially, its anterior margin forming a triangular lobe except for *C.repens*, which has a truncate anteromedian margin; dorsum of body covered with numerous slender, whitish, erect hairs and abundant, elongated pubescence. In lateral view, pronotum weakly convex with anterolateral portion marginate; promesonotal suture slightly depressed (Fig. 10B, D). Petiole nodiform except *C.claveri*. Head, mesosoma, and gaster black; legs generally much lighter in color than body. Integument shiny black.

**Remarks.** Workers of the *C.repens* group can be diagnosed by the integument being black and shiny in appearance, and the presence of erect white setae along the entire dorsum, all of which are about the same length and transversely arranged, except in *C.jjacquia*, which has filiform and relatively medium-sized setae. Median portion of clypeus with longitudinal carina; dorsum of mesosoma covered with numerous, slender, erect hairs of about the same length; in lateral view, petiolar node higher than long. Metanotal spiracle obvious, surrounded by shallow depression, not as in *C.christi* species group. The best method to discriminate between the *Camponotusrepens* species group and all other species stated in the Rakotonirina et al. (2018) *Camponotuschristi* species group, is to compare the anterodorsum of pronotal margin and the mesosomal pilosity. In the *C.repens* group, in dorsal view, the pronotal disc is rectangular and pilosity consists of thick, whitish, erect hairs. By contrast, in the *C.christi* group, in dorsal view, the pronotal disc is semicircular, and pilosity consists of slender, filiform hairs.

### Definition of the *Camponotusrobustus* species group

**Figure 11.**
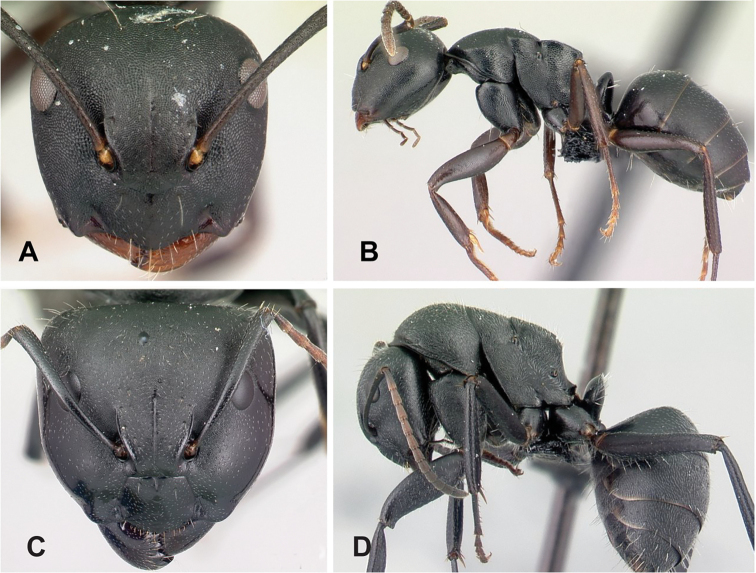
Morphological character of minor and major workers of *C.robustus* species group **A, B** minor worker of *C.zoro* (CASENT0049853) **A** head in full-face view **B** body in lateral view **C** head of major worker of *C.robustus* (CASENT0066724) **D** minor worker of *C.robustus* (CASENT0066723).

**Minor worker**: in full-face view, head longer than broad, lateral margins nearly straight and slightly diverging posteriorly, posterior margin broadly convex (Fig. 11A).

**Major worker**: in full-face view, head broader than long, lateral margin slightly convex (Fig. 11C).

**Minor and major worker**: clypeus not produced but with a rounded anterior margin. In profile, dorsal outline of mesosoma roughly straight and broken by metanotal suture (Fig. 11B, D); mesonotum and propodeum dorsum mostly rectangular and flattened in profile view. Ant black with opaque integument, pilosity suberect and white, pubescence short but distinct.

**Remarks.** The *Camponotusrobustus* species group can be distinguished by the combination of the following characters: in profile, posterodorsal face of petiole flat; anterodorsal margin of pronotum not arcuate, as in *C.edmondi* species group, but with broadly rounded to tuberculate humeral angle. In dorsal view, propodeum dorsum rectangular with distinct lateral margin; it forms an obtuse angle with the declivitous face. Clypeus with short anterior rounded lobe, sometimes notched medially (*C.ethicus*). Mesosomal dorsum complex, metanotal suture impressed. This group includes three species, two of which have been previously described as part of the *C.edmondi* species group. However, from Rakotonirina et al. (2016), this group can be recognized with the combination of the following characters: dorsolateral margin of propodeum marginate or extending into a sharp ridge, propodeal declivity usually concave, anterolateral corner of pronotum most often marginate, forecoxa larger than the width of mesopleuron, and usually propodeal dorsum abruptly sloping down to the insertion of the petiole. In addition, this species group has a prominent pronotal suture which is large and arcuate, making the characteristic form of mesonotum oval in dorsal view; propodeum dorsum mostly reduced in length and inclined towards petiole; and petiolar face distinctly convex, with at least one face, in dorsal view, resembling a handheld fan. *C.ethicus* and *C.robustus* are now placed with *Mayria* and form a monophyletic group with *Camponotuszoro* based on the morphological traits cited above.

#### ﻿Multivariate statistical analysis of morphometric data

**Figure 12.**
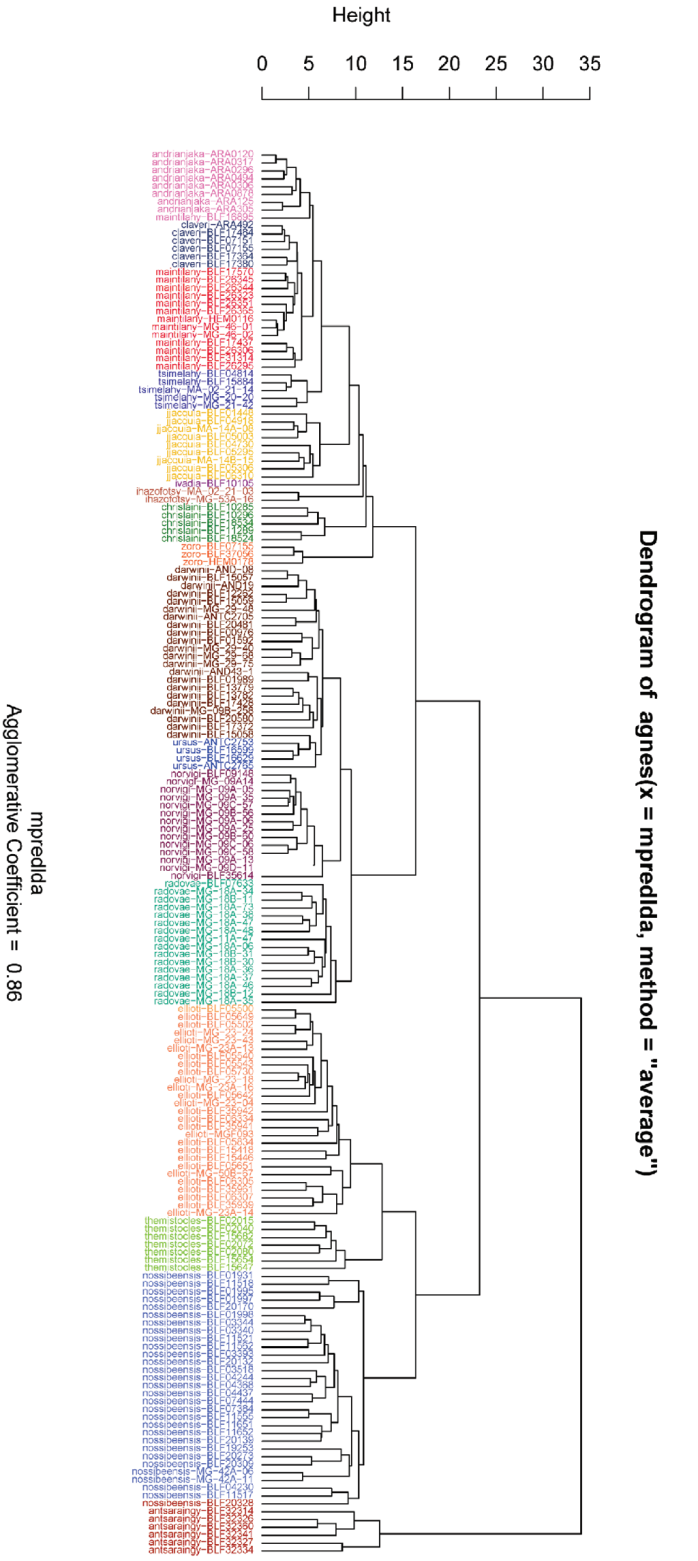
Dendrogram solution for the Malagasy Mayria subgenus based on morphometric data.

The NC Clustering dendrogram revealed 14 clusters (Fig. 12). Morphometric difference between *Camponotusclaveri* and *C.maintilany*, *C.darwinii* and *C.ursus* are not detected by the clustering methods. Moreover, the distribution of each species, along with some unmeasurable features such as density of hair, integument opacity, and color of hair/integument/appendages mentioned at the species description, allowed us to consider them as separate species.

#### ﻿Identification key to species for minor worker for Malagasy Camponotus (Mayria)

Some couplets related to the 22 species that have been transferred into the subgenus Mayria are taken from Rakotonirina et al. (2016, 2017, 2018).

**Table:** 

1	With the combination of the following characters: head broadened posteriorly, head width equal to or slightly longer than pronotal width; occipital margin straight to evenly convex; in dorsal view, pronotum subrectangular with subparallel margins; mesosoma short and high, forming a rounded V in dorsal view; in profile, propodeum dorsum flat to slightly convex, never concave; petiolar node ranging from nodiform to squamiform; body integument opaque and dull with noticeable sculpture; pilosity consists of thick setae (Fig. 2)	***CamponotusMayria* 2**
–	Without combination of above characters	**other *Camponotus* subgenera**
2	Hairs consist of enlarged setae with pointed ends, curving, mostly pale colored, uneven in length; bending anteriorly on mesosoma dorsum; sometimes forming a fur coating (Fig. 13A); head more quadrate, occipital margin straight to evenly convex; coronal line always present	**(*darwinii* species group) 3**
–	Hairs consist of straight white setae, filiform to straplike, never bending anteriorly on mesosoma dorsum (Fig. 13B); moderately abundant and all about the same length, widely spaced; head other than quadrate	**8**
3	Large and robust species (CS: 2.24±0.46, 1.92–2.70); in profile, basal face of propodeum half as long as declivitous face (Fig. 14A); density of hairs on the mesopropodeum dorsum more abundant than on the gastral segment, most conspicuous on pronotal dorsum; gastral segment distinctly reticulate-punctate (CL: 2.30±0.17, 2.00–2.78; ML: 3.28±0.20; 2.86–3.73	***nossibeensis***
–	Medium to smaller species; in profile, basal and declivitous faces of propodeum unequal in length (Fig. 14B)	**4**
4	Erect setae white, long, and sparse on occipital region and thorax, very dense on the gaster and concealing the surface; appressed pubescence long and silvery in appearance (Fig. 15A); in full-face view, head more quadrate (CL: 1.51±0.09, 1.35–1.69; CS: 1.45±0.24; 1.31–1.66; ML: 1.98±0.12, 1.79–2.20)	***norvigi***
–	Erect pilosity yellow or orange in color, appressed pubescence short and inconspicuous (Fig. 15B); in full-face view, head trapezoidal	**5**
5	Hairs densely distributed on mesosoma (Fig. 16A) or gastral dorsum (Fig. 16B)	**6**
–	Hairs sparsely distributed on mesosoma (Fig. 16C) or gastral dorsum (Fig. 6D)	**7**
6	Erect hairs on mesosoma consisting of orange, long, thick setae coarsely distributed on each portion, sutures well marked; short, sparse setae widely distributed on gaster; petiole with sharp edge; legs with brown femora, orange tibia, and testaceous basitarsus (Fig. 17A). (CS: 1.54±0.19, 1.43–1.64; CL: 1.58±0.06; 1.49–1.66; ML: 2.19±0.10; 2.01–2.31)	***ursus***
–	Erect hairs on mesosoma and gastral dorsum yellowish to pale ochreous, somewhat more abundant on the gaster and dense on the mesosoma; sutures obsolete; petiole with blunt edge; legs same color as mesosoma (Fig. 17B). (CS: 1.45±0.28; 1.22–1.63; CL: 1.49±0.11; 1.25–1.62; ML: 2.14±0.16; 1.80–2.51)	***darwinii***
7	Head elongate, occipital margin straight, lateral margins tapering to front; erect hairs on head, thorax, and gaster shorter and sparser; humeral angle more tuberculate; dorsum of propodeum longer than declivitous face and forms a right angle; ants entirely black (Fig. 18A, 18B) (CS: 1.82±0.37, 1.53–1.98; CL:1.90±0.13, 1.62–2.08; ML: 2.71±0.17, 2.34–2.91)	***themistocles***
-	Head ovoid, occipital and lateral margins convex; erect hairs on head, thorax, and gaster long and dense; humeral angle rounded; dorsum of propodeum and declivitous face about the same length and together form a rounded angle; mandibles and legs more reddish brown (Fig. 18C, 18D) (CS: 1.59 ± 0.22; 1.43–1.77; CL: 1.64±0.09, 1.49–1.81; ML: 2.31±0.14, 2.05–2.55)	***radovae***
8	Antennal scape entirely flattened longitudinally. In full-face view, lateral head sides straight and tapering to front (Fig. 19A); large in size (CS: 2.14±0.38; 2.00–2.33; CL: 2.30±0.14, 2.16–2.48; ML: 3.04±0.10, 2.92–3.18); decumbent spatulate setae present below the antennal insertion. Dorsum of mesosoma and petiole with long and thick hairs, gastral segment wholly pubescent with decumbent and suberect hairs long enough to overlap. (MPD/ML: 0.55±0.03, 0.52–0.60)	**(*antsaraingy* species group) *antsaraingy***
–	Antennal scape partially flattened. In full-face view, lateral head margin mostly convex; setae absent below the antennal insertion (Fig. 19B); medium sized. Hairs other than spatulate	**9**
9	Mesosoma partially submarginate or marginate (Fig. 20A)	**10**
–	Mesosoma completely immarginate, humeral angle may be tuberculate (Fig. 20B)	**25**
10	In profile, anterior margin of petiolar node convex and posterior margin either convex or roughly triangular; propodeal spiracle located on declivitous surface or at posterolateral margin of the propodeum; in dorsal view, mesonotum quite oval (Fig. 21A, 21B)	**(*edmondi* species group) 11**
–	In profile, anterior margin of petiolar node convex and posterior margin more or less straight; propodeal spiracle located on lateral portion of propodeum; in dorsal view, mesonotum dorsum more rectangular (Fig. 21C, 21D)	**20**
11	In profile, propodeum strongly compressed anteroposteriorly, without clear distinction between dorsal margin and declivity (Fig. 22A); in dorsal view, mesonotum broad, at least twice as broad as long (Fig. 22B)	**12**
–	In profile, propodeum not strongly compressed anteroposteriorly, propodeal dorsum and declivitous surface separated by blunt angle (Fig. 22C); in dorsal view, mesonotum narrow, less than twice as broad as long (Fig. 22D)	**13**
12	Posterodorsal corner of mesonotum raised into a bluntly rounded shield (Fig. 23A); somewhat larger species (CS: 1.235–2.667, 1.6; CL: 1.255–2.647, 1.625; ML: 1.843–2.922, 2.257)	***echinoploides***
-	Posterodorsal corner of mesonotum rounded, not forming an extended shield (Fig. 23B); somewhat smaller species (CS: 1–1.722, 1.265; CL: 0.961–1.725, 1.29; ML: 1.341–2.078, 1.623)	***galoko***
13	In profile, straight line connecting one end of dorsolateral carina of propodeum at the metanotal groove to the other end next to propodeal spiracle is conspicuously longer than posterolateral margin of propodeum (Fig. 24A)	***matsilo***
-	In profile, straight line connecting one end of dorsolateral carina of propodeum at the metanotal groove to the other end next to the propodeal spiracle is approximately as long as posterolateral margin of propodeum (Fig. 24B)	**14**
14	Dorsum of head and mesosoma densely and finely reticulate-punctate (Fig. 25A)	**15**
–	Dorsum of head and mesosoma smooth and shiny, superimposed by fine imbrication (Fig. 25B)	**18**
15	Dorsum of mesosoma with numerous erect hairs, pubescence conspicuous (Fig. 26A)	***mifaka***
-	Hairs lacking on dorsum of pronotum; a pair of hairs present on mesonotum; dorsum of propodeum covered with few erect hairs; hairs on propodeum mostly arise along the region separating dorsal surface and declivity; pubescence inconspicuous (Fig. 26B)	**16**
16	Distance between meso-metapleural suture and dorsolateral margin of propodeum remains the same along the dorsolateral carina of propodeum (Fig. 27A); no distinct angle between dorsal margin of propodeum and declivity, both portions apparently forming a straight line	***orombe***
-	Distance between meso-metapleural suture and dorsolateral margin of propodeum variable, largest near the junction of dorsolateral carina and declivitous surface (Fig. 27B); blunt angle or convexity between dorsal margin of propodeum and declivity distinct	**17**
17	With mesosoma in dorsal view, lateral margins of mesonotum roughly straight and gradually converging posteriorly; width of propodeum at the metanotal groove less than half the maximum width of mesonotum (Fig. 28A); with head in full-face view, anteromedian margin of clypeus truncate	***edmondi***
–	With mesosoma in dorsal view, lateral margins of mesonotum convex and strongly converging posteriorly; width of propodeum at metanotal groove greater than half the maximum width of mesonotum (Fig. 28B); with head in full-face view, anteromedian margin of clypeus triangular	***tafo***
18	In profile, mesonotal dorsum strongly sloping down to the level of propodeum, maximum length of mesonotum about as long as distance between metanotal groove and propodeal spiracle (Fig. 29A); in dorsal view, lateral margin of mesonotum not well defined and converging gradually towards metanotal groove (Fig. 29B); head and mesosoma brown	***tratra***
-	In profile, mesonotum slightly sloping down to the level of propodeum, maximum length distinctly shorter than distance between metanotal groove and propodeal spiracle (Fig. 29C); in dorsal view, lateral margin of mesonotum well defined and evenly convex, converging abruptly towards metanotal groove (Fig. 29D); head and mesosoma dark brown to black	**19**
19	In profile, anterodorsal corner of pronotum extending anteriorly into narrow edge but dorsolateral portion not marginate, junction of dorsum to lateral surface always rounded; blunt angle between dorsal margin of propodeum and declivity distinct, or the junction between both portions rounded (Fig. 30A). Antennal scape and gastral tergites I-III covered with abundant, appressed pubescence (Fig. 30B)	***zavo***
-	In profile, anterodorsal corner of pronotum extending anteriorly into narrow edge and dorsolateral portion marginate; junction of dorsum to lateral surface of pronotum sharply angulate; no distinct angle between dorsal margin of propodeum and declivity, both portions apparently forming a straight line (Fig. 30C). Antennal scape and gastral tergites I-III with scattered, appressed pubescence (Fig. 30D)	***varatra***
20	Appendages the same color as mesosoma. Body integument finely reticulate and matte; dorsal face of propodeum forms a right angle with the declivitous face (Fig. 31A)	**(*robustus* species group) 21**
–	Appendages lighter colored than mesosoma. Body integument finely imbricate, shining; dorsal face of propodeum forms a rounded angle with the declivitous face (Fig. 31)	**(*alamaina* species group) 23**
21	Level of the propodeal dorsum abruptly lower than level of the promesonotal dorsum; pronotal dorsum with few erect hairs; humeral angle extended anteriorly into a narrow ridge (Fig. 32A)	**22**
–	Level of propodeal dorsum not abruptly lower than level of promesonotal dorsum; pronotum covered with numerous erect hairs and pubescence; humeral angle slightly tuberculate, not extended into a narrow ridge (Fig. 32B)	***robustus***
22	Larger species. In dorsal view, mesonotum dorsum quadrate (Fig. 33A); in profile, petiole scale with acute summit, its anterior surface convex and posterior flat (CS: 1.92–2.58; ML: 3.49–4.18)	***ethicus***
-	Medium species. In dorsal view, mesonotum dorsum ovate (Fig. 33B); in profile, petiole scale with blunt summit, convex anterior and posterior surfaces; propodeum with blunt prominences (CS: 1.02±0.28, 0.92–1.14; CL: 1.02±0.11, 0.93–1.14; ML: 1.34±0.14, 1.22–1.50)	***zoro***
23	In profile, anterior margin of pronotum broadly rounding to the dorsum; dorsolateral and posterolateral margins of propodeum strongly carinate (Fig. 34A); somewhat larger species (CS: 0.991–2.222; CL: 1.078–2.275; ML: 1.62–2.902)	***alamaina***
-	In profile, anterior margin of pronotum very short and indistinct, the cervical shield apparently joins the pronotal dorsum directly; at least posterolateral margin of propodeum not strongly carinate, but simply marginate or rounded (Fig. 34B); generally smaller species (CS: 0.875–1.739; CL: 0.98–1.922;ML: 1.373–2.235)	**24**
24	In dorsal view, dorsolateral portion of propodeum with sharp carina, posterolateral margin marginate (Fig. 35A). In profile, width of mesopleuron, seen at the level of spiracle, about as large as that of lateral portion of propodeum; at least one pair of erect hairs present on propodeal dorsum	***androy***
-	In dorsal view, dorsal face of propodeum rounded to lateral face, junction without sharp carina, and posterolateral margin rounded (Fig. 35B). In profile mesopleuron, taken at spiracle level, much wider than lateral portion of propodeum; erect hairs lacking on propodeal dorsum	***bevohitra***
25	In full-face view, lateral sides of head rather straight and subparallel; anterior clypeal margin rounded (Fig. 36A). Ants bicolored, head and mesosoma relatively reddish, gaster black	**(*ellioti* species group) 26**
–	In full-face view, lateral sides of head convex; anterior clypeal margin projecting into a rectangular lobe (Fig. 36B). Ants entirely black	**29**
26	Body opaque, integument harder and sculptured. In profile, dorsal outline of mesosoma arcuate (Fig. 37A)	**27**
–	Body shining, integument thin and unsculptured. In profile, dorsal outline of mesosoma depressed at the level of metanotal suture (Fig. 37B)	**28**
27	Head, mesosoma, and petiolar node yellowish orange, gastral segments yellowish anteriorly and darkened posteriorly, very sparsely and finely pubescent; humeral angle rounded; petiole nodiform, node summit convex (Fig. 38A)	***maintikibo***
–	Head, mesosoma, and petiolar node reddish to dark brown, gastral segments all black with dense, appressed, golden pubescence; humeral angle tuberculate; petiole squamiform with sharp edge (Fig. 38B) (CS: 1.85±0.55, 1.45–2.42; CL:1.93±0.20, 1.57–2.43; ML: 2.74±0.22, 2.27–3.19)	***ellioti***
28	Petiole higher than long with sharp edge, dorsal surface of mesosoma with impressed metanotal suture which forms a transverse canal; gaster broadly rounded, oval; four pairs of standing hairs present on petiolar border (Fig. 39A) (CS: 0.84±0.05, 0.8––0.86; CL: 0.92±0.02, 0.89–0.95; ML: 1.32±0.03, 1.26–1.38)	***maintilany***
–	Petiole nodiform, node summit convex; dorsal surface of mesosoma with shallow metanotal groove; gaster elliptical; two pairs of standing hairs present on petiolar border (Fig. 39B) (CS: 0.84±0.07, 0.81–0.88; CL: 0.94±0.03, 0.91–0.99; ML: 1.33±0.03, 1.29–1.36)	***andrianjaka***
29	Ants brownish; promesonotal suture distinctly impressed (Fig. 40A)	**(*efitra* species group) 30**
–	Ants dark colored; promesonotal suture feebly impressed (Fig. 40B)	**31**
30	Base of scape without lobe; frontal carina divergent (Fig. 41A); head, mesosoma, and petiolar node brown to reddish brown, gastral segments uniformly dark brown to black; propodeum dorsum slightly convex (Fig. 41B); mandible with six teeth; legs same color as mesosoma	***efitra***
-	Base of scape with lobe; frontal carina sigmoid (Fig. 41C); head, mesopropodeum, and petiolar node brown, pronotum light brown, gaster dark brown; dorsum of propodeum distinctly concave (Fig. 41D); mandible with five teeth; mid- and forecoxa, apical portion of femora and basal portion of tibia yellowish (CS: 0.95±0.17, 0.87–1.02; CL: 1.02±0.06, 0.95–1.09; ML: 1.30±0.13, 1.13–1.43)	***chrislaini***
31	Whole body weakly sculptured, appears more or less shiny; median frontal carina absent (Fig. 42A)	**(*repens* species group) 32**
–	Whole body densely sculptured, appears subopaque or dull; median frontal carina impressed (Fig. 42B)	**(*madagascarensis* species group) 36**
32	Mesosomal profile continuous, forming a regular arch; the metanotal groove very shallow (Fig. 43A); petiolar node higher than long	**33**
–	Mesosomal profile discontinuous, not forming a regular arch; interrupted at the deep metanotal groove (Fig. 43B); petiole nodiform, node summit convex to straight	**34**
33	Forecoxae bicolored (Fig. 44A). In full-face view, head elongate, oval. Propodeum much rounded in lateral view. Dorsum of mesosoma covered with numerous slender, whitish, erect hairs, equal in length and arranged transversally; appressed pubescence prominent	***repens***
–	Forecoxae black colored (Fig. 44B). In full-face view, head more trapezoidal. Propodeum angulate in lateral view. Dorsum of mesosoma with few pairs of hairs; appressed pubescence very obscure (CS: 0.89±0.08, 0.84–0.93; CL: 0.97±0.04, 0.92–1.02; ML: 1.45±0.08, 1.35–1.53)	***claveri***
34	In profile, petiole longer than broad, anterior face leaning forward (Fig. 45A); propodeum acutely angular with a deep impression at the metanotal suture. Dorsal outline of mesosoma depressed at the level of metanotal suture; ants covered with fine, pointed, white hairs uneven in length; distributed sparsely on entire dorsum (CS: 0.99±0.14, 0.91–1.09; CL: 1.08±0.06, 0.99–1.18; ML: 1.67±0.08; 1.57–1.87)	***jjacquia***
-	In profile, petiole cubic or wider than long (Fig. 45B); anterior face straight and rounding gradually to its dorsum; angle of propodeum rounded, the metanotal suture only slightly or not at all impressed; scattered short hairs present on mesosoma dorsum	**35**
35	Head elongate (Fig. 46A); five to six pairs of standing hairs present on propodeum dorsum; petiole higher than long with convex edge (CS: 0.79±0.04, 0.77–0.81; CL: 0.92±0.02, 0.89–0.95; ML: 1.43±0.05, 1.34–1.46)	***tsimelahy***
–	Head more quadrate (Fig. 46B); one pair of stiff hairs on propodeal angle; petiole cubic with flat dorsum which forms a smooth curve with the posterior face (CS: 0.78±0.12, 0.75–0.81, CL: 0.83±0.06, 0.79–0.87; ML: 1.25±0.04, 1.22–1.27)	***ihazofotsy***
36	In profile, mesosoma long and low (MPH/ML: 0.35–0.47), propodeal dorsum longer than declivitous face (Fig. 47A); erect hairs lacking immediately behind lateral margin of clypeus	**37**
–	In profile, mesosoma short and high (MPH/ML: 0.45–0.71), propodeal dorsum shorter than declivitous face (Fig. 47B); a few scattered, erect hairs present immediately behind lateral margin of clypeus	**38**
37	In full-face view, whitish, erect hairs on posterior portion of head scattered laterally to level of anterior margin of eyes (Fig. 48A); eyes located at least in posterior fourth of head; in dorsal view, dorsal margin of petiolar node with two rows of erect hairs extending from upper half to apex; transverse pale strip on posterior margin of abdominal tergites large, width a fifth of the visible width of tergite (Fig. 48B)	***madagascarensis***
–	In full-face view, whitish erect hairs on posterior portion of head spread mostly to posterolateral angle of head, not reaching level of anterior margin of eyes (Fig. 48C); eyes located on posterior fifth of head; in dorsal view, dorsal margin of petiolar node with only one row of erect hairs extending from upper half to apex; transverse pale strip on posterior margin of abdominal tergites narrow, width an eighth of the visible width of tergite (Fig. 48D)	***descarpentriesi***
38	Basal half of antennal scape noticeably flattened dorsoventrally; head elongate; base of scape near basal condyle forming a lobelike extension (Fig. 49A); in full-face view, lateral head sides slightly convex (CWb/CL: 0.83, 0.81–0.84; CW/CL: 0.80, 0.78–0.82), dorsum of body with fewer scattered erect hairs and sparse pubescence; large species	***mita***
–	Basal half of antennal scape cylindrical, not strongly flattened (Fig. 49B); in full-face view, head subquadrate; base of scape near basal condyle forming a lobe	**39**
39	Body color matte black, entire body covered with numerous, thick, whitish hairs. In full-face view, occipital margin medially convex, anterior margin of clypeus triangular. In profile, dorsum of propodeum shorter than declivitous face (Fig. 50A) (CWb/CL: 0.91, 0.87–1.07; CW/CL: 0.89, 0.86–1.04)	***voeltzkowii***
–	Body color shiny black, white hair on dorsum thin, few in number. In full-face view, occipital margin straight medially, anterior margin of clypeus rectangular. In profile, dorsum of propodeum about the same length as declivitous face (Fig. 50B) (CS: 1.21±0.19; 1.13–1.3; CL: 1.28±0.08, 1.18–1.35; ML: 1.85±0.15, 1.69–2.04)	***ivadia***

**Figure 13.**
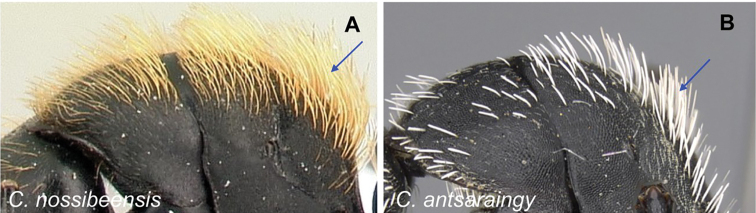
Type of hair on mesosoma dorsum **A***C.nossibeensis* (CASENT0191657) **B***C.antsaraingy* (CASENT0371032).

**Figure 14.**
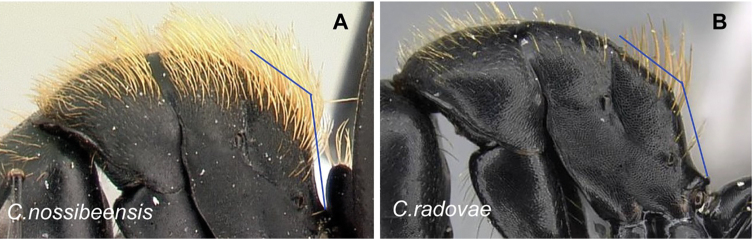
Density of hair on mesosoma dorsum and propodeum angle **A***C.nossibeensis* (CASENT0191657) **B***C.radovae* (CASENT0066208).

**Figure 15.**
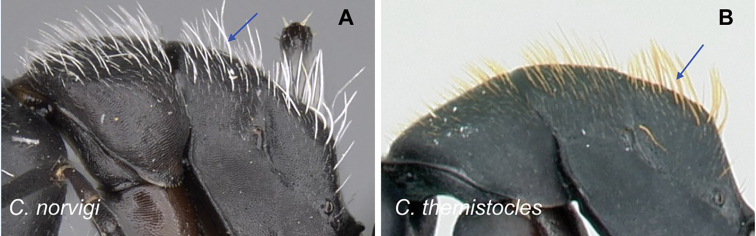
Hair color **A***C.norvigi* (CASENT0923254) **B***C.themistocles* (CASENT0217297).

**Figure 16.**
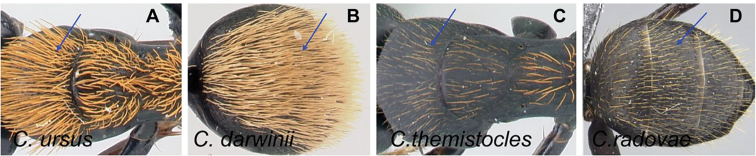
Mesosomal and gastral pilosity **A***C.ursus* (CASENT0217284) **B***C.darwinii* (CASENT0071420) **C***C.themistocles* (CASENT0217297) **D***C.radovae* (CASENT0121525).

**Figure 17.**
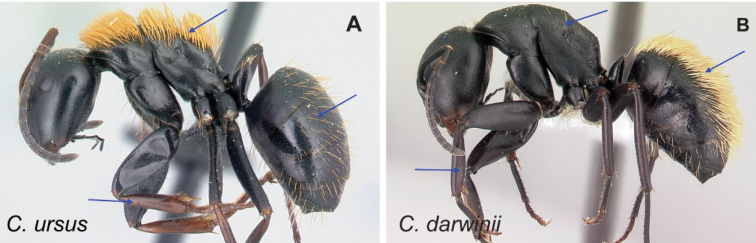
Dense hair on mesosoma dorsum **A***C.ursus* (CASENT0217284), and on gastral dorsum for **B***C.darwinii* (CASENT0179460).

**Figure 18.**
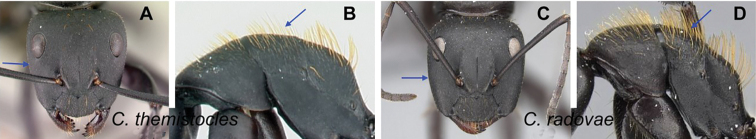
Head in full-face view **A, B***C.themistocles* (CASENT0217297) **C, D***C.radovae* (CASENT0066208) and mesosomal pilosity for both species.

**Figure 19.**
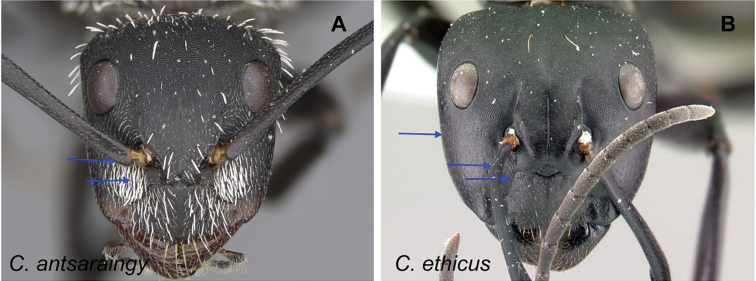
Head in full-face view **A***C.antsaraingy* (CASENT0371032). **B***C.ethicus* (CASENT0409948).

**Figure 20.**
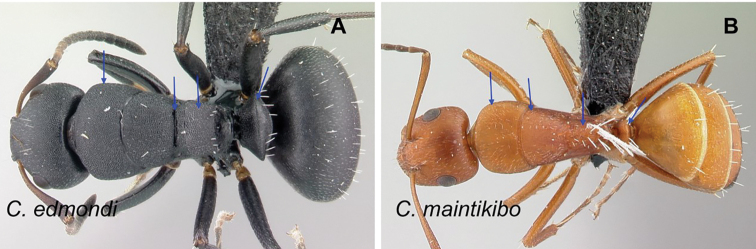
Body in dorsal view showing lateral margination of each part of mesosoma dorsum **A***C.edmondi* (CASENT0136511) **B***C.maintikibo* (CASENT0763877).

**Figure 21.**
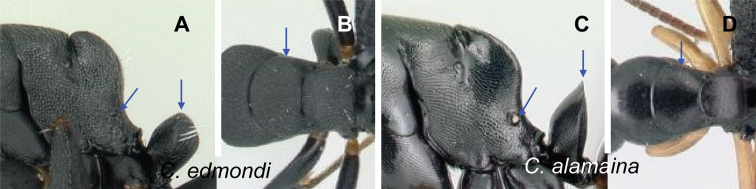
Mesosoma and petiole node in lateral view followed by mesonotum in dorsal view **A, B***C.edmondi* (CASENT0136511) **C, D***C.alamaina* (CASENT0481799).

**Figure 22.**
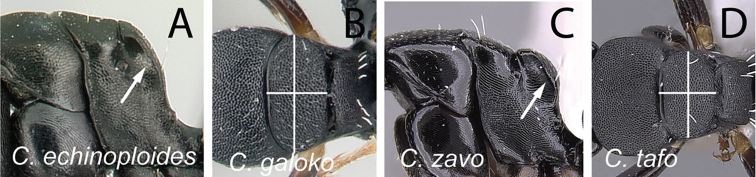
Mesosoma in lateral and dorsal views **A***C.echinoploides* (CASENT0409171) **B***C.galoko* (CASENT0178918) **C***C.zavo* (CASENT0060041) **D***C.tafo* (CASENT0763608).

**Figure 23.**
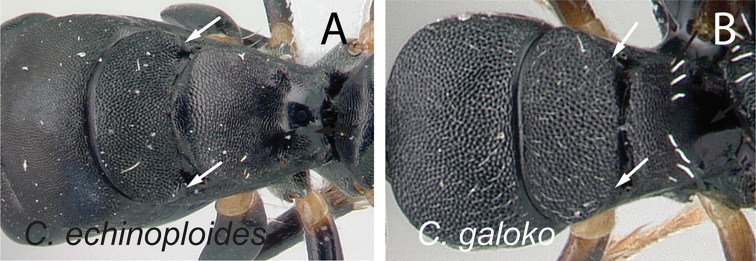
Mesosoma in dorsal view **A***C.echinoploides* (CASENT0409171) **B***C.galoko* (CASENT0178918).

**Figure 24.**
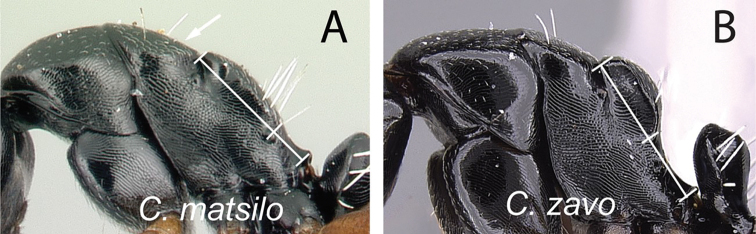
Mesosoma in lateral view **A***C.matsilo* (CASENT0121843) **B***C.zavo* (CASENT0060041).

**Figure 25.**
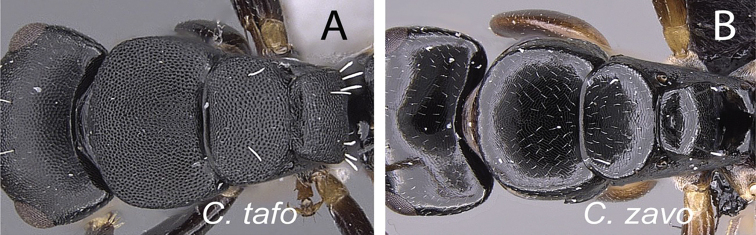
Head and mesosoma in dorsal view **A***C.tafo* (CASENT0763608) **B***C.zavo* (CASENT0060041).

**Figure 26.**
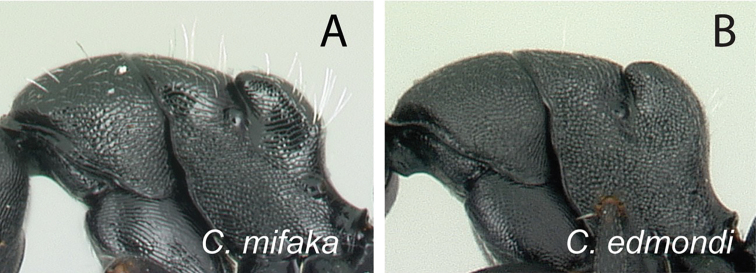
Mesosoma in lateral view **A***C.mifaka* (CASENT0217301) **B***C.edmondi* (CASENT0136511).

**Figure 27.**
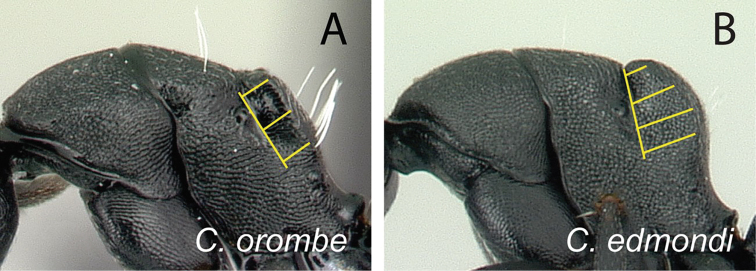
Mesosoma in lateral view **A***C.orombe* (CASENT0178923) **B***C.edmondi* (CASENT0136511).

**Figure 28.**
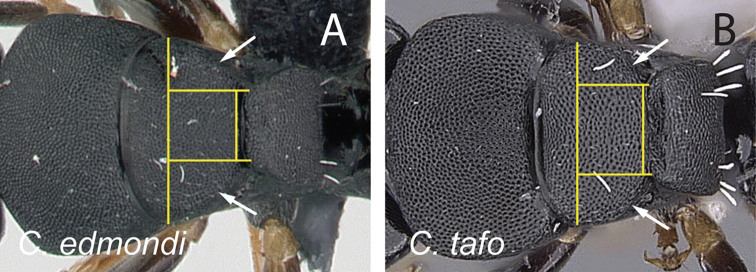
Mesosoma in dorsal view **A***C.edmondi* (CASENT0134980) **B***C.tafo* (CASENT0763608).

**Figure 29.**
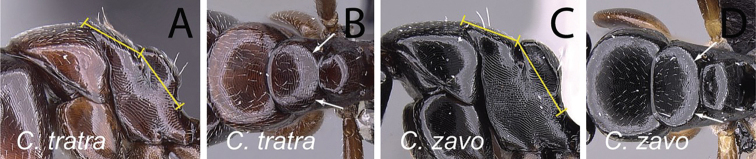
Mesosoma in lateral view and in dorsal view **A, B***C.tratra* (CASENT0763608) **C, D***C.zavo* (CASENT0060041).

**Figure 30.**
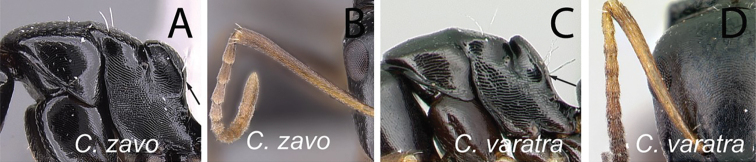
Mesosoma in lateral view and antennal scape in full-face view **A, B***C.zavo* (CASENT0060041) **C***C.varatra* (CASENT0492888) **D***C.varatra* (CASENT0409723).

**Figure 31.**
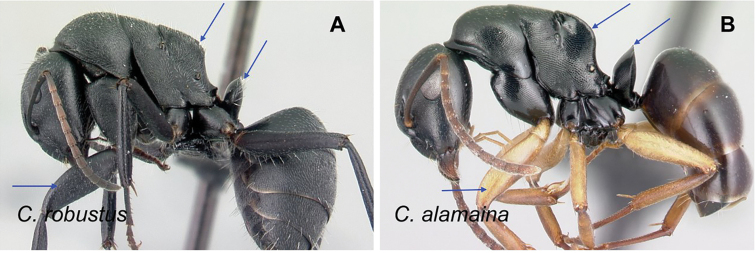
Body in lateral view **A***C.robustus* (CASENT0066723) **B***C.alamaina* (CASENT0499291).

**Figure 32.**
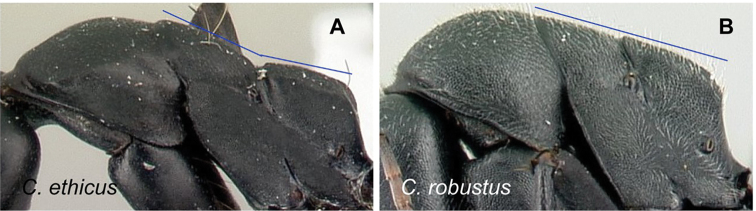
Mesosoma in lateral view **A***C.ethicus* (CASENT0409949) **B***C.robustus* (CASENT0066723).

**Figure 33.**
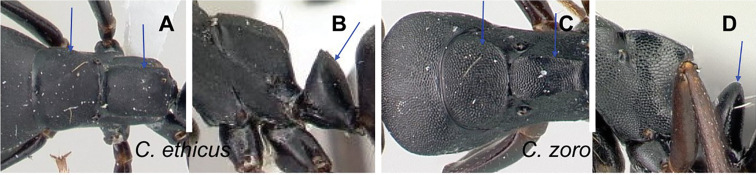
Mesonotum and propodeum in dorsal view, petiole in lateral view **A, B***C.ethicus* (CASENT0409949) **C, D***C.zoro* (CASENT0049853).

**Figure 34.**
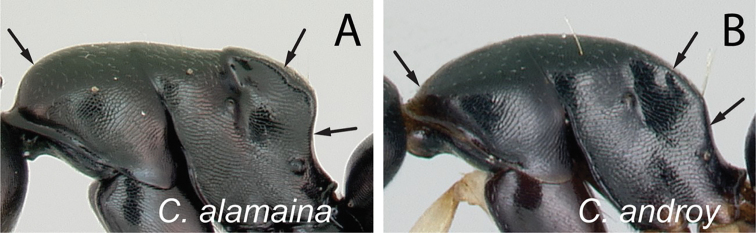
Mesosoma in lateral view **A***C.alamaina* (CASENT0499291) **B***C.androy* (CASENT0453723).

**Figure 35.**
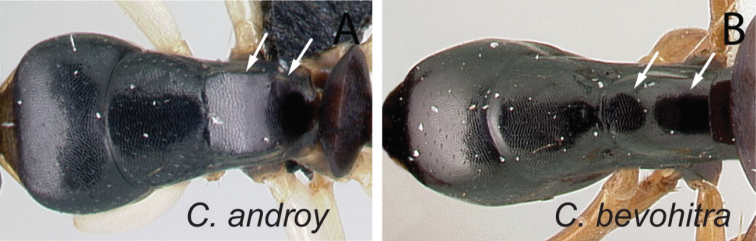
Mesosoma in dorsal view **A***C.androy* (CASENT0453723) **B***C.bevohitra* (CASENT0437238).

**Figure 36.**
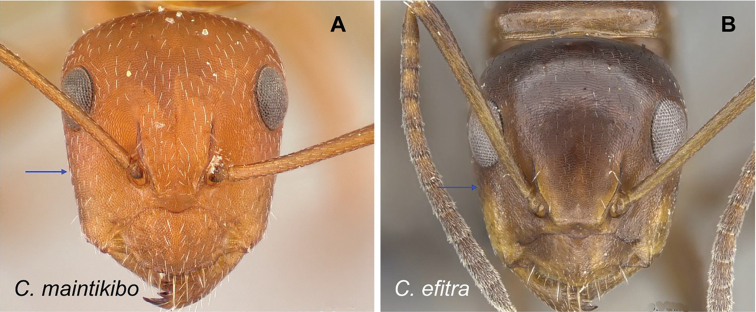
Head in full-face view **A***C.maintikibo* (CASENT0763877) **B***C.efitra* (CASENT0453926).

**Figure 37.**
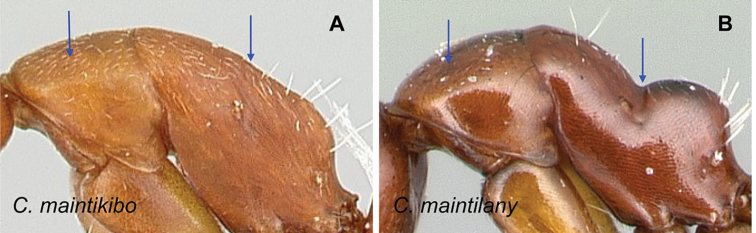
Mesosoma in lateral view **A***C.maintikibo* (CASENT0763877) **B***C.maintilany* (CASENT0120678).

**Figure 38.**
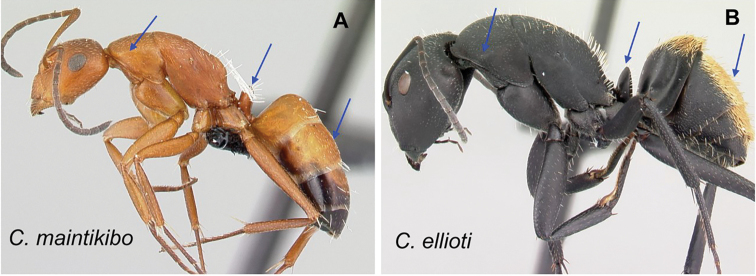
Body in lateral view **A***C.maintikibo* (CASENT0763877) **B***C.ellioti* (CASENT0450893).

**Figure 39.**
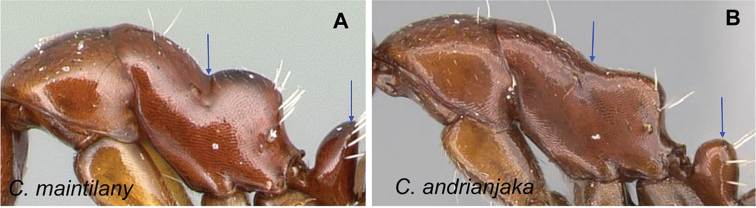
Mesosoma in lateral view **A***C.maintilany* (CASENT0120678) **B***C.andrianjaka* (CASENT0243690).

**Figure 40.**
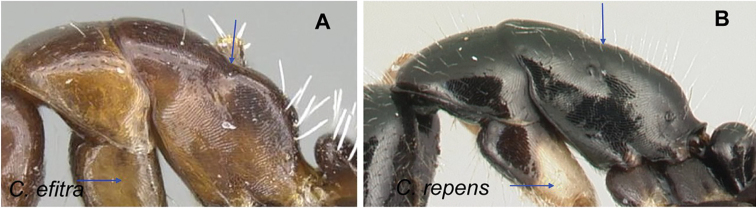
Mesosoma in lateral view **A***C.efitra* (CASENT0453926) **B***C.repens* (CASENT0481832).

**Figure 41.**
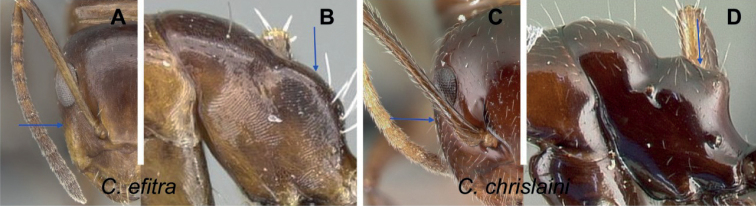
Antennal scape and mesopropodeum in lateral view **A, B***C.efitra* (CASENT0453926) **C,D***C.chrislaini* (CASENT0498999).

**Figure 42.**
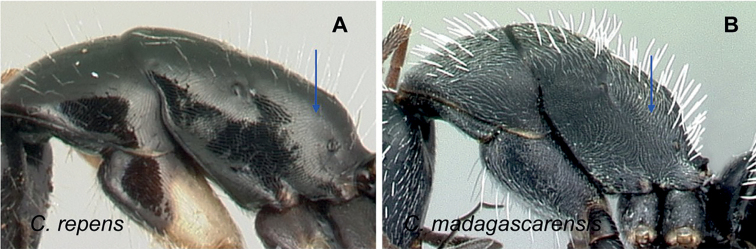
Mesosoma in lateral view **A***C.repens* (CASENT0481832) **B***C.madagascarensis* (CASENT0125551).

**Figure 43.**
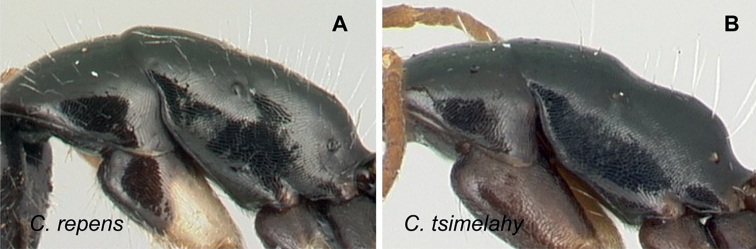
Mesosoma in lateral view **A***C.repens* (CASENT0481832) **B***C.tsimelahy* (CASENT0446651).

**Figure 44.**
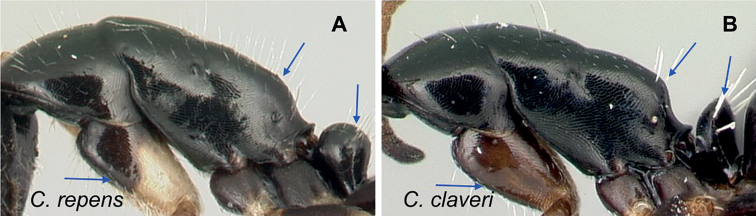
Mesosoma in lateral view **A***C.repens* (CASENT0481832) **B***C.claveri* (CASENT0490618).

**Figure 45.**
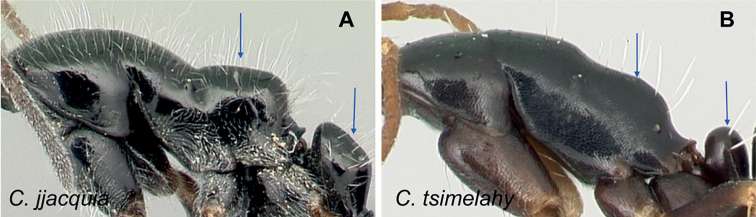
Mesosoma in lateral view **A**C.*jjacquia* (CASENT0445276) **B***C.tsimelahy* (CASENT0446651).

**Figure 46.**
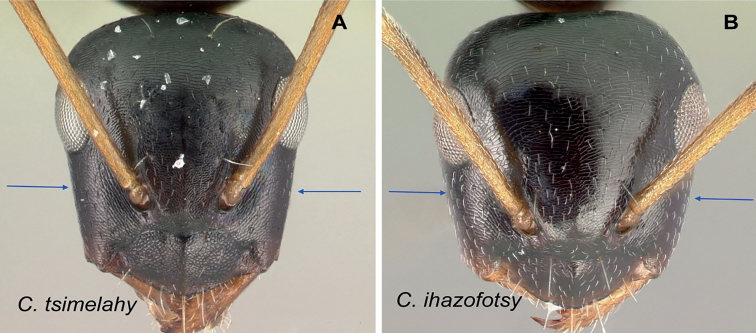
Head in full-face view **A***C.tsimelahy* (CASENT0446651) **B***C.ihazofotsy* (CASENT0062675).

**Figure 47.**
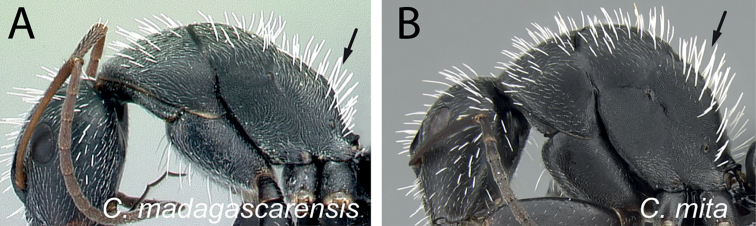
Head and mesosoma in lateral view **A***C.madagascarensis* (CASENT0125551) **B***C.mita* (CASENT0498906).

**Figure 48.**
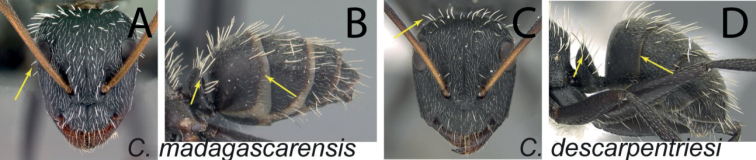
Head in full-face view, petiole and gastral segments in lateral view **A, B***C.madagascarensis* (CASENT0125551, CASENT0101383) **C, D***C.descarpentriesi* (CASENT0763876).

**Figure 49.**
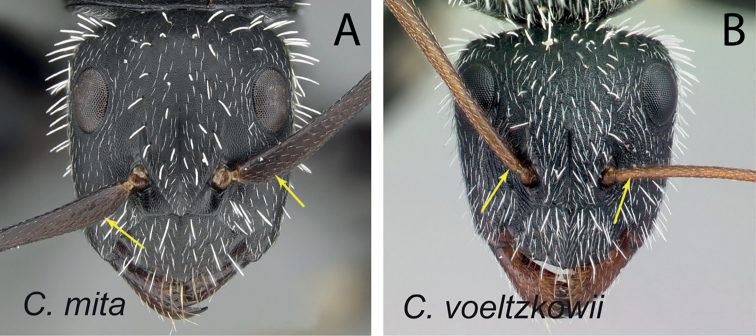
Head in full-face view **A***C.mita* (CASENT0498906) **B***C.voeltzkowii* (CASENT0121619).

**Figure 50.**
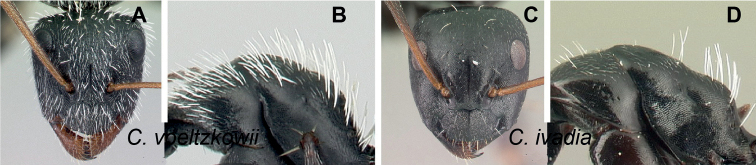
Head in full-face view and mesosoma in lateral view **A, B***C.voeltzkowii* (CASENT0121619) **C, D***C.ivadia* (CASENT0498634).

## ﻿Species accounts

### The *Camponotusalamaina* species group

Descriptions of the three species are illustrated in Rakotonirina et al. (2016): *Camponotusalamaina* Rakotonirina, Csősz & Fisher, 2016 on pp. 105–110, *Camponotusandroy* Rakotonirina, Csősz & Fisher, 2016 on pp. 110–113, and *Camponotusbevohitra* Rakotonirina, Csősz & Fisher, 2016 on pp. 113–115.

### The *Camponotusantsaraingy* species group

#### ﻿ Camponotus antsaraingy sp. nov.

Taxon classificationAnimaliaHymenopteraFormicidae

E5B75706-343E-523F-BFA2-FCE6EDDE3A5B

http://zoobank.org/CE9B7FC8-9992-4E11-AB74-B4B69A1725C5

**Figure 51.**
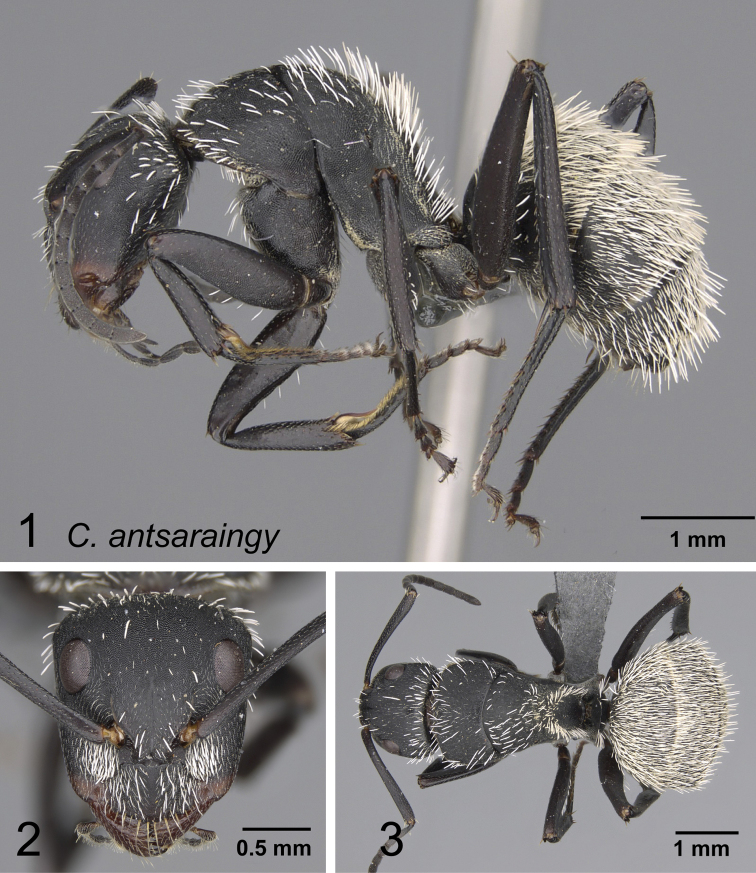
*Camponotusantsaraingy* minor worker (CASENT0371032) **1** body in lateral view **2** head in full-face view **3** body in dorsal view.

Figures 4 A–D , 13B , 19A , 51

##### Holotype worker.

Madagascar, Province Antsiranana, Antsaraingy, –12.90362, 49.65921, 66 m, littoral forest, ground nest, 21 October 2013 (BLF), collection code: BLF32314, specimen code: CASENT0371032 (CAS). **Paratypes.** Two workers with same data as holotype but collection code: BLF32341, specimen code: CASENT0371026 and CASENT0840640 (CAS).

##### Worker diagnosis.

Integument entirely black, coarsely sculptured; gaster densely covered with enlarged white hairs; anterior clypeal margin with a rectangular lobe, broadly rounded medially; antennal scape flattened longitudinally; appressed, pointed-oval, whitish hairs present next to the antennal insertion; dorsal face of propodeum distinctly shorter than declivitous face; only three first gastral tergites visible dorsally.

##### Description of minor worker.

Large-sized species. Absolute cephalic size (CS: 2.14±0.38, 2.00–2.33). In full-face view, head elongate (CWb/CL: 0.34±0.03; 0.32–0.35); posterior margin of head straight, lateral margin of head even straighter, feebly converging toward base of mandible. Eyes elliptical, sublateral, and placed next to the vertex (PoOC/CL: 0.08±0.02; 0.07–0.09). Mandibles triangular with six teeth. Clypeus strongly carinate throughout its length, with produced, angular, anterior margin, lateral corner angulate and median portion a rounded triangle (ClyL/GPD: 0.25±0.05, 0.22–0.28). Antennal scape longitudinally flattened, its base scarcely enlarged (SL/CS: 0.44±0.04, 0.42–0.45). In lateral view, mesosoma short and high, its dorsum smoothly arcuate, metanotal suture obsolete. Humeral angle rounded. Dorsal and declivitous face of propodeum difficult to distinguish and forming a rounded angle (MW/ML: 0.27±0.02; 0.26–0.28; MPH/ML: 0.21±0.02, 0.20–0.22). In lateral view, petiole squamiform with sharp edge, anterior face short and converging to the highest point of the petiole, posterior face straight. Ventral face of femora marginate laterally, the outer margin of tibia with long, whitish setae. Head and mesosoma coarsely reticulate-punctate, legs and gastral tergite finely imbricate. Mandible finely foveolate with sparse punctures. Whitish, suberect hairs present on occipital portion, to the anterior margin of eyes, and on posteroventral head surface, scarcely distributed on mesosoma dorsum but much closer on declivity; decumbent, spatulate, whitish hair on the ventral section of gena, below the antennal socket, on the posterolateral margin of clypeus, promesonotum dorsum, petiolar node, and gastral tergite; suberect and decumbent whitish hairs present on gastral segment. Entire body blackish except the mandible testaceous to reddish.

##### Description of major worker.

With characteristics of minor workers, except: head more cordate (CS: 3.32±0.21, 2.94–3.55) (CWb/CL: 0.96±0.01, 0.95–0.99); lateral margins slightly convex and tapering anteriorly. Eyes elliptical, smaller compared to head size (EL/CS: 0.20±0.01; 0.20–0.21), placed dorsally next to posterior margin of frontal carina. Antennal scape short and not exceeding posterior margin of head (SL/CS: 0.81±0.04; 0.76, 0.88); mandible more robust. Mesosoma and pilosity same as minor worker.

##### Distribution and biology.

This new species is only known from Antsaraingy, a littoral forest, from 66 to 90 m in elevation, located in the northern portion of Madagascar (Fig. 70A). Nests were found underground and inside termite mounds; four colonies were collected to represent this species.

##### Discussion.

Despite its large size, *Camponotusantsaraingy* may be confused with *Camponotusmita* and *Camponotusvoeltzkowii* because in these three species the propodeal face of the propodeum is more reduced than the declivitous face. In *C.antsaraingy* the antennal segments are entirely flattened while in *C.mita* only the basal half is flattened; *C.voeltzkowii* has circular antennal segments. The pubescence on the gastral segment consists of thick, decumbent hair of the same type as the pilosity in *C.antsaraingy*, with short and filiform hairs on the other two species.

##### Etymology.

The species epithet is in reference to the type locality.

##### Additional material examined.

**Province Antsiranana**: Antsaraingy, –12.90665, 49.6606, 90 m, littoral forest, BLF (CAS).

### The *Camponotusdarwinii* species group

#### ﻿ Camponotus darwinii

Taxon classificationAnimaliaHymenopteraFormicidae

Forel

1EDEEF99-9D99-54D8-8F8F-EDDBEF505189

**Figure 52.**
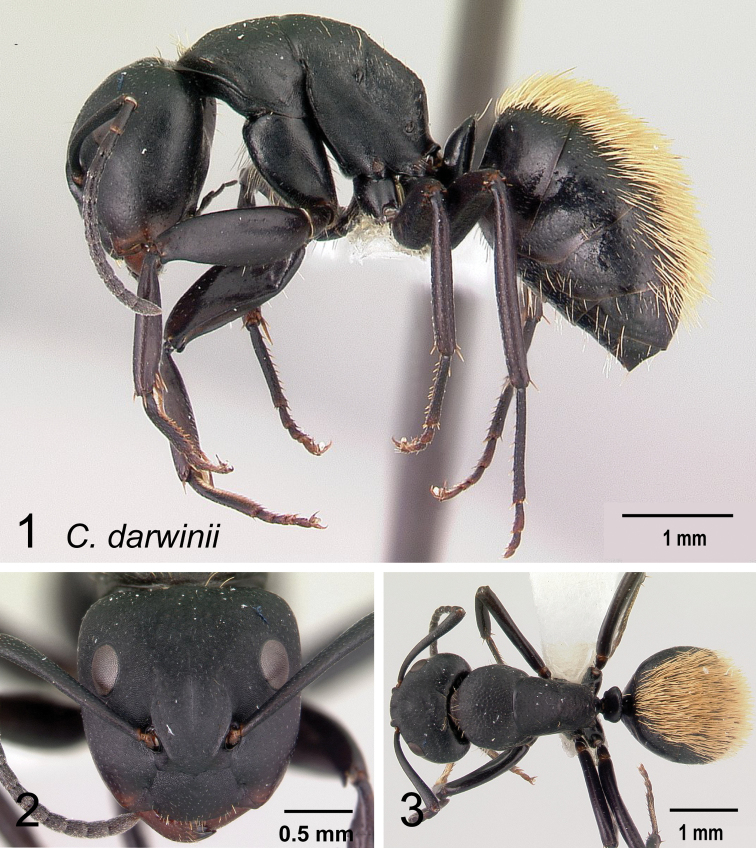
*Camponotusdarwinii* minor worker (CASENT0179460) **1** body in lateral view **2** head in full-face view **3** body in dorsal view.

Figures 5A , 16B , 17B , 52

Camponotus darwinii Forel, 1886: 179. Lectotype minor worker, present designation, Centre de Madagascar [Ambatomanjaka, Miarinarivo, –18.766947, 46.869107, 1343 m] (Hildebrandt), AntWeb CASENT0101391 (MNHG). Paralectotypes: one alate queen and five major workers, same data as lectotype, but specimen coded respectively as CASENT0101385 (queen) andCASENT0101386 (MNHG), CASENT0104426, CASENT0104427, FOCOL2468 and FOCOL2469 (ZMHB) (examined).
Camponotus (Myrmobrachys) darwinii Forel, 1914: 270; in Camponotus (Myrmepomis): Emery, 1920: 258; in Camponotus (Myrmopiromis): Wheeler, 1922: 1051; Emery, 1925: 128; Bolton, 1995: 95, 131.
Camponotus darwinii var. rubropilosus Forel, 1891: 44. Lectotype major worker, present designation, Madagascar, Antananarivo (Camboué), AntWeb CASENT0101392 (MHNG). Paralectotypes: three major workers same data as the lectotype CASENT0101393 (MHNG), CASENT0104628 (ZMHB), CASENT0101193 (NHMB).
Camponotus (Myrmopiromis) darwinii var. rubropilosus Wheeler, 1922: 1051; Emery, 1925: 128; Bolton, 1995: 121. Syn. nov.
Camponotus darwinii r. rubropilosus var. robustior Forel, 1905: 165. Lectotype minor worker, present designation, Toliara, Menabe, Beronono; Mahabo, –21.0, 45.0, 339 m (Woltzkow), AntWeb CASENT0104629 (MNHG). Paralectotypes: two major workers, same data as lectotype, but specimen coded respectively as CASENT0101195andCASENT0101380 (MHNG).
Camponotus (Myrmopiromis) darwinii r. rubropilosus var. robustior Wheeler, 1922: 1051; Emery, 1925: 128; Bolton, 1995: 121. Syn. nov.

##### Worker diagnosis.

Body integument matte black, finely reticulate-punctate; mesosoma and/or gastral dorsum with dense, long, and yellowish pilosity and pubescence; anterior clypeal margin with short, rounded, rectangular lobe; petiole squamiform.

##### Description of minor worker.

Medium size. Absolute cephalic size (CS: 1.45±0.28; 1.22–1.63). In full-face view, head more quadrate (CWb/CL: 0.37±0.03, 0.35–0.41); posterior margin of head almost flat, lateral margin of head slightly convex and tapering to front. Eyes circular, placed midway from the occipital corner (PoOC/CL: 0.08±0.02; 0.06–0.09). Mandibles triangular with six teeth. With head in full-face view, anterior clypeal margin with a short, rounded, rectangular lobe; less convex in lateral view, not medially marginate (ClyL/GPD: 0.25±0.04, 0.23–0.28). Antennal scape circular and long, surpassing the occiput by the length of two basal funiculi (SL/CS: 0.40±0.04; 0.37–0.43). In lateral view, anterodorsal corner of pronotum rounded, mesosomal dorsal outline feebly and rather evenly arcuate above, dorsal face of propodeum generally longer than declivitous face (MW/ML: 0.21±0.02; 0.19–0.23; MPD/ML: 0.24±0.03; 0.20–0.26). In lateral view, petiolar node cuneate, anterodosum straight basally and inclined dorsally to reach the flattened posterodorsal margin. Tibia tubular, not prismatic, its extensor profile with suberect setae. Head dorsum is matte, finely reticulate-punctate with sparse punctures on malar area; mesosoma striate in lateral view; in dorsal view, petiole, mesosoma, and appendages less shiny, finely reticulate-striolate except the abdominal sternite, which is more matte. Mandible finely reticulate-punctate with sparse oblique punctures. Hairs yellowish white, orange, or tawny colored, erect hairs usually abundant, at least on gastral tergite, often on vertex, occipital portion, and thorax dorsum; petiolar node with 4 pairs of hairs on its dorsum. Shiny black, with mandible and basitarsus reddish to brownish.

##### Description of major worker.

Characteristics of minor workers, except: head much wider posteriorly (CS: 2.14±0.26, 1.70–2.47; CWb/CL: 1.04±0.06, 0.94–1.20); lateral margins convex and converging towards base of mandible. Eyes semicircular, placed dorsolaterally midway from the occipital margin (PoOC/CL: 0.22±0.01, 0.19–0.24). Clypeus much more quadrate in full-face view (ClyL/GPD: 0.72± 0.09, 0.48–0.81), its anterior margin slightly concave medially. Antennal scape short, just surpassing the occipital border (SL/CS: 0.83±0.08, 0.75–0.99). Dorsal outline of mesosoma forms a continuous line so that the basal portion of propodeum forms an obtuse angle with the declivity. Lateral portion of head finely reticulate above, punctate to finely areolate below.

##### Distribution and biology.

*Camponotusdarwinii* is a famous species which is distributed widely across central of Madagascar, with a range extending from Ankazobe to Mount Papango, PN Befotaka Midongy (Fig. 58A). It has been collected from a wide variety of different habitats, including rainforest, montane rainforest, Uapaca forest, and Uapaca woodland as well as disturbed habitats such as urban and garden areas and cultivated land (Tavy). The species has also been found nesting inside *Meliaazedarach* and *Phellolophiummadagascariense*. Habitat elevations ranged from 1069–2150 m.

##### Discussion.

*Camponotusdarwinii* should be separable from most similar species in this group by the combination of the following characters: presence of dense hairs on mesosoma and/or gastral dorsum, mandible and malar area distinctly reddish in color, propodeal and declivitous faces unequal in length. This species is much closer to *C.ursus* but differs in the density of mesosomal hairs and the aspect of the petiole. In addition, *C.darwinii* is much more robust than *C.ursus.*

##### Additional material examined.

**Province Antananarivo**: Analamanga Region, District of Ankazobe, Ambohitantely, 46 km NE of Ankazobe, –18.198, 47.2815, 701 m, Forêt sclerophylle (MG) (CAS); Ambatolaona, –18.928, 47.88283, 1382 m, urban/garden (BLF) (CAS); Kaloy, –18.58998, 47.65102, 1423 m, disturbed montane rainforest, (BLF) (CAS); Ilafy, –18.85415, 47.56575, 1385 m, urban/garden (BLF) (CAS); Forêt de galerie, Telomirahavavy, 23.4 km NNE Ankazobe, –18.12167, 47.20627, 1520 m, disturbed gallery montane forest (BLF) (CAS); Stn. Forestiѐre Manjakatompo, –19.35, 47.31667, 2000 m, montane rainforest (Mark Pigeon) (CAS); 25 km NNE Ankazobe, –18.1, 47.18333, 1500 m, rainforest (PSW) (PSWC); Stn. Forestiѐre Manjakatompo, –19.35, 47.31667, 1600 m, montane rainforest (PSW) (PSWC); –18.91667, 47.53333, 1350 m, park/garden (PSW) (PSWC); Parc de Tsimbazaza; Antananarivo renivohitra, –18.93, 47.52611, 1275 m (D.M. Olson) (PSWC); –18.91667, 47.53333, 1300 m, park/garden (PSW) (PSWC); 3 km 41° NE Andranomay, 11.5 km 147° SSE Anjozorobe, –18.47333, 47.96, 1300 m, montane rainforest (FGAT) (CAS); Parc Botanique et Zoologique de Tsimbazaza, –18.93233, 47.91167, 1300 m (D.H. Kavanaugh) (CAS); Angavokely, –18.93333, 47.75 (B. Pettersson) (PSWC).

**Province Fianarantsoa**: 29 km SSW Ambositra, Ankazomivady, –20.77667, 47.165, 1700 m, disturbed montane rainforest (B.L. Fisher) (CAS); Forêt d’ Atsirakambiaty, 7.6 km 285° WNW Itremo, –20.59333, 46.56333, 1550 m, montane rainforest (FGAT)(CAS); 2 km W Andrambovato, along river Tatamaly, –21.51167, 47.41, 1075 m, cultivated land (tavy), recent 1–2 year-old tavy (BLF) (CAS); stream area, 900 m E of Isalo National Park Interpretive Center, –22.62667, 45.35817, 750 m, open area near stream (R. Harin’Hala) (CAS); Parc National d’Andringitra, Plateau d’Andohariana, 39.8 km 204° Ambalavao, –22.18767, 46.90083, 2150 m, rubicole thicket at base of cliff (BLF) (CAS); Vohiparara, –21.23333, 47.36667 (A. Pauly) (CAS); Miandritsara Forest, 40 km S of Ambositra, –20.79267, 47.17567, 822 m, low altitude rainforest (MG) (CAS); dry wash, 1 km E of Isalo National Park Interpretive Center, –22.62667, 45.35817, 885 m, dry wash (R. Harin’Hala) (CAS); Parc National Befotaka-Midongy, Papango 28.5 km S Midongy-Sud, Mount Papango, –23.84083, 46.9575, 1250 m, montane rainforest (BLF) (CAS); Parc naturel communautaire, 29.3 km SW Ambositra, –20.79751, 47.1791, 1782 m, disturbed montane rainforest (BLF) (CAS); Parc naturel communautaire, 26.8 km SW Ambositra, –20.775, 47.18362, 1755 m, disturbed montane rainforest (BLF) (CAS); Parc naturel communautaire, 28.5 km SW Ambositra, –20.78414, 47.16699, 1780 m, disturbed montane rainforest along road side (BLF) (CAS); Mampiarika IV Non Protected Area, 27.98 km SW Ambositra, –20.73528, 47.08382, 1486 m, Uapaca woodland (ARA) (CAS); R.S. Ivohibe, 6.5 km ESE Ivohibe, –22.49667, 46.955, 1575 m, montane rainforest (B.L. Fisher (Sylvain)) (CAS); P. N. Andringitra, Forêt Ravaro 12.5 km SW Antanifotsy, –22.21167, 46.845, 1650 m, montane rainforest (S. Razafimandimby) (CAS); R.S. Ivohibe 8.0 km E Ivohibe, –22.48333, 46.96833, 1200 m, montane rainforest (B.L. Fisher (Sylvain)) (CAS); 28 km. SSW Ambositra, Ankazomivady, –20.775, 47.16833, 1670 m, montane rainforest edge (B.L. Fisher) (CAS); 38 km S Ambalavao, Res. Andringitra, –22.2, 46.96667, 1680 m, montane rainforest (B.L. Fisher) (CAS); 40 km S Ambalavao, Res. Andringitra, –22.21667, 46.96667, 1275 m, montane rainforest (B.L. Fisher) (CAS); Res. Andringitra; 8.5 km SE Antanitotsy, –22.16667, 46.96667, 1990 m, montane rainforest (Sylvian) (CAS); 8.0 km NE Ivohibe, –22.42167, 46.89833, 1200 m, montane rainforest (B.L. Fisher (Sylvain)) (CAS); Antapia I Non Protected Area, 26.43 km SW Ambositra, –20.71972, 47.08685, 1495 m, Uapaca woodland (ARA) (CAS); Antohatsahomby I Non Protected Area, 22.77 km NW Ambatofinandrahana, –20.55056, 46.58562, 1550 m, Uapaca woodland (ARA) (CAS); Amoron’i Mania Region, District of Ambositra, Italaviana Uapaca forest, 135 Km SE of Antsirabe, –20.17333, 47.086, 1359 m, Uapaca forest, (MG) (CAS); Antapia IV Non Protected Area, 26.42 km SW Ambositra, –20.71917, 47.0868, 1494 m, Uapaca woodland (ARA) (CAS); Antohatsahomby V Non Protected Area, 22.63 km NW Itremo, –20.56722, 46.57923, 1726 m, Uapaca woodland (ARA) (CAS); Manombo Special Reserve, 32 km SE of Farafangana, –23.02183, 47.72, 36 m, lowland rainforest (MG) (CAS); Fitovavy Fitovinany Region, District of Ifanadiana, 12 km W of Ranomafana, –21.25083, 47.40717, 1127 m, forest edge, open area (MG) (CAS); Antohatsahomby IV Non Protected Area, 22.67 km NW Itremo, –20.56306, 46.58097, 1708 m, Uapaca woodland (ARA) (CAS); Ambinanindranomena Non Protected Area, 39.45 km SE Ambalavao, –21.95386, 47.29427, 1069 m, montane rainforest (ARA) (CAS); Hte Mananjary; Ambinanindrano; Ambositra, –20.733334, 47.633335, 657 m (R. Catala) (MNHN).

**Province Mahajanga**: Sofia Region, District of Sofia, Anjiamangirana 45 km S Antsohihy, Analagnambe Galery forest, 5 km W Anjiamangirana, –15.157, 47.73417, 97 m, low degraded dry forest (MG) (CAS). **Province Toamasina**: Ambanizana, Parc National Masoala, –15.57222, 50.00694, 1020 m, montane rainforest (D. Andriamalala, D. Silva et al.) (CAS); Moramanga, –18.948902, 48.224426, 914 m (Decarpentries) (NHMB); 6.9 km NE Ambanizana, Ambohitsitondroina, –15.58506, 50.00952, 825 m, rainforest (B.L. Fisher) (CAS); Montagne d’Anjanaharibe, 19.5 km 27° NNE Ambinanitelo, –15.17833, 49.635, 1100 m, montane rainforest (FGAT) (CAS). **Province Toliara**: [S.W. Madagascar]; Beronono; Mahabo, –21, 45, 339 m (Voltzkow) (NHMB); Androy Region, District of Tsihombe, 74 km S of Tsihombe, Cap Ste Marie Reserve, –25.58767, 45.163, 36 m, spiny bush (MG) (CAS); 13 km NW Enkara, Res Andohahela, –24.55, 46.8, 1300 m, montane rainforest (B.L. Fisher) (CAS); Atsimo Andrefana Region, District of Tulear II, Mikea decidious dry forest 3 km N Andranomavo village, –22.90367, 43.4755, 30 m, deciduous dry forest (MG) (CAS); Miary,–23.311972, 43.740917, 55 m (F. Geay) (MNHN); Ambovombe, –25.1775, 46.09283, 25 m (R. Decary) (MNHN); near road, Zombitse National Park, –22.8405, 44.73117, 825 m, spiny deciduous forest (R. Harin’Hala) (CAS).

#### ﻿ Camponotus norvigi sp. nov.

Taxon classificationAnimaliaHymenopteraFormicidae

561C14F8-1F15-5920-AD6C-6310C917395C

http://zoobank.org/E3EEFF4B-8BD3-4EEB-A15B-CC11DA614755

Figures 15A , 53

##### Holotype worker.

Madagascar, Province Mahajanga, Region Sofia, Bemanevika, –14.337, 48.58874, 1606 m, montane rainforest, ex dead twig above ground, 14 January 2015 (BLF), collection code: BLF35614, specimen code: CASENT0923254 (CAS). **Paratypes.** Three workers with the same data as holotype: collection code: BLF35657, specimen code: CASENT0724324; collection code: BLF35614, specimen code: CASENT0724356; collection code: BLF35615, specimen code: CASENT0724357 (CAS).

##### Worker diagnosis.

Integument black; head, mesosoma, and gastral dorsum clothed with dense, whitish hairs; anterior margin of clypeus with short rectangular lobe; dorsal outline of mesosoma almost flat; dorsum and declivitous faces of propodeum unequal in length and form an obtuse angle.

##### Description minor worker.

Medium-sized species. Absolute cephalic size (CS: 1.45±0.24, 1.31–1.66). In full-face view, head more quadrate (CWb/CL: 0.36±0.02; 0.35–0.38), posterior margin of head and lateral margin weakly convex. Eyes circular, placed laterally close to lateral head margin (PoOC/CL: 0.08±0.01; 0.07–0.09). Mandibles triangular with six teeth. Clypeus convex, its anterior portion projected into a rectangular lobe. Antennal scape circular and long (SL/CS: 0.40±0.03; 0.37–0.42). In lateral view, mesosoma low and long, its dorsum smoothly arcuate, metanotal suture obsolete. Humeral angle rounded. Propodeal dorsum about the same length as declivitous face (MPH/ML: 0.18±0.02; 0.18–0.20). In lateral view, petiole squamiform with blunt edge, anterior face short and converging to the highest point of the petiole, posterior face straight. Ventral face of femora marginate laterally, the outer margin of tibia with long, whitish setae. Head and mesosoma coarsely reticulate-punctate, legs and gastral tergite finely imbricate. Mandible finely foveolate with sparse punctures. Pilosity and pubescence whitish, the former sparse and suberect, especially on occipital portion, frontal area, and mesosoma, though most conspicuous on the thoracic dorsum. On the gaster, the suberect hairs are long and abundant, overlapping with appressed hairs, pubescence inconspicuous and short. Petiolar node fringed with one row of whitish hair from the level of petiolar spiracle. Head including scape, mesosoma, and gaster dark brown to black, legs brownish, less dark than mesosoma.

##### Description of major worker.

Characteristics of minor workers, except: head much wider posteriorly (CS: 1.98±0.30, 1.78–2.32; CWb/CL: 0.99±0.01, 0.98–1.00); lateral margins convex and converging towards base of mandible. Eyes semicircular, placed dorsolaterally midway from the occipital margin (PoOC/CL: 0.23± 0.01, 0.22–0.23). Clypeus much more quadrate in full-face view (ClyL/GPD: 0.72±0.02, 0.69–0.73), its anterior margin slightly concave medially. Antennal scape short, just surpassing the occipital border (SL/CS: 0.90±0.14, 0.75–1.00). Dorsal outline of mesosoma forms a continuous line so that the basal portion of propodeum forms an obtuse angle with the declivity. Lateral portion of head finely reticulate above, punctate to finely areolate below.

**Figure 53.**
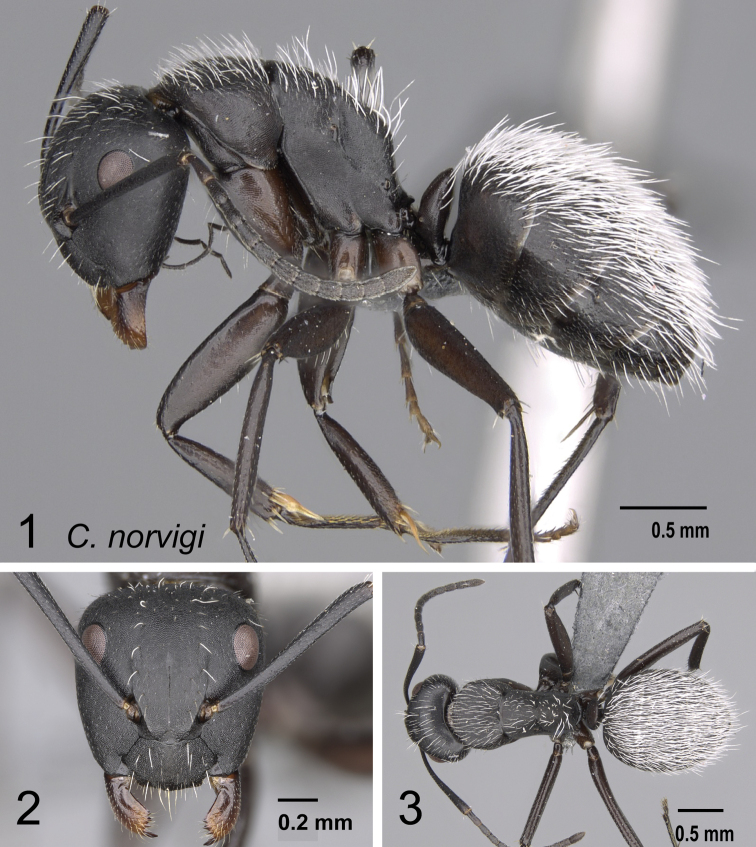
*Camponotusnorvigi* minor worker (CASENT0923254) **1** body in lateral view **2** head in full-face view **3** body in dorsal view.

##### Distribution and biology.

*Camponotusnorvigi* has been collected primarily by Malaise trap and beating low vegetation, and rarely from yellow pan traps. No colonies have been collected. It is distributed mainly in montane forest, rainforest, and high-altitude rainforest in southern, western, and central Madagascar; in tropical dry forest in northern Madagascar, e.g., Res Tsingy de Bemaraha, and in gallery forest and open areas on the western portion of the island (Fig. 58B). It occurs at elevations of 20–1606 meters. This species is sympatric with *C.darwinii* at the edge of PN Isalo and Ranomafana, at Ambohitantely (Forêt sclerophylle).

##### Discussion.

*Camponotusnorvigi* stands out within the species group. The color and disposition of the erect hairs covering its entire dorsum separate *C.norvigi* from *C.darwinii.* The latter species and *C.ursus* both have dense pubescence on their mesosoma or gastral dorsum, a character absent in *C.norvigi*. At first glance, *C.norvigi* appears closely related to the *C.madagascarensis* group because of its whitish pilosity, but the two differ in the structure of the mesosoma, which is low and long in the *C.madagascarensis* group and short and high in the *C.darwinii* group.

##### Etymology.

This species is named in honor of K. Norvig for support in training young scientists in Madagascar.

##### Additional material examined.

**Province Antananarivo**: Analamanga Region,District of Ankazobe, Ambohitantely, 46 km NE of Ankazobe, –18.198, 47.2815, 701 m, Forêt sclerophylle (MG) (CAS); Reserve Speciale d’Ambohitantely, –18.22444, 47.2774, 1490 m, montane forest (B.L. Fisher et al.) (CAS). **Province Antsiranana**: 9.2 km WSW Befingotra, Res. Anjanaharibe-Sud, –14.75, 49.46667, 1200 m, montane rainforest (B.L. Fisher) (CAS); Parc National de Marojejy, Antranohofa, 26.6 km 31° NNE Andapa, 10.7 km 318° NW Manantenina, –14.44333, 49.74333, 1325 m, montane rainforest (B.L. Fisher) (CAS); Parc National Montagne d’Ambre [Petit Lac road], –12.533333, 49.166668, 1125m, rainforest (R. Harin’Hala) (CAS). **Province Fianarantsoa**: 2 km NE Anjoma-Ramartina, –19.63333, 45.96667, 750 m, grassland (PSW) (CAS) ; 2 km W Andrambovato, along river Tatamaly, –21.51167, 47.41, 1075 m, cultivated land (tavy), recent 1–2 year-old tavy (BLF) (CAS); Ambalavao, –21.83267, 46.93867, 1020 m, urban/garden (BLF) (CAS); Belle Vue trail, Ranomafana National Park, –21.2665, 47.42017, 1020 m, mixed tropical forest (R. Harin’Hala) (CAS); Fitovavy Fitovinany Region, District of Ifanadiana Belle vue area, 1200 m S of Ranomafana National Park entrance, –21.2665, 47.42017, 1018 m, rainforest (MG) (CAS); Fitovavy Fitovinany Region, District of Ifanadiana, 12 km W of Ranomafana, –21.25083, 47.40717, 1127 m, forest edge, open area, (MG) (CAS); JIRAMA water works near river, Ranomafana National Park, Fianarantsoa Prov., –21.2485, 47.45217, 690m, open area near stream (R. Harin’Hala) (CAS); radio tower, Ranomafana National Park, –21.25833, 47.40717, 1130 m, forest edge, mixed tropical forest, open area (MG) (CAS); Ranomafana, –21.25, 47.36667 (A. Pauly) (CAS); Ranomafana, nr. Ifanadiana, –21.26667, 47.45, 610 m, open forest/shrubland (PSW) (CAS); stream area, 900 m E of Isalo National Park Interpretive Center, –22.62667, 45.35817, 750 m, open area near stream (R. Harin’Hala) (CAS); Vohiparara broken bridge, –21.22617, 47.36983, 1110 m, high altitude rainforest (R. Harin’Hala) (CAS). **Province Mahajanga**: Parc National de Namoroka, 16.9 km 317° NW Vilanandro, –16.40667, 45.31, 100 m, tropical dry forest (FGAT) (CAS); Parc National Tsingy de Bemaraha, 2.5 km 62° ENE Bekopaka, Ankidrodroa River, –19.13222, 44.81467, 100 m, tropical dry forest on Tsingy (FGAT) (CAS); Region Sofia, Bemanevika, –14.337, 48.58874, 1606 m, montane rainforest (BLF) (CAS). **Province Toamasina**: Ankerana, –18.40636, 48.80254, 1108 m, montane forest (BLF) (CAS); Corridor Forestier Analamay-Mantadia, Tsaravoniana, –18.76465, 48.41938, 1039 m, rainforest (BLF) (CAS); Toamasina Forêt Ambatovy, 14.3 km 57° Moramanga, –18.85083, 48.32, 1075 m, montane rainforest (B.L. Fisher) (CAS); Manakambahiny Atsinanana, –17.75, 48.71667, primary forest (A. Pauly) (CAS); P.N. Mantadia, –18.79167, 48.42667, 895m, rainforest (H.J. Ratsirarson) (CAS); Torotorofotsy, –18.77048, 48.43043, 1005 m, montane rainforest (B.L. Fisher et al.) (CAS). **Province Toliara**: 13 km NW Enakara, Res. Andohahela, –24.55, 46.8, 1180 m, montane rainforest (B.L. Fisher) (CAS); 2.7 km WNW 302° Ste. Luce, –24.77167, 47.17167, 20 m, littoral rainforest (B.L. Fisher) (CAS); 4 km N Isaka-Ivondro, –24.76667, 46.86667, 180 m, roadside (PSW) (CAS); 6 km ESE Imonty, Res. Andohahela, –24.85, 46.75, 1200 m, rainforest (PSW) (CAS); Makay Mts., –21.227, 45.33222, 475 m, gallery forest on sandy soil (BLF) (CAS); Manatantely, 8.5 km NW Tolagnaro, –24.9875, 46.92617, 85 m, rainforest (BLF) (CAS); Parc National Andohahela, Col de Tanatana, 33.3 km NW Tolagnaro, –24.7585, 46.85367, 275 m, rainforest (BLF) (CAS); Parc National d’Andohahela, Col du Sedro, 3.8 km 113° ESE Mahamavo, 37.6 km 341° NNW Tolagnaro, –24.76389, 46.75167, 900 m, montane rainforest (FGAT) (CAS); Res. Andohahela, 6 km SSW Eminiminy, –24.73333, 46.8, 330 m, rainforest (PSW) (CAS); Reserve Speciale d’Ambohijanahary, Forêt d’Ankazotsihitafototra, 35.2 km 312° NW Ambaravaranala, –18.26667, 45.40667, 1050 m, montane rainforest (FGAT) (CAS).

#### ﻿ Camponotus nossibeensis

Taxon classificationAnimaliaHymenopteraFormicidae

André

643A9FA7-C8CA-5C60-BD48-BB038098503A

**Figure 54.**
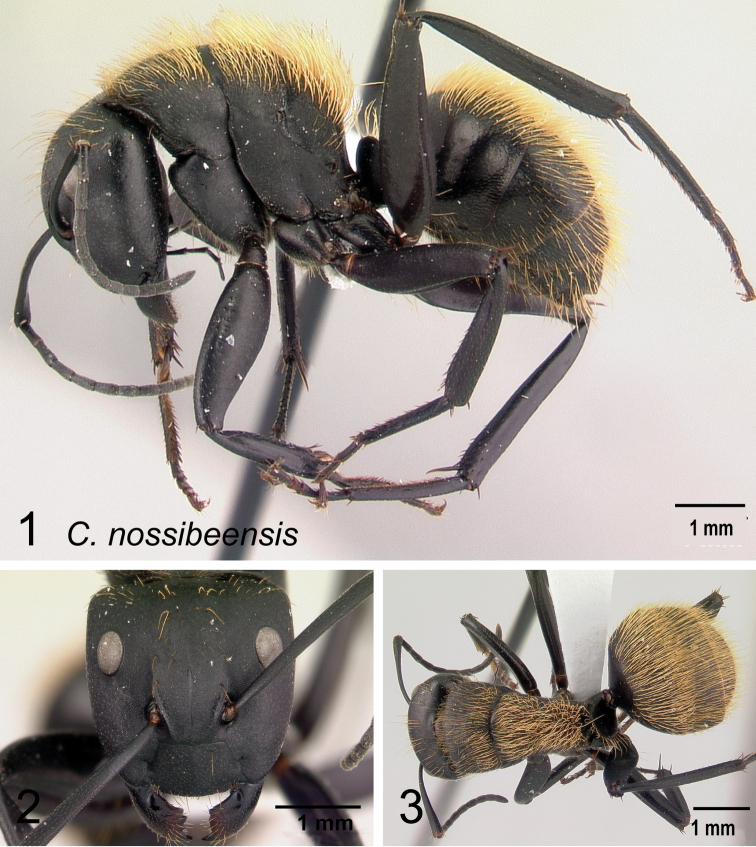
*Camponotusnossibeensis* minor worker (CASENT0191657) **1** body in lateral view **2** head in full-face view **3** body in dorsal view.

Figures 5C , 13A , 14A , 54

Camponotus nossibeensis André, 1887: 281. Lectotype major worker, present designation, Madagascar, Province Antsiranana, Nossi-be, –13.291667, 48.258335, 146 m (Forel), AntWeb CASENT0101425 (MNHN). Paralectotype minor worker, same data as lectotype but specimen coded as CASENT0101373 (MHNG).
Camponotus (Myrmobrachys) nossibeensis Forel, 1912: 91; 1914: 270.
Camponotus (Myrmepomis) nossibeensis Emery, 1920: 258.
Camponotus (Myrmopiromis) nossibeensis Wheeler, 1922: 1052; Emery, 1925: 129; Bolton, 1995: 114, 131.

##### Worker diagnosis.

Integument matte black; body a rust brown color, relatively dense on entire dorsum; anterior margin of clypeus with short rectangular lobe; dorsal outline of mesosoma weakly arcuate; declivitous face of propodeum longer than its dorsum.

##### Description of minor worker.

Large-sized species. Absolute cephalic size (CS: 2.24 0.46; 1.92–2.70). In full-face view, head slightly longer than broad, narrower in front than behind, with nearly straight posterior and lateral margins. (CWb/CL: 0.37±0.03; 0.35–0.42). Eyes more elongate, elliptical, not breaking outlines of head (PoOC/CL: 0.08±0.02; 0.06–0.09). Mandibles triangular withsix teeth. Clypeus carinate, anterior clypeal margin entire, projecting into a rectangular lobe with an obtuse anterolateral angle (ClyL/GPD: 0.28±0.05, 0.24–0.32). Antennal scape long, surpassing the occiput by the length of one funiculus (SL/CS: 0.39±0.05; 0.33–0.41). In lateral view, mesosoma short, robust, and depressed; its dorsal outline arcuate; promesonotal suture large, distinctly shiny, and glabrous; humeral angle distinctly rounded; metanotal suture obsolete; propodeal dorsum slightly convex in profile and distinctly shorter than the declivitous face into which it passes via a rounded obtuse angle (MW/ML: 0.22±0.03; 0.20–0.24; MPH/ML: 0.21±0.05; 0.18–0.24). In lateral view, petiolar node squamiform, anterior face confused with its dorsal face, node summit truncate, rounded triangle in dorsal view. Head, mesosoma, and gastral tergite finely and densely punctate. Mandible finely and longitudinally striate with sparse punctuation. Hairs golden yellow, erect to suberect, abundant, bending forward at its midlength on occipital region and mesosomal dorsum, but more abundant on mesonotum and propodeum dorsum; sparse, stiff, pointed, and a different length on gastral tergites. Petiole along its border with a fringe of golden, yellowish hairs not the same length. Body entirely black and a little shiny; masticatory margin reddish to dark brown.

##### Description of major worker.

With characteristics of minor worker, except: head much wider posteriorly (CS: 3.05±0.41, 2.35–3.67; CWb/CL: 1.02±0.04, 0.96–1.08), lateral margin almost straight and tapering anteriorly to the mandibular insertion. Eyes elliptical, moderately sized compared to the head (EL/CS: 0.20±0.01, 0.1––0.23), placed dorsally next to the vertex (PoOC/CL: 0.23± 0.02, 0.18–0.27). Clypeus more rectangular (ClyL/GPD: 0.84±0.05; 0.73–0.91). Antennal scape just surpassing the occipital margin (SL/CS: 0.77±0.08; 0.68–0.91). Mandible strongly built with five large teeth, apical tooth sharp and long, and remainder decreasing in size to the basal margin. Mesosoma, pilosity, and pubescence same as minor worker. Head shiny black; anterior portion of head, including frontal area, finely alveolate punctate.

##### Distribution and biology.

*Camponotusnossibeensis* is an endemic species mostly known from the northern part of Madagascar, from Nosy Faly to Ambanja. This species seems very adaple because it has been collected from both humid and tropical dry forest, at altitudes 7–780 m (Fig. 58C). The data indicate that individual workers forage on the ground and on lower vegetation, while nests are mostly found in dead twigs above ground, rotten logs, dead tree stumps, and underground.

##### Discussion.

The combination of large size, big eyes, vertical declivitous face of propodeum, dense pilosity on mesosoma and gaster, and alveolate sculpture make *C.nossibeensis* a very distinctive species.

##### Additional material examined.

**Province Antsiranana**: Forêt Ambato, 26.6 km 33° Ambanja, –13.4645, 48.55167, 150 m, rainforest (B.L. Fisher) (CAS); Nosy faly, Tafiambotry, 35.3 km N Ambanja, –13.3654, 48.48775, 7 m, littoral rainforest (BLF) (CAS); Ankobahoba, 32.3 km N Ambanja, –13.39166, 48.48249, 41 m, disturbed littoral rainforest (BLF) (CAS); R.S. Manongarivo, 10.8 km 229° SW Antanambao, –13.96167, 48.43333, 400 m, rainforest (CAS); R.S. Manongarivo, 12.8 km 228° SW Antanambao, –13.97667, 48.42333, 780 m, rainforest(CAS); R.S. Manongarivo, 20.4 km 219° SW Antanambao, –14.04667, 48.40167, 1860 m, montane rainforest (CAS); R.S. Manongarivo 17.3 km 218° SW Antanambao, –14.02167, 48.41833, 1580 m, montane rainforest (B.L. Fisher) (CAS); Nosy Faly, –13.36435, 48.49137, 40 m, open secondary vegetation (BLF) (CAS); Nosy Faly, –13.3624, 48.49101, 15 m, open secondary vegetation (BLF) (CAS); Sahamalaza Peninsula, Forêt d’Anabohazo, 21.6 km 247° WSW Maromandia, –14.30889, 47.91433, 120 m, tropical dry forest (FGAT) (CAS); Nosy Be, Réserve Naturelle Intégrale de Lokobe, 6.3 km 112° ESE Hellville, –13.41933, 48.33117, 30 m, rainforest (FGAT) (CAS); Sahamalaza National Park, –14.16361, 47.70277, 192 m, subhumid forest (MG) (CAS); Nosy Faly, –13.36255, 48.49104, 19 m, open secondary vegetation (BLF) (CAS). **Province Mahajanga**: Parc National Tsingy de Bemaraha, 3.4 km 93° E Bekopaka, Tombeau Vazimba, –19.14194, 44.828, 50 m, tropical dry forest (FGAT) (CAS); Parc National Tsingy de Bemaraha, 10.6 km ESE 123° Antsalova, –18.70944, 44.71817, 150 m, tropical dry forest on Tsingy (FGAT) (CAS); Parc National Tsingy de Bemaraha, 2.5 km 62° ENE Bekopaka, Ankidrodroa River, –19.13222, 44.81467, 100 m, tropical dry forest on Tsingy (FGAT) (CAS); Réserve Spéciale Marotandrano, Marotandrano 48.3 km S Mandritsara, –16.28322, 48.81443, 865 m, transitional humid forest (BLF) (CAS); Ampamakiambato, 45 km SW Ambanja, –13.97545, 48.15929, 145 m, disturbed forest, in tsingy (BLF) (CAS); Manerinerina, 76.6 km N Antsohihy, –14.10744, 48.11046, 247 m, disturbed forest (BLF) (CAS); Réserve forestière Beanka, 50.7 km E Maintirano, –17.88021, 44.46877, 140 m, tropical dry forest on tsingy (BLF) (CAS); Réserve forestière Beanka, 54.3 km E Maintirano, –18.06009, 44.54086, 262 m, tropical dry forest on tsingy (BLF) (CAS); Melaky Region, District of Besalampy, Marofototra dry forest, 17 km W of Besalampy, –16.72167, 44.42367, 51 m, dry wash in the dry forest (MG) (CAS); Bemeraha, 9 km E. Antsalova, –18.65, 44.71667 (D.C. Lees) (PSWC); Forêt de Tsimembo, 8.7 km 336° NNW Soatana, –19.02139, 44.44067, 20 m, tropical dry forest (FGAT) (CAS). **Province Toliara**: Belalanda, –23.29833, 43.64648, 5 m, spiny forest/thicket, wetlands (Frontier Wilderness Project) (CAS); near road, Zombitse National Park, –22.8405, 44.73117, 825 m, spiny deciduous forest (R. Harin’Hala) (CAS); near ANGAP office, Zombitse National Park, –22.8865, 44.69217, 840 m, deciduous spiny forest (R. Harin’Hala) (CAS); 15 km E Sakaraha, –22.9, 44.68333, 760 m, tropical dry forest (PSW) (PSWC); Makay Mts., –21.30997, 45.12946, 590 m, Dry forest on sandy soil (BLF) (CAS); Makay Mts., –21.227, 45.33222, 475 m, gallery forest on sandy soil (BLF) (CAS); Makay Mts., –21.21013, 45.35462, 540 m, gallery forest with bamboo (BLF) (CAS); Parc National de Zombitse, 17.7 km 98° E Sakaraha, –22.88833, 44.70167, 760 m, tropical dry forest (FGAT) (CAS); 9 km NNW Ranohira, P.N. Isalo, –22.48333, 45.38333, 800 m, tropical dry forest (PSW) (PSWC).

#### ﻿ Camponotus radovae

Taxon classificationAnimaliaHymenopteraFormicidae

Forel, 1886

90859E1B-4168-588A-97CB-8325A074D9A9

**Figure 55.**
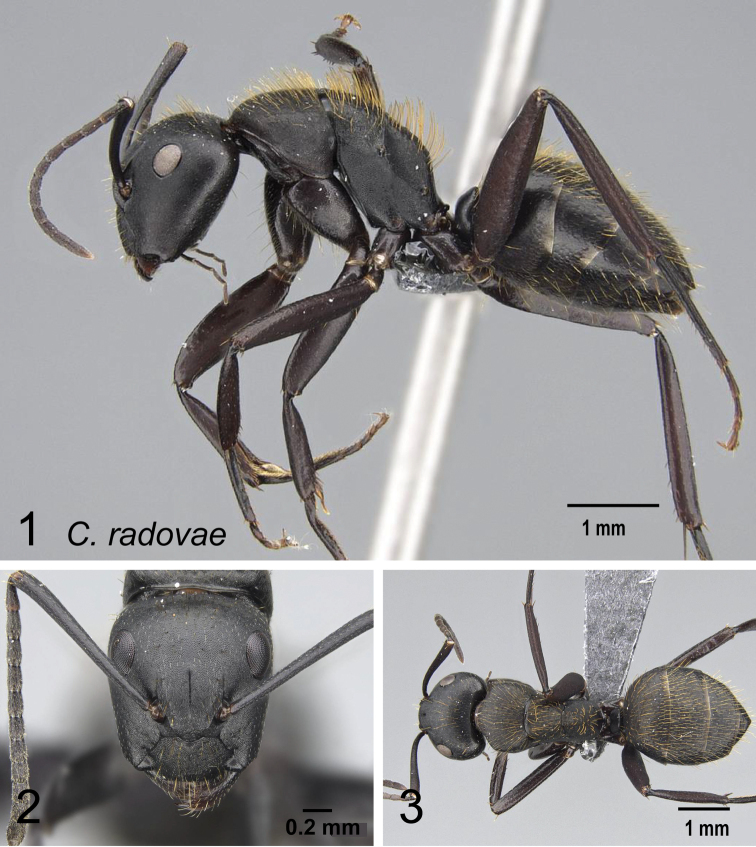
*Camponotusradovae* minor worker (CASENT0066208) **1** body in lateral view **2** head in full-face view **3** body in dorsal view.

Figures 14B , 16D , 18C, D , 55

Camponotus radovae Forel, 1886: civ. Lectotype minor worker, present designation, Madagascar, Province Antananarivo (Camboué), AntWeb CASENT0101363 (MHNG). Paralectotypes two workers with the same data as lectotype but specimen coded as CASENT0101362, CASENT0101364 (MHNG).
Camponotus (Myrmobrachys) radovae Forel, 1912: 91.
Camponotus (Myrmepomis) radovae Emery, 1920: 258.
Camponotus (Myrmopiromis) radovae Wheeler, 1922: 1052; Emery, 1925: 129; Bolton, 1995: 119, 131.
Camponotus radovae-darwinii Forel, 1891: 46. Lectotype minor worker, present designation, Madagascar, Province Antananarivo (Camboué), AntWeb CASENT0101367 (MHNG). Paralectotype one worker with the same data as lectotype but specimen coded as CASENT0101115 (MHNG).
Camponotus (Myrmopiromis) radovae-darwinii Wheeler, 1922: 1053; Emery, 1925: 129; Bolton, 1995: 119. Syn, nov.

##### Worker diagnosis.

Integument matte black with sparse, yellowish brown hairs arranged in a series of transverse rows, pubescence reduced to absent on mesosomal dorsum. Anterior clypeal margin with a distinct produced rectangular lobe. Petiole cuneate in profile.

##### Description of minor worker.

Medium-sized species. Absolute cephalic size (CS: 1.59±0.22, 1.43–1.77). In full-face view, head longer than broad, narrower in front than in back, with feebly convex occipital and lateral sides (CWb/CL: 0.37±0.03; 0.35–0.40). Eyes circular, placed much closer to the lateral sides (PoOC/CL: 0.08±0.01, 0.07–0.09). Mandibles triangular with six teeth. With head in full-face view, clypeus not carinate, its anterior margin produced, forming a short rectangular lobe with a sharp, lateral angle. Antennal scape circular, long, surpassing the occiput by the length of one basal funiculus (SL/CS: 0.39±0.03, 0.37–0.41). In lateral view, anterodorsal corner of pronotum broadly rounded, mesosomal dorsal almost straight, propodeal dorsum shorter than declivity, both straight in profile and meeting at a blunt angle (MW/ML: 0.21±0.02, 0.20–0.23; MPD/ML: 0.24±0.02, 0.22–0.26). Promesonotal and metanotal sutures form bold, polished lines. In lateral view, petiolar node cuneate, with a short, vertical, anterior face which reaches toward the flattened posterior faces. Head and mesosoma finely alveolate, gaster more finely strigulate. Mandible finely foveate with sparse punctures. Pilosity of dorsal head and body consists of fine and pointed brownish yellow hairs, long and suberect on frontal area and mesosomal dorsum; mostly short and sparse on gastral tergites; petiolar dorsum edge with 4 to 5 pairs of brownish yellow hairs. Body entirely matte black, apical portion of mandible, basitarsi, and funiculus reddish brown.

##### Description of major worker.

Characteristics of minor workers, except: head somewhat cordate, with nearly straight posterior margin and slightly convex lateral sides (CS: 2.76±0.12, 2.59–2.85; CWb/CL: 1.05±0.02, 1.02–1.07). Eyes elliptical, placed dorsally midway from the occipital margin (PoOC/CL: 0.24±0.01, 0.23–0.25). Clypeus much more quadrate in full-face view with truncate anterior margin (ClyL/GPD: 0.76±0.13, 0.57–0.86). Antennal scape short, just surpassing the occipital border (SL/CS: 0.74±0.03, 0.72–0.79). Dorsal outline of mesosoma interrupted by pronotal and mesonotal sutures, basal portion of propodeum forms an obtuse angle with the declivity. Lateral portion of head finely punctate, frontal area and vertex with deep punctures.

##### Distribution and biology.

*Camponotusradovae* is mainly distributed in the southern part of Madagascar in Zombitse National Park (Fig. 58D). This species is encountered in native forest habitats such as deciduous dry forest, tropical dry forests, gallery forests, and spiny forests and thickets. It was sampled mostly from Malaise traps and by beating lower vegetation, on the ground, and twice on leaf litter at altitudes 20–840 m.

##### Discussion.

Within the *Camponotusdarwinii* group, *Camponotusradovae* cannot be confused with *C.darwinii* or *C.themistocles* since the latter two differ by size and pilosity color. However, *C.radovae* has reduced pubescence and is smaller in size.

##### Additional material examined.

**Province Toliara**: Atsimo Andrefana Region, District of Sakaraha, Zombitse National Park; 900 m N from ANGAP Entrance office, –22.8405, 44.73117, 823 m, spiny deciduous forest (MG) (CAS); Atsimo Andrefana Region, District of Toliara II, Mikea decidious dry forest 3 km N Andranomavo village, –22.90367, 43.4755, 30 m, Deciduous dry forest (MG) (CAS); Atsimo Andrefana Region, District of Toliara II, Mikea spiny forest 8 km N Andranomavo village, –22.91333, 43.39883, 37 m, spiny forest (MG) (CAS); Mikea Forest, deciduous dry forest, –22.90367, 43.4755, 30 m, deciduous dry forest (R. Harin’Hala) (CAS); Mikea Forest, spiny forest, –22.91333, 43.48222, 37 m, spiny forest (R. Harin’Hala) (CAS); near ANGAP office, Zombitse National Park, –22.8865, 44.69217, 840 m, deciduous spiny forest (R. Harin’Hala) (CAS); near road, Zombitse National Park, –22.8405, 44.73117, 825 m, spiny deciduous forest (R. Harin’Hala) (CAS); Parc National de Zombitse, 17.7 km 98° E Sakaraha, –22.88833, 44.70167, 760 m, tropical dry forest (FGAT) (CAS); Parc National de Zombitse, 19.8 km 84° E Sakaraha, –22.84333, 44.71, 770 m, tropical dry forest (FGAT) (CAS); Ranobe, –23.03975, 43.6109, 30 m, spiny forest/thicket (Frontier Project) (CAS); Ranobe, –23.03957, 43.61032, 30 m, spiny forest/thicket (Frontier Project) (CAS); Ranobe, –23.03957, 43.61017, 30 m, spiny forest/thicket (Frontier Project) (CAS); Ranobe, –23.03883, 43.60982, 30 m, gallery forest (Frontier Wilderness Project) (CAS); Ranobe, –23.03952, 43.61015, 20 m, gallery forest (Frontier Wilderness Project) (CAS); Ranobe, –23.03832, 43.6096, 20 m, gallery forest (Frontier Wilderness Project) (CAS).

#### ﻿ Camponotus themistocles

Taxon classificationAnimaliaHymenopteraFormicidae

Forel stat. nov.

02D9DC15-904D-5058-BCD8-DF2156D26ACC

**Figure 56.**
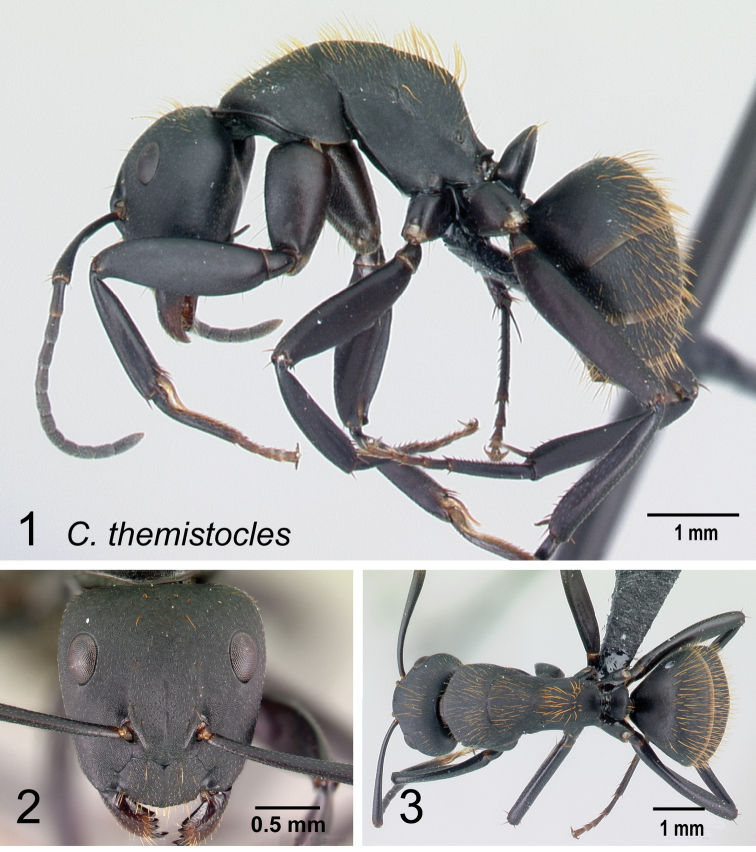
*Camponotusthemistocles* minor worker (CASENT0217297) **1** body in lateral view **2** head in full-face view **3** body in dorsal view.

Figures 5D , 15B , 16C , 18A, B , 56

Camponotus darwinii themistocles Forel, 1910: 456. Holotype minor worker, Madagascar, Fort Dauphin S. E. Madagascar, AntWeb CASENT0101381 (MHNG).
Camponotus (Myrmobrachys) darwinii themistocles Forel, 1914: 271.
Camponotus (Myrmepomis) darwinii themistocles Emery, 1920: 258. Stat.nov.
Subspecies of Camponotusdarwinii Santschi, 1911: 133.
Subspecies of Camponotus (Myrmopiromis) Wheeler, 1922: 1051; Emery, 1925: 128.

##### Worker diagnosis.

Integument dark brown with rust or red-brown suberect pilosity. Anterior clypeal margin with rectangular projection. Petiole scale thin with sharp border.

##### Description of minor worker.

Medium-sized. Absolute cephalic size (CS: 1.82±0.37; 1.53–1.98). In full-face view, head slightly longer than broad, a little narrower in front than in back, with straight occipital and feebly convex lateral margins (CWb/CL: 0.36±0.03; 0.34–0.38). Eyes circular, placed much closer to the lateral sides (PoOC/CL: 0.07±0.01; 0.07–0.08). Mandibles triangular with sixteeth. With head in full-face view, anterior clypeal margin produced, entire, and angularly projecting in the middle. Antennal scape circular, long, surpassing the occiput by the lengths of two basal funiculi (SL/CS: 0.41±0.05; 0.38–0.44). In lateral view, anterodorsal corner of pronotum broadly rounded, mesosomal dorsum moderately arcuate, propodeum dorsum a little shorter than the declivity, each straight in profile and meeting at an obtuse angle (MW/ML: 0.21±0.02, 0.20–0.22; MPD/ML: 0.24±0.03; 0.22–0.26). In lateral view, petiolar node wedge-shaped, with feebly convex anterior and flattened posterior faces. Ants entirely reticulate-punctate. Mandible finely striolate with sparse punctures. Pilosity and pubescence brownish yellow, the former long and curved forward especially on vertex, frontal area, and mesosomal dorsum; mostly short and sparse on gastral tergites; petiolar dorsum edge with four or five pairs of brownish yellow hairs. Body color matte black, apical portion of mandible reddish.

##### Description of major worker.

Characteristics of minor workers, except: head somewhat cordate, with nearly straight posterior margin and slightly convex lateral sides (CS: 2.68±0.36, 2.26–3.15; CWb/CL: 1.05±0.04, 0.99–1.11). Eyes elliptical, placed dorsally midway from the occipital margin (PoOC/CL: 0.23±0.01, 0.22–0.25). Clypeus much more quadrate in full-face view with truncate anterior margin (ClyL/GPD: 0.82±0.07, 0.73–0.93). Antennal scape short, just surpassing the occipital border (SL/CS: 0.75±0.03, 0.71–0.79). Dorsal outline of mesosoma interrupted by pronotal and mesonotal sutures, basal portion of propodeum forms an obtuse angle with the declivity. Lateral portion of head finely punctate, frontal area and vertex with deep punctures.

##### Distribution and biology.

*Camponotusthemistocles* has been collected at three localities in southern Madagascar (Fig. 58E): two are littoral forest and one is gallery forest. All sites are at low altitudes from 10 to 30 meters. *Camponotusthemistocles* is a ground nester; most collections have been made from sifted litter, rotten logs, and dead twigs above the ground.

##### Discussion.

*Camponotusthemistocles* differs from *C.radovae* by its larger size and the sculpture of the lateral sides *o*f its mesosoma, which is finely reticulate-punctate in the former and widely striolate in the later. In addition, in lateral view, the petiole scale of *C.themistocles* has an acute summit but is blunt in *C.radovae.* Pubescence is long and abundant in *C.themistocles*, and short and dilute in *C.radovae*. Morphometrical analysis reveals that *C.themistocles* and *C.ellioti* are the same size, but differ in integument coloration and pilosity pattern on the gastral tergite.

##### Additional material examined.

**Province Toliara**: Forêt de Petriky, 12.5 km W 272° Tolagnaro, –25.06167, 46.87, 10 m, littoral rainforest (B.L. Fisher) (CAS); Forêt Mandena 8.5 km N Tolagnaro, –24.95267, 47.0025, 20 m, littoral rainforest (BLF) (CAS); Mandena, 8.4 km NNE 30° Tolagnaro, –24.95167, 47.00167, 20 m, littoral rainforest (B.L. Fisher) (CAS); Ranobe, –23.04085, 43.61012, 30 m, gallery forest (Frontier Wilderness Project) (CAS).

#### ﻿ Camponotus ursus

Taxon classificationAnimaliaHymenopteraFormicidae

Forel

CC6DAF11-264F-5C95-B145-3B2E816ECD40

**Figure 57.**
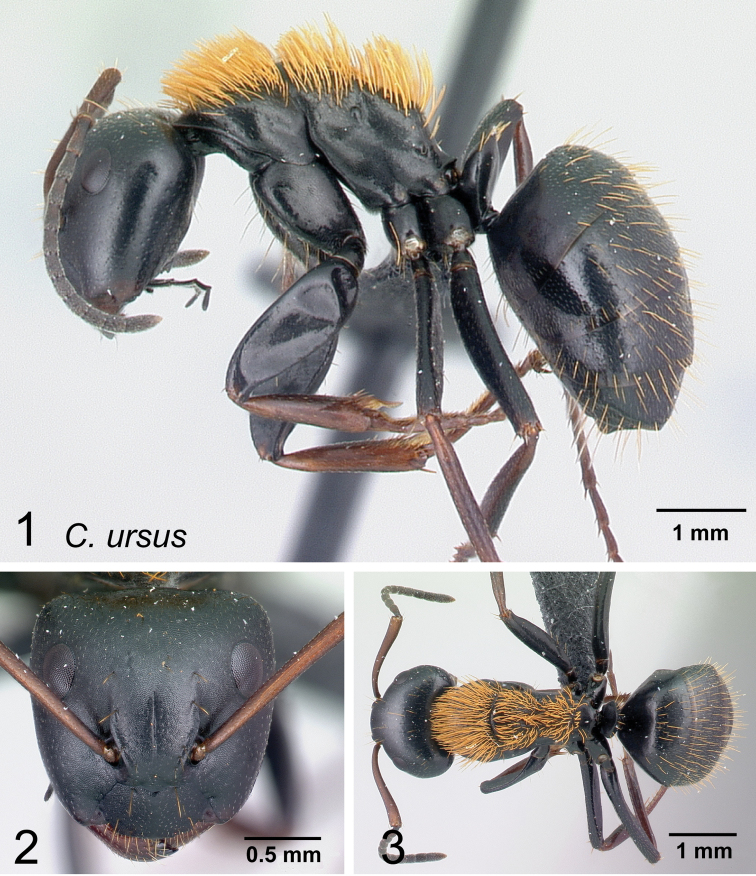
*Camponotusursus* minor worker (CASENT0217284) **1** body in lateral view **2** head in ful-face view **3** body in dorsal view.

Figures 2C , 16A , 17A , 57

Camponotus ursus Forel, 1886: ci. Lectotype minor worker, present designation, Madagascar (Forel), AntWeb CASENT0101374 (MHNG) [examined]. Paralectotype, one minor worker same data as lectotype but specimen coded as CASENT0101375 (MHNG).
Camponotus (Myrmobrachys) ursus Forel, 1912: 91; 1914: 271.
Camponotus (Myrmepomis) ursus Emery, 1920: 258.
Camponotus (Myrmopiromis) ursus Wheeler, 1922: 1053; Emery, 1925: 129; Bolton, 1995: 128, 131.

##### Worker diagnosis.

Integument shiny black; anterior margin of clypeus with short, rectangular lobe; dorsum of mesosoma with dense, decumbent, golden-yellow setae; gastral dorsum with widely distributed, short, subdecumbent setae.

##### Description of minor worker.

Medium-sized species. Absolute cephalic size (CS: 1.54±0.19; 1.43–1.64). In full-face view, head somewhat longer than broad, narrower in front than behind, with slightly convex lateral and posterior sides (CWb/CL: 0.37±0.02; 0.36–0.38). Eyes elliptical, sublateral at midpoint from the lateral sides (PoOC/CL: 0.07±0.02; 0.06–0.08). Mandibles triangular with six teeth. Clypeus not carinate, produced into a short, rectangular lobe. Antennal scape long, surpassing the occiput by the length of one basal funiculus. (SL/CS: 0.38±0.02; 0.37–0.39). In lateral view, dorsal contour of mesosoma smoothly convex, humeral angle broadly rounded; in dorsal view, mesonotal suture distinct but not impressed, mesonotal suture obsolete so that mesonotum and propodeum are fused together, propodeum with convex base and sloping declivity (MPH/ML: 0.18±0.02; 0.16–0.19). Petiole narrow, cuneate in profile, with short anterior face tapering dorsally to the flattened posterior face, its border rather sharp, produced upwards as a blunt angle in the middle. Head dorsum finely reticulate-punctate with shallow, sparse punctures; occipital region transversally striate-reticulate; lateral face of mesosoma, declivitous face, and petiolar face finely strigulate; legs finely reticulate; dorsum of mesosoma finely reticulate-striate-punctate with sparse excavation from which one suberect setae arises. Dorsum of gastral tergite finely striate-reticulate transversally, with sparse, small punctures. Mandible finely rugose with sparse, large punctures. Hairs golden yellow, the former abundant, long, and bending forward on entire mesosoma dorsum; suberect, short, and sparsely distributed on gastral tergites; the latter short and conspicuous on the abdominal segment, five pairs of erect hairs present on vertex. Body shiny black; scape, two basal funiculi, mandible, tarsi, and tibiae reddish.

##### Description of major worker.

Characteristics of minor workers, except: head as broad as long, with occipital and lateral margin almost straight (CS: 2.14±0.15, 2.00–2.39; CWb/CL: 0.89±0.07, 0.84–1.03). Eyes circular, placed dorsally next to the vertex (PoOC/CL: 0.23±0.01, 0.21–0.24). Anterior clypeal margin forms a short, rounded lobe (ClyL/GPD: 1.03±0.41, 0.73–1.78). Antennal scape short, just reaching the occipital border (SL/CS: 0.82±0.03, 0.78–0.86). Dorsal outline of mesosoma almost flat, propodeum dorsum naked and the same length as the sloping declivity.

##### Distribution and biology.

**Figure 58.**
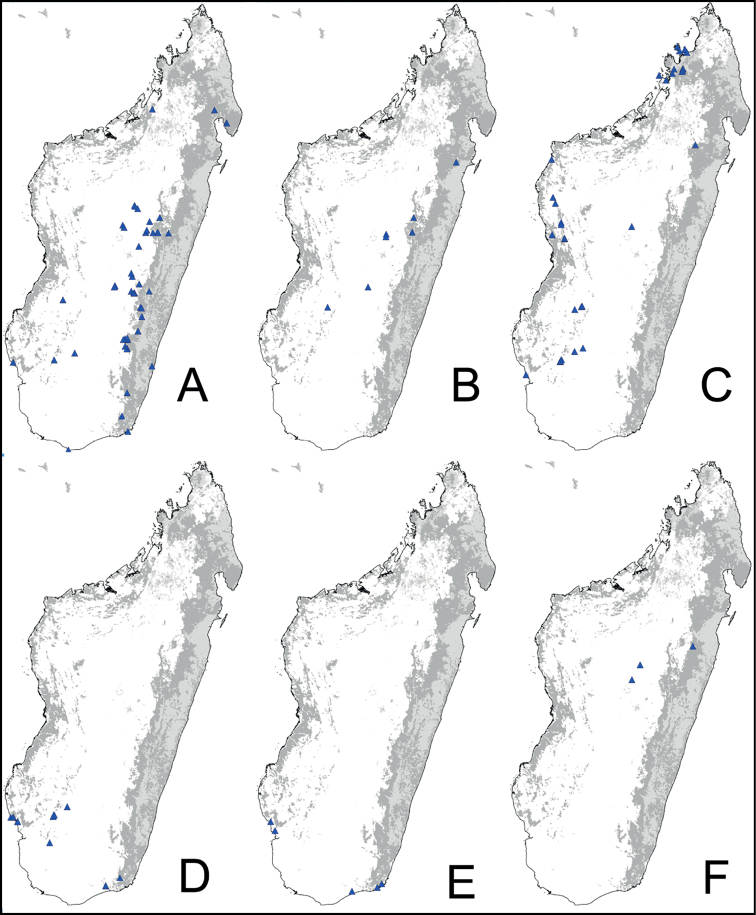
Distribution map of *C.darwinii* species group **A***C.darwinii***B***C.norvigi***C***C.nossibeensis***D***C.radovae***E***C.themistocles***F***C.ursus*

*Camponotusursus* is found in two different habitats: primary forest in the eastern portion and urban/garden areas in the central highlands of Madagascar (Fig. 58F). It is found foraging on low vegetation or inside branches above the ground. It occurs at altitudes above 1,200 meters.

##### Discussion.

*Camponotusursus* is recognizable within the *C.darwinii* species group on the basis of its distinct mesosomal pilosity. In addition, it is the only species with reddish brown basitarsi. However, *C.ursus* is unlikely be confused with *C darwinii* for several reasons. First, the body of the latter is much larger. Second, the head and gastral segment of *C.ursus* are covered with fine, short, sparse, and yellowish setae, giving the ant a glossy appearance that is completely different than that of *C.darwinii.*

##### Additional material examined.

**Province Antananarivo**: Ambatomanjaka; Miarinarivo, –18.766947, 46.869107, 1343 m (MHNG); Ankazobe, –18.31617, 47.11583, 1241 m (BLF) (CAS). **Province Toamasina**: Manakambahiny Atsinanana, –17.75, 48.71667 (A. Pauly) (CAS).

### The *Camponotusefitra* species group

#### ﻿ Camponotus chrislaini sp. nov.

Taxon classificationAnimaliaHymenopteraFormicidae

B1BA149D-AF57-5670-859E-C43BF1DA7E54

http://zoobank.org/0F5C0EBB-7F91-4FCC-839C-F8772D2BD9B5

**Figure 59.**
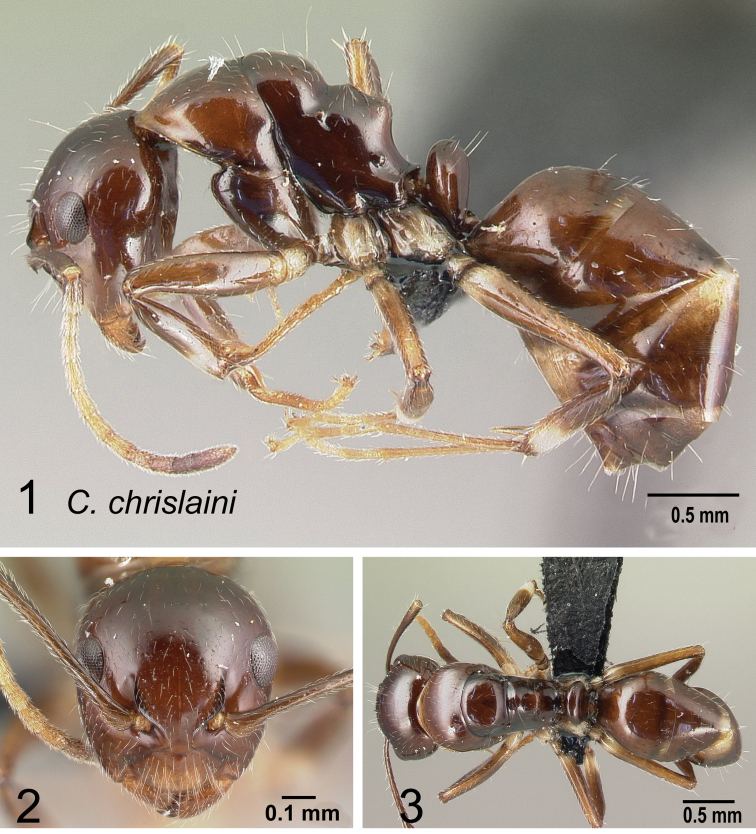
*Camponotuschrislaini* minor worker (CASENT0498999) **1** body in lateral view **2** head in full-face view **3** body in dorsal view.

Figures 2D , 7C–D , 41D , 59

##### Holotype worker.

Madagascar, Province Antsiranana, Forêt d’Andavakoera, 21.4 km 75° ENE Ambilobe; 4.6 km 356° N Betsiaka, –13.11833, 49.23, 425 m, rainforest, ex rotten log, 16 December 2003 (BLF), collection code: BLF10296, specimen code: CASENT0498999 (CAS). **Paratypes.** Nine workers with the same data as holotype, CASENT0499000, CASENT0499001, CASENT0499002 (three workers in each pin) (CAS).

##### Worker diagnosis.

*Camponotuschrislaini* can be easily distinguished from the other members of the *efitra* group by its mostly smooth and shiny sculpture; head more or less globose in full-face view; sigmoid frontal carina, and wavy propodeum dorsum.

##### Description of minor worker.

Medium-sized species. Absolute cephalic size (CS: 0.95 ± 0.17; 0.87–1.02). In full-face view, head ovoid (CWb/CL: 0.34±0.03; 0.32–0.35); posterior margin rounded, lateral margin of head convex. Eyes circular, protruding, placed midway from the occipital corner (PoOC/CL: 0.12±0.03; 0.11–0.13). Mandibles triangular with five teeth. Clypeus transversally trapezoidal, its anterior border broadly rounded, not angular. With head in full-face view, anterior clypeal margin rounded, not projecting (ClyL/GPD: 0.26±0.03, 0.25–0.29). Antennal scape flattened and lobulated at the base, long, surpassing the occiput by the length of three basal funiculi (SL/CS: 0.46±0.03; 0.45–0.48). In lateral view, anterodorsal corner of pronotum tuberculate, pronotal suture distinct, mesonotal suture distinctly impressed so that the anterior portion of propodeum is raised to a blunt edge in profile, and suture forms a concavity (MW/ML: 0.22±0.02, 0.21–0.23; MPH/ML: 0.17±0.02, 0.17–0.18). In lateral view, petiolar node nodiform, higher than long. Entire body, including mandible, smooth and shiny. One pair of standing hairs on angle formed by mesonotal dorsum and propodeum, head with scattered, subdecumbent, filiform brown hairs, scape with decumbent setae. Ant brownish; mandible, clypeus, and pronotum light brown; mid- and forecoxae, apical femora, basal tibiae, and apical portion of third and fourth tergite whitish.

##### Description of major worker.

Characteristics of minor workers, except: head quadrate with straight margins (CS: 1.67±0.06, 1.57–1.74; CWb/CL: 0.92±0.03, 0.90–0.96). Eyes circular, closed to the lateral margin (PoOC/CL: 0.29±0.02, 0.27–0.31). In profile, clypeus distinctly truncate (ClyL/GPD: 0.92±0.03, 0.86–0.94), its anterior margin with a short concavity in full-face view. Antennal scape same as minor but short, just reaching the occipital border (SL/CS: 0.77±0.04, 0.74–0.83). Promesonotum forms a unique dome followed by an impressed mesonotal suture, dorsum of propodeum slightly concave near the propodeal angle. Anterior portion of head with widely spaced large punctures, a single puncture in the center of the vertex. Filiform brownish hair present on head and mesosoma dorsum, more abundant on malar area.

##### Distribution and biology.

*Camponotuschrislaini* is restricted to the northern portion of Madagascar (Fig. 70B). This newly identified species prefers dry (tropical dry forest, dry forest, disturbed dry forest) as well as wet (montane forest and rainforest) habitats at moderate altitudes (below 1000 m). It was primarily collected from rotten logs, sifted litter, inside trees, or on branches above ground.

##### Discussion.

The new species is easily distinguishable from the *Mayria* species, but morphologically is most similar to the unidentified species Camponotus MG098 from subgenus Myrmonesites. The main character used to distinguish them is the form of the petiolar scale, as in the former the petiole is much thinner and its dorsal face forms a straight surface, while in the latter the petiole is thickened with the posterior face concave in dorsal view. *Camponotus* MG098 is also covered with plentiful setae which are scarce and widely distributed in *C.chrislaini*. The presence of *C.chrislaini* and *C.* MG098 in six localities (Sakaramy, Binara, Antsahabe, Analamerana-Ankavanana, Galoko, and Mont Kalabenono) suggests that they are reproductively isolated.

##### Additional material examined.

**Province Antsiranana**: Forêt de Binara, 7.5 km 230° SW Daraina, –13.255, 49.61667, 375 m, tropical dry forest (B.L. Fisher) (CAS); Forêt d’ Antsahabe, 11.4 km 275° W Daraina, –13.21167, 49.55667, 550 m, tropical dry forest (B.L. Fisher) (CAS); Forêt d’ Andavakoera, 21.4 km 75° ENE Ambilobe; 4.6 km 356° N Betsiaka, –13.11833, 49.23, 425 m, rainforest (B.L. Fisher) (CAS); Res. Analamerana, 16.7 km 123° Anivorano-Nord, –12.80467, 49.37383, 225 m, tropical dry forest (B.L. Fisher) (CAS); Parc National Montagne d’Ambre, Fozalanana, –12.46637, 49.22157, 475m, dry forest (BLF) (CAS); Res. Ankarana, 7 km SE Matsaborimanga,–12.9, 49.11667, 150 m, tropical dry forest (PSW) (PSWC); Galoko chain, Mont Galoko, –13.58487, 48.71818, 520 m, rainforest (BLF) (CAS); Galoko chain, Mont Galoko, –13.5888, 48.72864, 980 m, montane forest (BLF) (CAS); Galoko chain, Mont Galoko, –13.58745, 48.71419, 38 m, rainforest (BLF) (CAS); Galoko chain, Mont Kalabenono, –13.63999, 48.67374, 498 m, rainforest (BLF) (CAS); Galoko chain, Mont Kalabenono, –13.64179, 48.67282, 643 m, rainforest (BLF) (CAS); Galoko chain, Mont Kalabenono, –13.64609, 48.67732, 937 m, rainforest (BLF) (CAS); Ampasindava, Andranomatavy Forest, –13.6648, 47.98702, 275 m, disturbed dry forest (BLF) (CAS); Reserve Spéciale d’Ambre, 3.5 km 235° SW Sakaramy, –12.46889, 49.24217, 325 m, tropical dry forest (FGAT) (CAS); Reserve Spéciale de l’Ankarana, 22.9 km 224° SW Anivorano Nord, –12.90889, 49.10983, 80 m, tropical dry forest (FGAT) (CAS); Ampasindava, Forêt d’Ambilanivy, 3.9 km 181° S Ambaliha, –13.79861, 48.16167, 600 m, rainforest (FGAT) (CAS).

**Figure 60.**
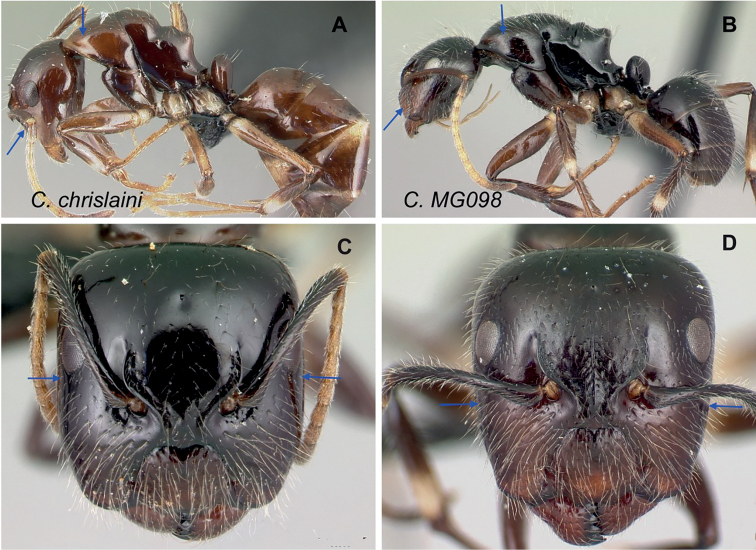
**A** minor worker of *C.chrislaini* (CASENT0498999) **B** minor worker of *C.* MG098 (CASENT0465407) **C** head of major worker of *C.chrislaini* (CASENT0499002) **D** head of major worker of *C.* MG098 (CASENT0465404).

### The *Camponotusellioti* species group

A description of *Camponotusmaintikibo* can be found in Rakotonirina et al. (2016): 229–231.

#### ﻿ Camponotus andrianjaka sp. nov.

Taxon classificationAnimaliaHymenopteraFormicidae

B07D7FC5-2507-52D4-8742-BC8001EFFC5C

http://zoobank.org/85932AC5-3790-4161-B72C-134BF27EAC0A

**Figure 61.**
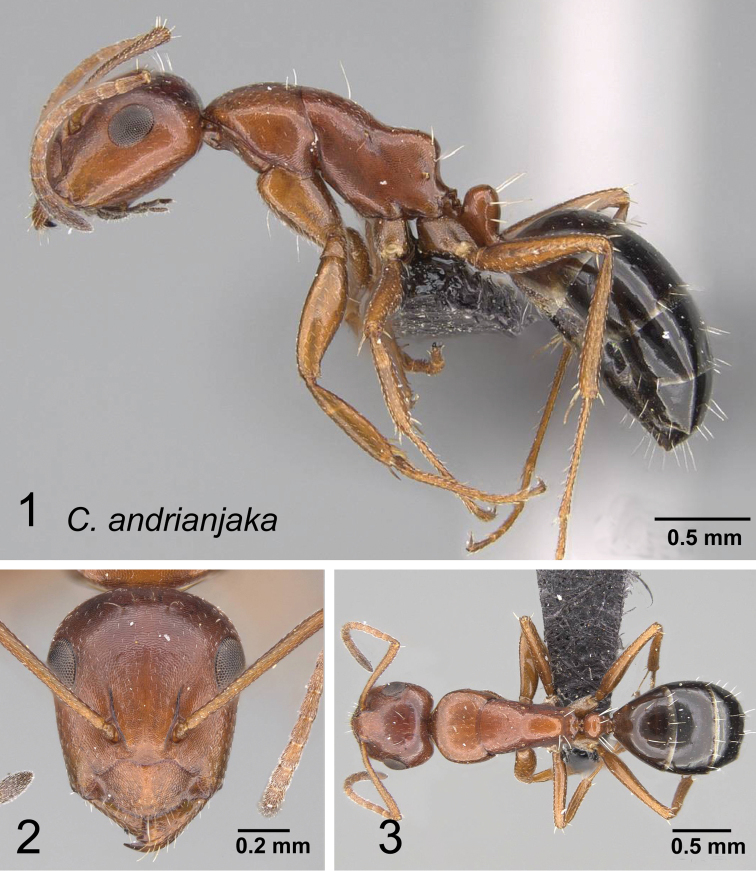
*Camponotusandrianjaka* minor worker (CASENT0243690) **1** body in lateral view **2** head in full-face view **3** body in dorsal view.

**Figure 62.**
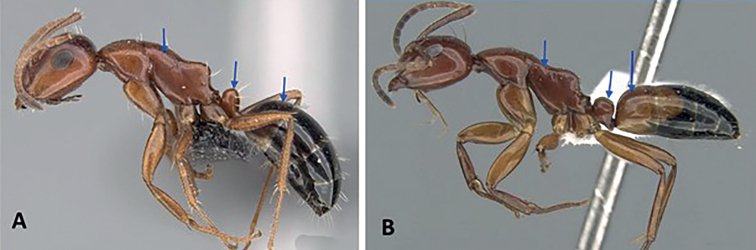
A minor worker of *C.andrianjaka* (CASENT0243690) **B** minor worker of *C.* MG132 (CASENT0188254).

Figures 8D , 39B , 61A–C , 62

##### Holotype worker.

Madagascar, Province Fianarantsoa, Isalo III National Parc, 12.02 km SW Ranohira, –22.6158, 45.31084, 870 m, Bismarckia woodland, ex soil, 15–24 February 2010 (ARA), collection code: ARA0306, specimen code: CASENT0243690 (CAS). **Paratype.** one worker with same data as holotype but specimen coded as CASENT0243954 (CAS).

##### Worker diagnosis.

*Camponotusandrianjaka* can be easily distinguished from other species of the *ellioti* group by the following character set: body bicolored, head and mesosoma reddish brown, gaster dark brown to black; anterior clypeal margin rounded, not produced; propodeum dorsum slightly concave at the level of metanotal suture; petiole nodiform and surmounted with standing, whitish hairs on its posterior margin.

##### Description of minor worker.

Medium-sized species. Absolute cephalic size (CS: 0.84±0.07; 0.81–0.88). In full-face view, head subovate, usually somewhat longer than broad, posterior cephalic margin roundly convex; lateral margin of head straight and tapering to front (CWb/CL: 0.31±0.01, 0.30–0.32). Eyes subelliptical, protruding, its border smoothly aligned to lateral margins of head (PoOC/CL: 0.08±0.01, 0.08–0.09). Mandibles triangular with six teeth. Clypeus carinate, anterior clypeal margin with rounded triangular projection (ClyL/GPD: 0.24±0.01, 0.23–0.25). Antennal scape long, surpassing the occiput by the length of two basal funiculi (SL/CS: 0.36±0.04, 0.35–0.38). In lateral view, mesosoma without anterolateral margination, promesonotum convex, mesopropodeal suture shallowly impressed, propodeum rather angulate with slightly concave basal and declivitous surface (MW/ML: 0.19±0.02, 0.18–0.21, MPH/ML: 0.15±0.01, 0.14–0.15). In lateral view, petiole nodiform, higher than long, its short anterior face rounding gradually to the convex posterior faces. Head, especially anterior portion, finely reticulate-areolate, gaster and mesosoma finely imbricate. Mandibles finely reticulate-punctate. Hairs whitish, one pair present on vertex, middle of mesonotum, and propodeum dorsum; three to four pairs on each side of lateral corner of propodeum; four pairs transversely arranged on petiolar posteromargin; ground pubescence delicate. Head and mesosoma reddish, legs light brown, basal face of first gastral segment light brown and the remainder of the segment dark brown to black.

##### Description of major worker.

Characteristics of minor workers, except: head as long as broad, with rather straight posterior and lateral borders (CS: 1.15±0.05, 1.12–1.20, CWb/CL: 0.84±0.01, 0.84–0.85). Eyes circular, placed dorsally next to lateral borders (PoOC/CL: 0.26±0.03, 0.23–0.27). Anterior clypeal margin forms a rounded rectangular lobe (ClyL/GPD: 0.89±0.05, 0.83–0.92). Antennal scape short, just reaching the occipital border (SL/CS: 0.70±0.02, 0.69–0.72). Mesosoma the same as minor worker.

##### Distribution and biology.

*Camponotusandrianjaka* is a very distinctive species that appears endemic to *Bismarckia* woodland, shrubland, Uapaca woodland, and savannah woodland in the southern part of Madagascar (Fig. 70D). It can be found also on eucalyptus plantations and grassland. Altitude: 1300–1987 m.

##### Discussion.

At first sight, *Camponotusandrianjaka* may be confused with another new species, Camponotus MG132, subgenus Myrmonesites, but can be separated easily with mesonotum that is gradually rounded in dorsal view; petiole higher than long, its dorsolateral margin surmounted with white hairs; presence of ground pubescence; and gaster all black. In addition, *C.* MG132 has been collected from rainforest and montane rainforest and absent from woodland.

##### Etymology.

This new species is named after the collector Andrianjaka Ravelomanana.

##### Additional material examined.

**Province Fianarantsoa**: Ampandravelo III Non Protected Area, 10.72 km NE Ranohira, –22.53944, 45.51497, 869 m, shrubland (ARA) (CAS); Ampangabe III Non Protected Area, 21.26 km W Itremo, –20.6125, 46.60883, 1412 m, savannah woodland (ARA) (CAS); Ampangabe VI Non Protected Area, 21.16 km W Itremo, –20.61444, 46.6104, 1379 m, shrubland (ARA) (CAS); Ampangabe VII Non Protected Area, 21.2 km W Itremo, –20.61417, 46.60989, 1420 m, shrubland (ARA) (CAS); Ampotoampoto II National Parc, 8.07 km NW Ilakaka,–22.62806, 45.18843, 919 m, savannah woodland (ARA) (ATOL UCDC); Ampotoampoto III National Parc, 7.91 km NW Ilakaka, –22.62944, 45.189, 919 m, savannah woodland (ARA) (ATOL voucher CAS); Antohatsahomby III Non Protected Area, 22.79 km NW Itremo, –20.54806, 46.58599, 1499 m, Uapaca woodland (ARA) (CAS); Antohatsahomby V Non Protected Area, 22.63 km NW Itremo, –20.56722, 46.57923, 1726 m, Uapaca woodland (ARA) (CAS); Isalo III National Parc, 12.02 km SW Ranohira, –22.61583, 45.31084, 870 m, Bismarckia woodland (ARA) (CAS); Isalo IV National Parc, 12 km SW Ranohira, –22.61472, 45.31304, 867 m, Bismarckia woodland (ARA) (ATOL USNM); Isalo IV National Parc, 12 km SW Ranohira, –22.61472, 45.31304, 867 m, Bismarckia woodland (ARA) (CAS); Mampiarika IV Non Protected Area, 27.98 km SW Ambositra, –20.73528, 47.08382, 1486 m, Uapaca woodland (ARA) (ATOL CAS).

#### ﻿ Camponotus ellioti

Taxon classificationAnimaliaHymenopteraFormicidae

Forel

56A450F4-8CB9-5F46-8775-CE68A76E8CDF

**Figure 63.**
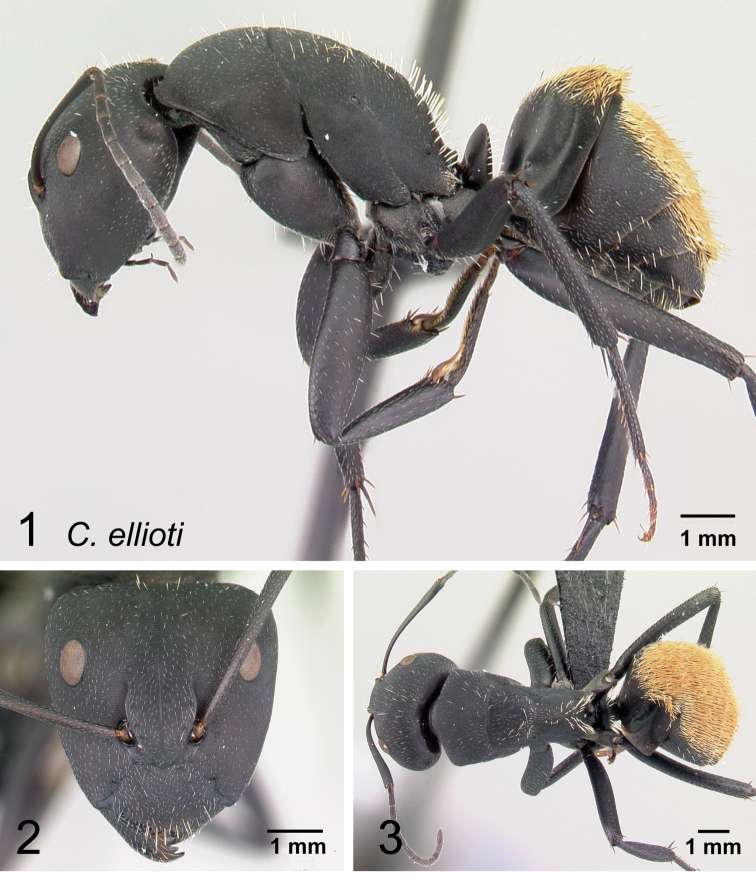
*Camponotusellioti* minor worker (CASENT0450893) **1** body in lateral view **2** head in full-face view **3** body in dorsal view.

Figures 2A , 8A , 38B , 63

Camponotus ellioti Forel, 1891: 37, 73. Holotype minor worker, Madagascar, collection Henri de Saussure. AntWeb CASENT0101382 (MHNG) (examined).
Camponotus (Myrmepomis) ellioti Forel, 1912: 92; Emery, 1920: 258.
Camponotus (Myrmopiromis) ellioti Wheeler, 1922: 1052; Emery, 1925: 128; Bolton, 1995: 97, 131.
Camponotus ellioti var. relucens Santschi, 1911: 133. Lectotype two workers, present designation, Madagascar, Toliara Province, Region du Sud-Est Fort-Dauphin (Ch. Alluaudi), AntWeb CASENT0101196 (minor); CASENT0101197 (major) (NHMB).
Camponotus (Myrmopiromis) ellioti var. relucens Wheeler, 1922: 1052; Emery, 1925: 129; Bolton, 1995: 120. Syn. nov.

##### Diagnosis.

Head and mesosoma reddish brown to dark brown, gaster always dark colored; anterior clypeal margin of clypeus with short, rectangular lobe; pronotal humeri tuberculate, dorsal outline of mesosoma arcuate; gastral tergite with short, decumbent, ochreous setae.

##### Description of minor worker.

Large-sized species. Absolute cephalic size (CS: 1.85±0.55, 1.45–2.42). In full-face view, head trapezoidal, wider than length of pronotum in dorsal view; occipital margin slightly convex medially, and straight subparallel lateral margin of head (CWb/CL: 0.36±0.03, 0.33–0.39). Eyes elliptical, positioned at the midline of head (PoOC/CL: 0.08±0.01, 0.07–0.09). Mandibles short and triangular with six teeth. Clypeus trapeziform, less convex in profile, sharply carinate along its entire length, with a short, rectangular projection feebly sinuate in the middle (ClyL/GPD: 0.12±0.01, 0.11–0.13). Frontal carina less sinuate and divergent. Antennal scape long and surpassing the occipital corner (SL/CS:0.41±0.07;0.34–0.46). In lateral view, dorsal outline of mesosoma forms a smooth curve, anterodorsal corner of pronotum distinctly shouldered, propodeum rounded, its surface convex and not marginate laterally, declivitous face triangular in posterior view (MW/ML: 0.23±0.03, 0.21–0.25; MPH/ML: 0.18±0.03, 0.16–0.20). Petiolar node squamiform in profile, wider than long, anterodorsal face distinctly flat and inclined forward, posterodorsal face flat, node summit arcuate. First abdominal tergite with vertical anterior face. Entire body (frontal area, dorsal face of mesosoma, scape, and legs) entirely, finely, and densely reticulate-punctate and matte; lateral face of petiole and abdomen transversally striolate. Mandible finely reticulate-punctate and subopaque, with superimposed, abundant punctuation arranged transversally. Ground pilosity yellowish, short, sparse, and dilute on tibia and scape. Short white to yellowish standing hair present on entire dorsum, sparsely distributed on occipital region, regularly present on petiolar dorsum and onto declivity, absent on scape and tibia. Dorsum of gastral segment with appressed ochreous setae and whitish, suberect hairs arranged serially parallel and longitudinally. Head and mesosoma dark brown to reddish brown; mandible, basal half of scape, basitarsus, and gastral segment reddish.

##### Description of major worker.

Characteristics of minor workers, except: head as long as broad, with rather straight posterior and lateral borders (CS: 4.13±0.16, 3.90–4.33; CWb/CL: 1.04±0.02, 1.01–1.07). Eyes circular, placed dorsally next to lateral borders (PoOC/CL: 0.28±0.01, 0.26–0.29). Anterior clypeal margin forms a rectangular lobe (ClyL/GPD: 0.87±0.05, 0.79–0.92). Antennal scape short, not reaching the occipital border (SL/CS: 0.61±0.02, 0.58–0.65). Promesonotal suture strongly marked.

##### Distribution and biology.

*Camponotusellioti* occurs in the dry forest regions of the southeast of Madagascar in habitats such as bush, coastal scrub, and coastal spiny bush on sandy soil, *Euphorbia* forest, spiny bush and thicket, gallery forest, and riparian scrub (Fig. 70C). It has been collected from sifted litter and Malaise traps. The altitudes of these localities range from 5 to 230 meters.

##### Discussion.

*Camponotusellioti* is a distinctive species recognizable by the submargined anterolateral corner of the pronotum and the gaster covering of thick, blunt hairs the color of rust. In the traditional classification, *C.ellioti* is considered a member of the *C.darwinii* species group based on the density and color of the gastral pilosity and the sculpture pattern; however, we propose color as a character difference between these two groups.

##### Additional material examined.

**Province Toliara**: [Région du Sud Andrahomana, Museum Paris, Ch. Alluaud 1901]; Analapatsa I; Taolagnaro, –25.1833333, 46.6666667, 31 m, (NHMB); [Toliara S.W. Madagascar]; Beronono; Mahabo, –21, 45, 339 m (Voeltzkow) (MHNG); 3.5km 236° SW Marovato, –25.55389, 45.25583, 230 m, spiny forest/thicket (FGAT) (CAS); 5 km N Ampotaka, Malaise on trail in Vitambany gallery forest, –24.65033, 43.96317, 86 m, gallery forest (MG) (CAS); 7.0 km 156° SSE Lavanono, –25.47111, 44.9885, 50 m, spiny forest/thicket (FGAT) (CAS); Ambohimahavelona village 33 km NE of Toliara, Andoharano, dry forest, –23.44083, 43.89967, 46 m, dry forest (MG) (CAS); Andranovato, 5 km SE of Manombo, –22.81806, 43.50217, 18 m, Euphorbia forest (CAS); Androy Region, District of Tsihombe, 74 km S of Tsihombe, Cap Sainte Marie Reserve, –25.58767, 45.163, 36 m, spiny bush (MG) (CAS); Anosy Region, District of Amboasary, 58 km SW of Fort Dauphin, 8 km NW of Amboasary, Berenty Special Reserve, –25.00667, 46.30333, 85 m, gallery forest (MG) (CAS); Atsimo-Andrefana Region, Antsokay Arboretum, –23.41491, 43.75499, 13 m, spiny forest/thicket (B.L. Fisher, F.A. Esteves et al.) (CAS); Atsimo-Andrefana Region, Famata 02, –23.45314, 43.76448, 20 m, coastal spiny bush on sandy soil (B.L. Fisher, F.A. Esteves et al.) (CAS); Atsimo-Andrefana Region, Famata04, –23.53922, 43.77935, 20 m, riparian scrub (B.L. Fisher, F.A. Esteves et al.) (CAS); Forêt de Petriky, 12.5 km W 272° Tolagnaro, –25.06167, 46.87, 10 m, littoral rainforest (B.L. Fisher) (CAS); Libanona beach, Tolagnaro, –25.03883, 46.996, 20 m, coastal scrub (BLF) (CAS); Mahafaly Plateau, 6.2 km 74° ENE Itampolo, –24.65361, 43.99667, 80 m, spiny forest/thicket (FGAT) (CAS); Mikea Forest, spiny forest, –22.91333, 43.48222, 37 m, spiny forest (R. Harin’Hala) (CAS); Parc National de Tsimanampetsotsa, 6.7 km 130° SE Efoetse, 23.0 km 175° S Beheloka, –24.10056, 43.76, 25 m, spiny forest/thicket (FishFGAT) (CAS); Parc National de Tsimanampetsotsa, Forêt de Bemanateza, 20.7 km 81° E Efoetse, 23.0 km 131° SE Beheloka, –23.99222, 43.88067, 90 m, spiny forest/thicket (FiFGAT) (CAS); Ranobe, –23.04457, 43.61532, 20 m, spiny forest/thicket (Frontier Wilderness Project) (CAS); Réserve Privé Berenty, Forêt d’Anjapolo, 21.4 km 325° NW Amboasary, –24.92972, 46.20967, 65 m, spiny forest/thicket (FGAT) (CAS); Réserve Spéciale de Cap Sainte Marie, 12.3 km 262° W Marovato, –25.58167, 45.16833, 200 m, spiny forest/thicket (FGAT) (CAS); Réserve Spéciale de Cap Sainte Marie, 14.9 km 261° W Marovato, –25.59444, 45.14683, 160 m, spiny forest/thicket (FGAT) (CAS); Tolagnaro (= Fort Dauphin), –25.03333, 47, 5 m, littoral vegetation (B.L. Fisher) (CAS); Tolagnaro (= Ft. Dauphin), –25.04611, 47, 30 m, garden, hotel (D.M. Olson) (PSWC); Toliara, –23.3575, 43.669, 20 m, urban/garden (BLF) (CAS); Tsimanampetsotsa National Park, Mitoho Forest, Malaise across trail at escarpment base, –24.0485, 43.75233, 120 m, dense dry forest and transitional forest (MG) (CAS); Tsimelahy – Parcel II, Andohahela National Park, transitional forest, Toliara Province, –24.93683, 46.62667, 180 m, transition forest (M.E. Irwin, F.D. Parker, R. Harin’Hala) (CAS).

#### ﻿ Camponotus maintilany sp. nov.

Taxon classificationAnimaliaHymenopteraFormicidae

45336E95-77B3-51B3-8C64-8D581ABF175A

http://zoobank.org/E90CD454-E16D-45AF-9F60-57F6F7343A1C

**Figure 64.**
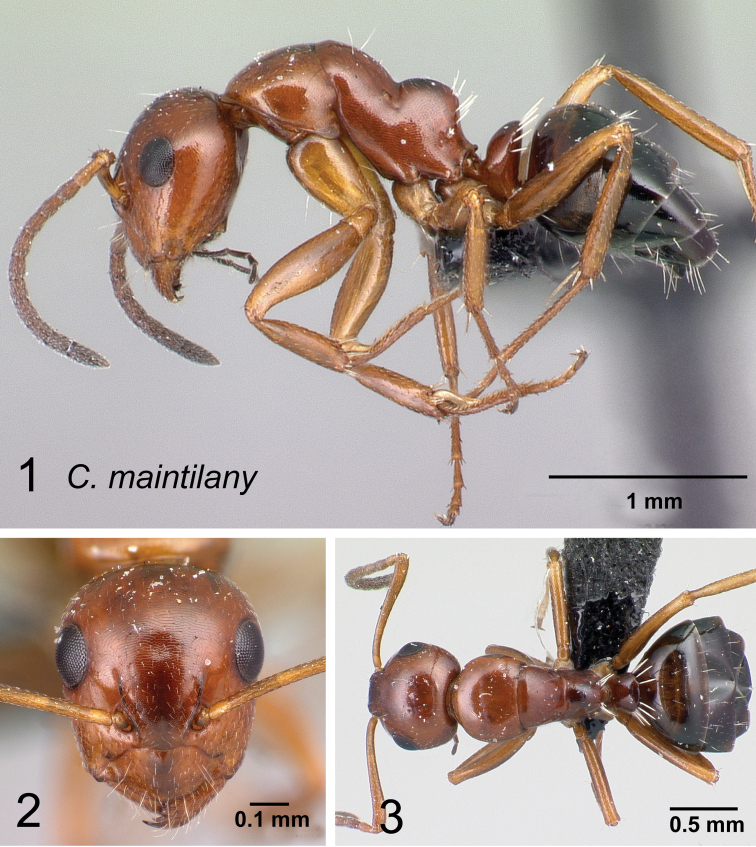
*Camponotusmaintilany* minor worker (CASENT0120678) **1** body in lateral view **2** head in full-face view **3** body in dorsal view.

Figures 37B , 39A , 64

##### Holotype worker.

Madagascar, Province Antananarivo, Ankalalahana, –19.00659, 47.1122, 1375 m, Uapaca woodland, ground forager(s), 29 March 2011 (BLF), collection code: BLF26368, specimen code: CASENT0245156 (CAS). **Paratypes.** Two workers with same data as holotype but collection code: BLF26364, specimen code: CASENT0245172; collection code: BLF26385, specimen code: CASENT0245187 (CAS).

##### Worker diagnosis.

Ants bicolored: head and mesosoma reddish brown, gaster dark brown to black; pronotum and mesonotum form a unique dome followed by an impressed metanotal suture, propodeum, and petiole angle with standing, whitish, spatulate hairs, head and gastral dorsum with sparse, fine, brown hairs; petiole squamiform.

##### Description of minor worker.

Medium-sized species. Absolute cephalic size (CS: 0.84±0.05, 0.81–0.86). In full-face view, head distinctly longer than broad, with convex occipital margin and subparallel lateral sides (CWb/CL: 0.33±0.02, 0.32–0.34). Eyes elliptical and break the lateral outlines of head (PoOC/CL: 0.09±0.01, 0.08–0.10). Mandibles triangular with six short, blunt teeth. Clypeus convex, its anterior border produced into rounded lobe (ClyL/GPD: 0.24±0.01, 0.22–0.25). Antennal scape long, surpassing the occipital margin (SL/CS: 0.40±0.03, 0.39–0.43). In lateral view, mesosoma without anterolateral margination, promesonotum distinctly convex, mesopropodeal suture deeply impressed so that propodeum dorsum is raised to the level of mesopropodeal suture, propodeum angular in profile, with unequal basal and declivitous faces, the former horizontal, the latter straight to evenly concave (MW/ML: 0.20±0.01, 0.19–0.20; MPH/ML: 0.14±0.03, 0.12–0.15). In lateral view, petiole cuneate, its anterior face convex, its posterior face more flattened with entire border broad. Body finely imbricate. Hairs grayish, present only on vertex, one pair next to the pronotal suture, one pair on the middle of mesonotum dorsum, sparse on gastral tergite; enlarged, whitish hairs present on propodeal corner (two or three pairs) and on posteromargin of petiole; pubescence whitish and more dilute on gaster tergites. Head, mesosoma, and appendages reddish to brownish yellow; first gastral segment lighter colored than the remaining dark brown segments.

##### Description of major worker.

Characteristics of minor workers, except: head apparently rectangular, with straight to feebly convex posterior margin and parallel lateral sides. (CS: 1.13±0.05, 1.09–1.18; CWb/CL: 0.90±0.02, 0.88–0.91). Eyes circular, placed dorsally next to the lateral margin (PoOC/CL: 0.26±0.01, 0.25–0.27). Anterior clypeal margin forms a short, rounded lobe (ClyL/GPD: 0.83± 0.00, 0.83–0.83). Antennal scape short, just reaching the occipital border (SL/CS: 0.77±0.03, 0.74–0.79). Dorsal outline of mesosoma not very convex and interrupted but pronotal and mesopropodeal suture present, the latter weakly impressed.

##### Distribution and biology.

*Camponotusmaintilany* occurs from 1300–1987 m in elevation and inhabits a montane environment in the central highlands, where it prefers mostly Uapaca forest or woodland, but is also found in savanna grassland and eucalyptus plantations (Fig. 70E). This new species has been collected by digging in soil, sifting leaf litter, and baiting with tuna fish or sardines. In addition, a few workers were found in Malaise traps.

##### Discussion.

*Camponotusmaintilany* is most readily distinguished from other members of the *ellioti* species group by a deeper metanotal groove and shinier sculpture. It may be confused with *C.andrianjaka* but can be separated by its impressed metanotal suture and truncate petiolar node.

##### Etymology.

This species is derived from the coloration of the mesosoma and gaster. “Mainty” means black and “ilany” means second part.

##### Additional material examined.

**Province Antananarivo**: Alasora, –18.96245, 47.58925, 1434 m, grassland (BLF) (CAS); Ambatolaona, –18.928, 47.88283, 1382 m, urban/garden (BLF) (CAS); Ambohidrabiby, –18.7746, 47.60908, 1362 m, eucalyptus plantation (BLF) (CAS); Analamanga Region, District of Arivonimamo, Andebadeba Uapaca forest, 4 km from Arivonimamo, –19.00806, 47.14861, 1300 m, Uapaca forest (MG) (CAS); Andohony I Non Protected Area, 22.62 km SW Antsirabe, –20.06784, 46.99068, 1451 m, Savannah grassland (ARA) (CAS); Andohony II Non Protected Area, 22.71 km SW Antsirabe, –20.06904, 46.99199, 1398 m, savanna grassland (ARA) (CAS); Ankalalahana, –19.00711, 47.1337, 1350 m, Uapaca woodland (BLF) (CAS); Ankalalahana, –19.00659, 47.1122, 1375 m, Uapaca woodland (BLF) Ankalalahana, –19.00659, 47.1122, 1375 m, Uapaca woodland (BLF) (CAS); Ankalalahana, –19.00716, 47.1124, 1370 m, Uapaca woodland (BLF) (CAS); Ankalalahana, –19.0531, 47.11304, 1314 m, Uapaca woodland (H.E. Marti et al.) (CAS); Ankalalahana, –18.99666, 47.1118, 1353 m, Uapaca woodland (H.E. Marti et al.) (CAS); Antaponimanadala III Non Protected Area, 6.55 km E Manalalondo, –19.25583, 47.17751, 1987 m, savanna grassland (ARA) (CAS); Iharanandriana, –19.15823, 47.49702, 1513 m, Uapaca woodland (BLF) (CAS), Kaloy, –18.59568, 47.65333, 1338 m, grassland (BLF) (CAS); Navoatra I Non Protected Area, 7.64 km NW Arivonimamo, –18.97806, 47.11929, 1373 m, Uapaca woodland (ARA) (CAS); Navoatra III Non Protected Area, 7.39 km NW Arivonimamo, –18.98028, 47.12071, 1321 m, Uapaca woodland (ARA) (CAS); Navoatra IV Non Protected Area, 7.15 km NW Arivonimamo, –18.98194, 47.12232, 1323 m, Uapaca woodland (ARA) (CAS).

### The *Camponotusmadagascarensis* species group

Descriptions of the four other species within *madagascarensis* group are in Rakotonirina et al. (2017): *Camponotusdescarpentriesi* Santschi, 1926 on pp. 231–233, *Camponotusmadagascarensis* Forel, 1886 stat. rev. on pp. 233–236, *Camponotusmita* Rakotonirina, Csősz & Fisher, 2017 on pp. 237–238, and *Camponotusvoeltzkowii* Forel, 1894 on pp. 239–243.

#### ﻿ Camponotus ivadia sp. nov.

Taxon classificationAnimaliaHymenopteraFormicidae

8AE00EA9-3EBC-587A-BDC2-AB6DE530B564

http://zoobank.org/840E714E-B70B-4EA1-9BBD-B8A19FCCFDCE

**Figure 65.**
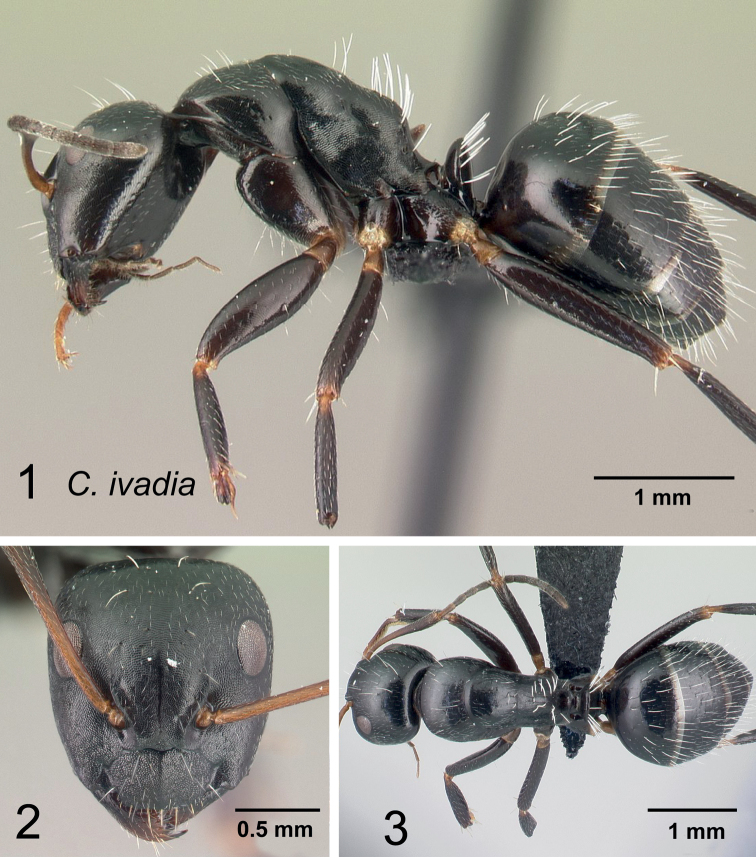
*Camponotusivadia* minor worker (CASENT0498634) **1** body in lateral view **2** head in full-face view **3** body in dorsal view.

Figures 9D , 50C, D , 65

##### Holotype worker.

Madagascar, Province Antsiranana, Forêt d’ Ampondrabe, 26.3 km 10° NNE Daraina, tropical dry forest, on low vegetation, –12.97, 49.7, 175 m, 04 November 2009 (BLF) collection code: BLF10105, specimen code: CASENT0498634 (CAS). **Paratype.** One worker with same data as holotype but specimen coded as CASENT0498635 (CAS).

##### Worker diagnosis.

Integument dark brown, apparently shiny; hairs on mesosoma and petiole blunt-tipped; fine, pointed hairs on head and gastral dorsum; anterior clypeal margin with straight rectangular lobe; dorsal outline of mesosoma nearly evenly arcuate; petiole higher than long.

##### Description of minor worker.

Medium-sized species. Absolute cephalic size (CS: 1.21±0.19, 1.13–1.30). In full-face view, head almost quadrate, with feebly convex posterior and lateral border (CWb/CL: 0.35±0.03, 0.33–0.36). Eyes elliptical, placed next to the lateral margins of head (PoOC/CL: 0.10±0.01, 0.10–0.11). Mandibles triangular with six teeth. Anterior clypeal margin angularly produced, with straight lateral border and rounded anterolateral corner. Antennal scape long, surpassing the occipital corner by the length of two basal funiculi (SL/CS: 0.39±0.02, 0.38–0.40). In lateral view, pronotum with short anterolateral margination, mesosoma moderately convex, propodeum angular in profile, with unequal basal and declivitous faces, each straight in profile and meeting at a rounded obtuse angle (MW/ML: 0.21±0.03, 0.19–0.22; MPH/ML: 0.16±0.01, 0.16–0.17). In lateral view, petiole rather narrow, its anterior face convex and its posterior face flattened. Body finely imbricate. Hairs whitish; enlarged, whitish hairs with blunt tip on mesonotum, propodeum, and petiolar dorsum; filiform, pointed, grayish hair on head, pronotum, and gastral segment; pubescence white, short, and more abundant across entire dorsum. Head, mesosoma and gaster dark brown, femora, tibia, and funiculi brown, scape and basitarsi slightly lighter.

##### Description of major worker.

Characteristics of minor workers, except: head as long as wide, posterior borders medially concave and subparallel, lateral sides diverging apically (CS: 1.93; CWb/CL: 0.93). Eyes circular, placed dorsally in the middle of the lateral borders (PoOC/CL: 0.28). Anterior clypeal margin forms a short, rounded, rectangular lobe, slightly concave medially (ClyL/GPD: 0.84). Antennal scape short, just reaching the occipital border (SL/CS: 0.69). Sculpture, pilosity, and color as in minor worker.

##### Distribution and biology.

*Camponotusivadia* has thus far been collected only in the Forêt d’ Ampondrabe within Ankarana Reserve, a region with tropical dry forest located in the northern portion of Madagascar (Fig. 70F). All specimens were collected by beating vegetation.

##### Discussion.

This species is easily recognized within the *madagascarensis* group as one of few back and shiny species with white standing pubescence.

##### Etymology.

The species epithet ivadia is derived from the low vegetation microhabitat it prefers.

##### Additional material examined.

**Province Antsiranana**: Rés. Ankarana, 7 km SE Matsaborimanga, –12.9, 49.11667, 150 m, tropical dry forest (PSW) (PSWC); Forêt d’ Ampondrabe, 26.3 km 10° NNE Daraina, –12.97, 49.7, 175 m, tropical dry forest (B.L. Fisher) (CAS).

### The *Camponotusrepens* species group

#### ﻿ Camponotus jjacquia sp. nov.

Taxon classificationAnimaliaHymenopteraFormicidae

8F479C2D-6B09-58D4-845A-5D9316B32BA8

http://zoobank.org/7B513036-F4AC-45A4-8B70-5FCCAF2C534D

**Figure 66.**
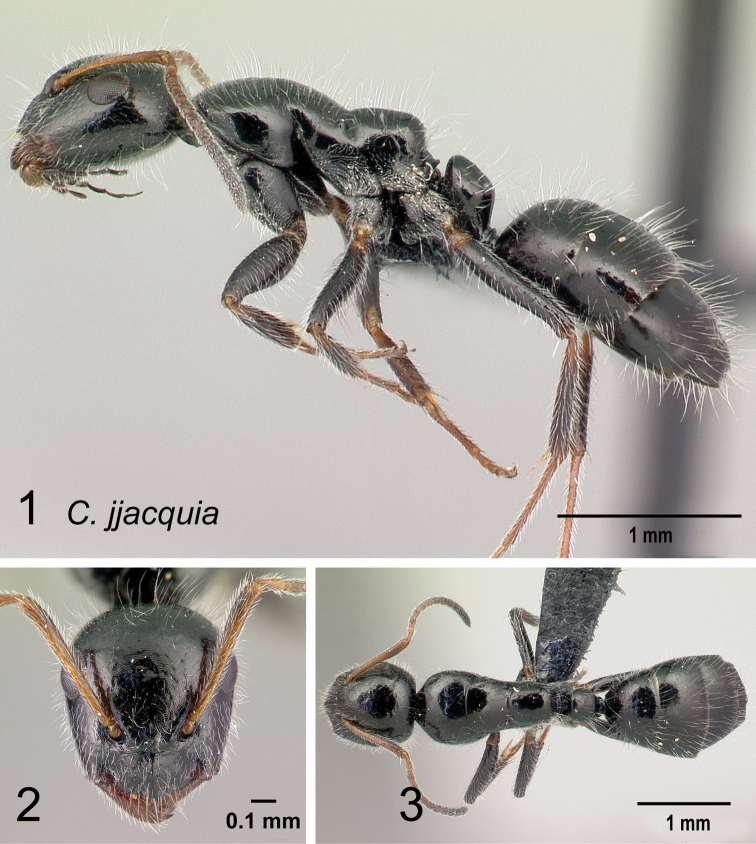
*Camponotusjjacquia* minor worker (CASENT0445276) **1** body in lateral view **2** head in full-face view **3** body in dorsal view.

Figures 10C, D , 45A , 66

##### Holotype worker.

Madagascar, Province Toliara, Parc National de Tsimanampetsotsa, Forêt de Bemanateza, 20.7 km 81°E Efoetse, 23.0 km 131°SE Beheloka, –23.99222, 43.88067, 90 m, spiny forest/thicket, under stone, 22–26 March 2002 (BLF), collection code: BLF06310, specimen code: CASENT0445276 (CAS). **Paratypes.** Four workers with the same data as holotype CASENT0445284, CASENT0445285, CASENT0445287, CASENT0445288 (CAS).

##### Worker diagnosis.

Integument black; entire body with plentiful, erect setae; anterior clypeal margin produced into a rounded triangular lobe; dorsal outline of mesosoma slightly impressed at the level of metanotum; petiole long and low.

##### Description of minor worker.

Medium-sized. Absolute cephalic size (CS: 0.99±0.14; 0.91–1.09). In full-face view, head almost quadrate (CWb/CL: 0.32 ± 0.02; 0.31–0.34); posterior margin evenly straight, lateral margin of head anterior to eyes slightly convex. Eyes circular, placed above midline of head (PoOC/CL: 0.10±0.02; 0.09–0.12). Mandibles triangular with six teeth. Clypeus carinate, its anterior border angularly produced in the middle (ClyL/GPD: 0.20±0.06, 0.16–0.23). Antennal scape long, surpassing the occiput by the length of one basal funiculus (SL/CS: 0.34±0.02; 0.32–0.34). In lateral view, anterodorsal corner of pronotum rounded, mesonotum dorsum inclined toward nearly straight propodeum face (MW/ML: 0.19±0.02, 0.18–0.20; MPH/ML: 0.13±0.02, 0.12–0.14). In lateral view, petiolar node longer than wide, with flat summit, its anterior face leaning forward. Ants finely foveolate throughout, apparently shiny. Entire body, including mandibles and appendages, covered with plentiful, erect, whitish setae, uneven in length, some long. Head, mesosoma, and gaster dark brown to black; antennae, mandibles, tibiae, and tarsi reddish brown.

##### Description of major worker.

Characteristics of minor workers, except: head much wider posteriorly (CS: 1.68±0.07, 1.61–1.76; CWb/CL: 0.91±0.02, 0.89–0.92); lateral margins convex and converging towards base of mandible. Eyes circular placed dorsolaterally midway from the occipital margin (PoOC/CL: 0.30±0.00, 0.29–0.30). Clypeus with a short rectangular lobe with rounded lateral angle (ClyL/GPD: 0.85±0.03, 0.81–0.88). Antennal scape short, not reaching the occipital margin (SL/CS: 0.57±0.02, 0.56–0.59). Dorsal outline of mesosoma interrupted by sutures, metanotal suture forms a canal. Head dorsum and mesosoma coarsely reticulate-punctate, petiole finely striolate, gaster imbricate and shiny.

##### Distribution and biology.

The known elevational range of *Camponotusjjacquia* extends from 43 to 923 m. Specimens have been collected on tree trunks, on low vegetation, in leaf litter, and under stones in *Bismarckia* woodland, deciduous forest, gallery forest, savannah woodland, spiny forest, and tropical dry forest (Fig. 72C). This species nests in the soil.

##### Discussion.

The unique petiolar shape, the density of its pilosity, and the shape of its metanotal dorsum make *C.jjacquia* a very distinctive species.

##### Etymology.

The new species is dedicated to Jean Jacques Rafanomezantsoa, who has been a Malagasy ant collector for more than twenty years.

##### Additional material examined.

**Province Fianarantsoa**: Ampotoampoto III National Park, 7.91 km NW Ilakaka, –22.62944, 45.189, 919 m, savannah woodland (ARA) (CAS); Isalo IV National Park, 12 km SW Ranohira, –22.61472, 45.31304, 867 m, Bismarckia woodland (ARA) (CAS); Ampotoampoto IV National Park, 7.83 km NW Ilakaka, –22.62944, 45.1912, 923 m, savanna woodland (ARA) (CAS); **Province Mahajanga**: Ampijoroa National Park, 160 km N Maevatanana, deciduous forest, –16.31944, 46.81333, 43 m, deciduous forest (MG) (CAS). **Province Toliara**: Anosy Region, District of Amboasary, 58 km SW of Fort Dauphin, 8 km NW of Amboasary, Berenty Special Reserve, –25.00667, 46.30333, 85 m, Galery forest (MG) (CAS); Anosy Region, District of Fort-Dauphin, Andohaela National Park Parcelle II, Tsimelahy, 42 km W of Fort-Dauphin, –24.93683, 46.62667, 176 m, transition forest (Mike Irwin, Frank Parker, Rin’ha) (CAS); Anosy Region, Parc National d’Andohahela, Forêt de Manatalinjo, –24.82505, 46.57811, 90 m, spiny forest/thicket (B.L. Fisher, F.A. Esteves et al.) (CAS); Atsimo Andrefana Region, District of Betioky; Beza Mahafaly Special reserve, Parcelle Belle vue 7 km W of Research Station, –23.68983, 44.5755, 177 m, spiny forest (Rin’ha) (CAS); Atsimo Andrefana Region, District of Betioky, 30 km E Betioky, Beza Mahafaly Special Reserve (around Research Station), –23.6865, 44.591, 165 m, gallery dry deciduous forest (MG); Beza-Mahafaly, 27 km E Betioky, –23.65,4 4.63333, 135 m, tropical dry forest (B.L. Fisher) (CAS); Forêt de Mahavelo, Isantoria River, –24.75833, 46.15717, 110 m, spiny forest/thicket (FGAT) (CAS); Parc National d’Andohahela, Forêt d’ Ambohibory, 1.7 km 61° ENE Tsimelahy, 36.1 km 308° NW Tolagnaro, –24.93, 46.6455, 300 m, tropical dry forest (FGAT) (CAS); Parc National d’Andohahela, Forêt de Manatalinjo, 33.6 km 63° ENE Amboasary, 7.6 km 99° E Hazofotsy, –24.81694, 46.61, 150 m, spiny forest/thicket (FGAT) (CAS); Parc National de Kirindy Mite, 16.3 km 127° SE Belo sur mer, –20.79528, 44.147, 80 m, tropical dry forest (FGAT) (CAS); Parc National de Tsimanampetsotsa, Forêt de Bemanateza, 20.7 km 81° E Efoetse, 23.0 km 131° SE Beheloka, –23.99222, 43.88067, 90 m, spiny forest/thicket (FGAT) (CAS); Beza Mahafaly Reserve, near research station, Parcel I, –23.6865, 44.591, 165 m, dry deciduous forest (R. Harin’Hala) (CAS); Beza Mahafaly Reserve, near Bellevue, Parcel II, –23.68983, 44.5755, 180 m, spiny forest (R. Harin’Hala) (CAS); Andohahela National Park, Tsimelahy – Parcel II, transitional forest, –24.93683, 46.62667, 180 m, transitional forest (M.E. Irwin, F.D. Parker, R. Harin’Hala) (CAS).

#### ﻿ Camponotus claveri sp. nov.

Taxon classificationAnimaliaHymenopteraFormicidae

200598EE-A3F4-5D8D-821B-ACDB7A0AB8D2

http://zoobank.org/1D76B9AA-E48D-4683-B19C-8D70CD063ADC

Figures 44B , 67

##### Holotype worker.

Madagascar, Province Fianarantsoa, Forêt d’ Atsirakambiaty, 7.6 km 285° WNW Itremo, –20.59333, 46.56333, 1550 m, montane rainforest, Malaise trap, 22–26 January 2003 (BLF), collection code: BLF07152, specimen code: CASENT0490618 (CAS). **Paratypes.** Three workers: one worker with same data as holotype but specimen collected from Malaise trap and with collection code: BLF07155, specimen code: CASENT0049849; two workers from Madagascar, Province Fianarantsoa, Mampiarika IV Non Protected Area, 27.98 km SW Ambositra, –20.73528, 47.08382, 1486 m, Uapaca woodland, 03–07 February 2010 (ARA), collection code: ARA0492, specimen code: CASENT0168788 and CASENT0168787 (CAS).

##### Worker diagnosis.

Integument shiny black; anterior clypeal margin with short, subtriangular lobe; erect hairs arranged in a transverse row on gastral segment, sparsely arranged on mesosoma dorsum; dorsum of mesosoma with promesonotal and mesometanotal sutures marked but not impressed; petiole squamiform.

##### Description of minor worker.

Medium-sized species. Absolute cephalic size (CS: 0.89±0.08, 0.84–0.93). In full-face view, head as long as wide, posterior margin rounded, lateral margin straight and subparallel (CWb/CL: 0.33±0.02, 0.32–0.34). Eye circular, protruding, its border smoothly aligned to lateral margins of head (PoOC/CL: 0.10±0.02, 0.08–0.10). Mandibles triangular with six teeth. Clypeus carinate, its anterior margin produced medially into a subtriangular lobe (ClyL/GPD: 0.23±0.07, 0.20–0.26). Antennal scape long, surpassing the occipital corner by the length of two-and-a-half basal funiculi (SL/CS: 0.39±0.03, 0.37–0.40). In lateral view, mesosoma without anterolateral margination, promesonotum not very convex, mesopropodeal suture impressed, propodeum angular with the base fully twice as long as the declivity, which is straight (MW/ML: 0.18±0.01, 0.18–0.19; MPH/ML: 0.14±0.01, 0.14–0.15). Petiole cuneate in profile, its anterior face convex, and its posterior face flattened along entire broad border. Body finely imbricate. Hairs whitish, one pair present on vertex, middle of mesonotum, and propodeum dorsum; two pairs on each side of lateral corner of propodeum; three pairs on lateral margins on petiole. Head and mesosoma reddish, legs light brown, basal face of first gastral segment light brown and the remaining segment dark brown to black.

**Figure 67.**
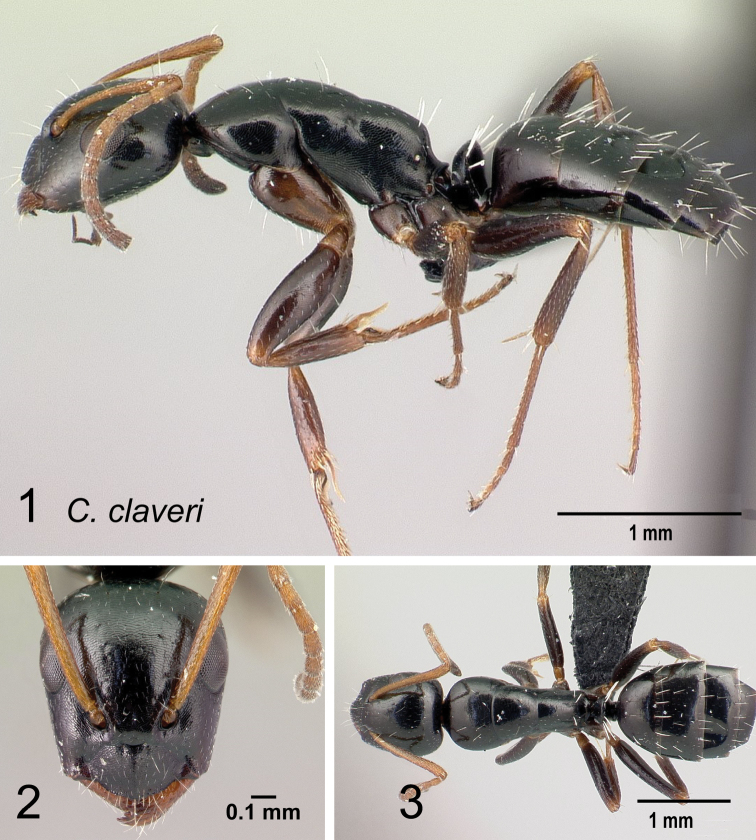
*Camponotusclaveri* minor worker (CASENT0490618) **1** body in lateral view **2** head in full-face view **3** body in dorsal view.

##### Description of major worker.

Characteristics of minor workers, except: head as long as broad, with rather convex posterior borders and subparallel lateral sides (CS: 1.18±0.09, 1.12–1.24, CWb/CL: 0.86±0.01, 0.85–0.87). Eyes circular, placed dorsally next to lateral borders (PoOC/CL: 0.26±0.00, 0.25–0.26). Anterior clypeal margin forms a rounded rectangular lobe (ClyL/GPD: 0.88±0.05, 0.85–0.91). Antennal scape short, just reaching the occipital border (SL/CS: 0.75±0.04, 0.73–0.78). Mesosoma with the same form as minor worker. Sculpture on head coarsely reticulate-punctate anteriorly and tends to be striolate posteriorly.

##### Distribution and biology.

*Camponotusclaveri* occupies two different habitats: montane rainforest and open habitats such as Uapaca woodland, eucalyptus plantation, shrubland, and deciduous dry forest, plus urban areas (Fig. 72A). Their nests are found in soil, under stones, and on the ground. It has been collected at elevations of 830–1987 m.

##### Discussion.

*Camponotusclaveri* is similar to other species of the *repens* group, but differs in having a thinner petiolar node with sharp dorsal edge, and a rounded triangular anterior clypeal margin. Morphometric analysis combined *C.claveri* and *C.maintilany* in the same cluster, meaning that they are the same size but have different qualitative morphological traits. Their distributions are quite distant but sympatric at Alasora, situated in the central highlands.

##### Etymology.

This species is named after the collector Claver Marotafika Randrianandrasana.

##### Additional material examined.

**Province Antananarivo**: Alasora, –18.96245, 47.58925, 1434 m, eucalyptus plantation (BLF) (CAS); Antaponimanadala III Non Protected Area, 6.55 km E Manalalondo, –19.25583, 47.17751, 1987 m, savanna grassland (ARA) (CAS); Ilafy, –18.85415, 47.56575, 1385 m, urban/garden (BLF) (CAS). **Province Fianarantsoa**: Amoron’i Mania Region, District of Ambositra, Italaviana Uapaca forest, 135 km SE of Antsirabe, –20.17333, 47.086, 1359 m, Uapaca forest (MG) (CAS); Ampandravelo II Non Protected Area, 10.78 km NE Ranohira, –22.53917, 45.51548, 87 3m, shrubland (ARA) (CAS); Antapia III Non Protected Area, 26.43 km SW Ambositra, –20.72, 47.08785, 1494 m, Uapaca woodland (ARA) (CAS); Forêt d’ Atsirakambiaty, 7.6 km 285° WNW Itremo, –20.59333, 46.56333, 1550 m, montane rainforest (FGAT) (CAS); Mampiarika III Non Protected Area, 28.93 km SW Ambositra, –20.73583, 47.08399, 1487 m, Uapaca woodland (ARA) (CAS); Mampiarika IV Non Protected Area, 27.98 km SW Ambositra, –20.73528, 47.08382, 1486 m, Uapaca woodland (ARA) (CAS).

#### ﻿ Camponotus ihazofotsy sp. nov.

Taxon classificationAnimaliaHymenopteraFormicidae

620DC68E-0ECC-53C5-BE04-D2935A560375

http://zoobank.org/72505F05-9AE7-4072-9341-EE9BD4298E8F

Figures 46B , 68

##### Holotype worker.

Madagascar, Province Toliara, Ihazofotsy – Parcel III, Andohahela National Park, –24.83483, 46.48683, 80 m, tropical dry forest, transition between spiny and dry deciduous forests, Malaise traps, 26 January– 03 March 2003 (M.E. Irwin, F.D. Parker, R. Harin’Hala), collection code: MA–02–21–08, specimen code: CASENT0062675 (CAS). **Paratypes.** Two workers with same data as holotype but collection code: MA–02–21–01, specimen codes: CASENT0062267 and CASENT0062269 (CAS)

**Figure 68.**
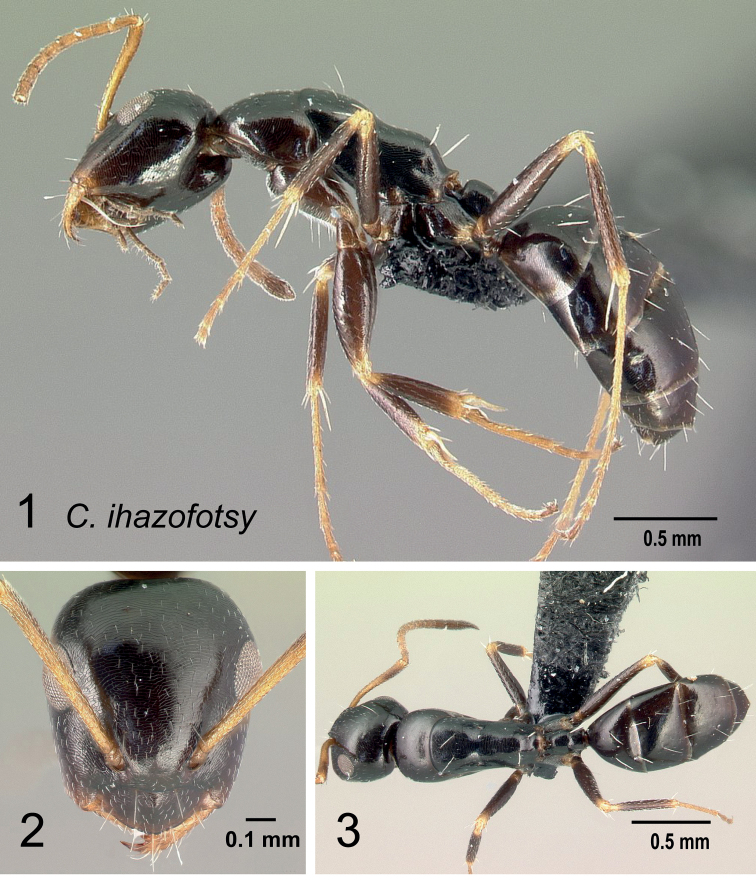
*Camponotusihazofotsy* minor worker (CASENT0062675) **1** body in lateral view **2** head in full-face view **3** body in dorsal view.

##### Worker diagnosis.

Integument shiny black; standing pilosity sparse elsewhere; clypeus with short, rounded lobe; mesonotal suture impressed; petiole nodiform; gaster longer than mesosoma.

##### Note.

Habitat: eucalyptus plantation, grassland, savanna grassland, Uapaca forest, Uapaca woodland, urban/garden. Altitude: 1300–1987 m. Province: Antananarivo. Microhabitat: sifted litter (leaf mold, rotten wood), under stones, on low vegetation, ex soil, ground forager(s), ground nest. Method: MW 20 sample transect, 10 m; SD 20 digging sample transect, 10 m; 20 sardine and ground tuna fish bait; Malaise, 2 traps.

##### Description of minor worker.

Small-sized species. Absolute cephalic size (CS: 0.78±0.12; 0.75–0.81). In full-face view, head ovate, usually somewhat longer than broad, lateral margin of head straight and tapering to front (CWb/CL: 0.34±0.01, 0.34–0.35). Eye subelliptical, protruding, its border smoothly aligned to lateral margins of head (PoOC/CL: 0.09±0.03, 0.08–0.10). Mandibles triangular with six teeth. Anterior clypeal margin not produced, entire, broadly rounded. Antennal scape long, surpassing the occiput by the length of two basal funiculi (SL/CS: 0.35±0.03, 0.34–0.36). In lateral view, mesosoma without anterolateral margination, promesonotum distinctly convex, mesopropodeal suture deeply impressed so that propodeum dorsum is raised to the level of mesopropodeal suture, propodeum angular in profile, with unequal basal and declivitous faces, the former horizontal, the latter straight to evenly concave (MW/ML: 0.18±0.01, 0.17–0.18; MPH/ML: 0.10±0.01, 0.10–0.10). In lateral view, petiole cuneate, its anterior face convex, its posterior face more flattened. Body finely imbricate. Hairs whitish, one pair present on vertex, middle of mesonotum, and propodeum dorsum; two pairs on each side of lateral corner of propodeum; three pairs on lateral margins of petiole. Head and mesosoma reddish, legs light brown, basal face of first gastral segment light brown, and the remaining segment dark brown to black.

##### Distribution and biology.

*Camponotusihazofotsy* is known from the dry forest of Tsimanapetsotsa and Andohaela NP (Fig. 72B). We assume that this species is arboreal because all samples were collected from Malaise traps.

##### Discussion.

*Camponotusihazofotsy* closely resembles *C.tsimelahy*, although morphological examination reveals a number of distinguishing characters. The most interesting character is the form and length of the petiolar scale; in *C.ihazofotsy* the petiole is cuboidal (anterior and posterior face about the same height), while in *C.tsimelahy* the posterior face is higher than the anterior face. In addition, these two species are sympatric at one site, Ihazofotsy. Molecular phylogenetic analysis provides strong evidence that they represent separate lineages (unpublished data).

##### Etymology.

The species epithet refers to the type locality.

##### Additional material examined.

**Province Toliara**: Tsimanampetsotsa National Park, Mitoho Forest, Malaise across trail at escarpment base, –24.0485, 43.75233, 120 m, dense dry forest and transition forest (MG) (CAS).

#### ﻿ Camponotus tsimelahy sp. nov.

Taxon classificationAnimaliaHymenopteraFormicidae

5C119361-6D2C-5AF3-B854-A8A0A4C56EDB

http://zoobank.org/91CA9AF1-550E-41E8-9770-F7F4372E4599

**Figure 69.**
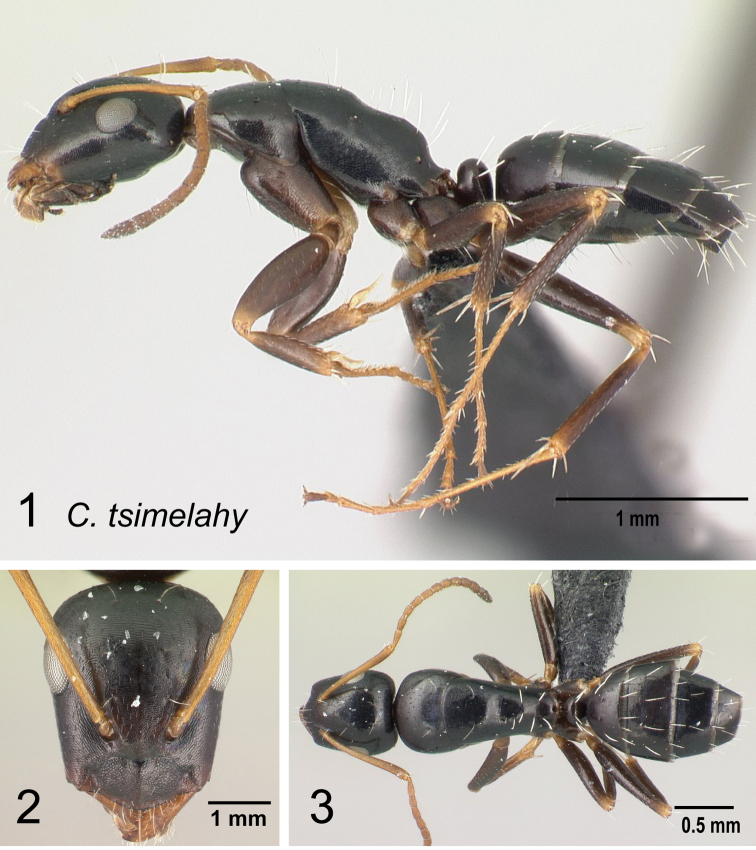
*Camponotustsimelahy* minor worker (CASENT0446651) **1** body in lateral view **2** head in full-face view **3** body in dorsal view.

Figures 43B , 45B , 46A , 69

##### Holotype worker.

Madagascar, Province Toliara, Parc National d ‘Andohahela, Forêt de Manatalinjo, 33.6 km 63° ENE Amboasary, 7.6 km 99° E Hazofotsy, –24.81694, 46.61, 150 m, spiny forest/thicket, 12–16 January 2002 (BLF), collection code: BLF04814, specimen code: CASENT0446651 (CAS). **Paratypes.** One worker with same data as holotype but specimen code CASENT0446549; 3 workers from Parc National d’ Andohahela, Forêt d’ Ambohibory, 1.7 km 61° ENE Tsimelahy, 36.1 km 308° NW Tolagnaro, –24.93, 46.6455, tropical dry forest, 12–16 January 2002 (BLF), collection code: BLF04918, specimen code: CASENT0444910 (CAS).

##### Note.

Habitat: eucalyptus plantation, grassland, savanna grassland, Uapaca forest, Uapaca woodland, urban/garden. Altitude: 1300–1987 m. Province: Antananarivo. Microhabitat: sifted litter (leaf mold, rotten wood), under stones, on low vegetation, ex soil, ground forager(s), ground nest. Method: MW 20 sample transect, 10 m; SD 20 digging sample transect, 10 m; 20 sardine and ground tuna fish baits; Malaise, 2 traps.

##### Description of minor worker.

Medium-sized species. Absolute cephalic size (CS: 0.79±0.04; 0.77–0.81). In full-face view, head ovate, usually somewhat longer than broad, lateral margin of head straight and tapering to front (CWb/CL: 0.29±0.02, 0.28–0.29). Eyes subelliptical, protruding, their borders smoothly aligned to lateral margins of head (PoOC/CL: 0.09±0.00, 0.08–0.09). Mandibles triangular with six teeth. Anterior clypeal margin not produced, entire, broadly rounded. Antennal scape long, surpassing the occiput by the length of two basal funiculi (SL/CS: 0.41±0.03, 0.40–0.42). In lateral view, mesosoma without anterolateral margination, promesonotum distinctly convex, mesopropodeal suture deeply impressed so that propodeal dorsum raised to the level of mesopropodeal suture, propodeum angular in profile, with unequal basal and declivitous faces, the former horizontal, the latter straight to evenly concave (MW/ML: 0.19±0.02, 0.17–0.20; MPH/ML: 0.13±0.01, 0.13–0.14). In lateral view, petiole cuneate with convex anterior face and flat dorsal face. Body finely imbricate. Hairs whitish, one pair present on vertex, middle of mesonotum, and propodeum dorsum; two pairs on each side of lateral corner of propodeum; three pairs on lateral margins of petiole. Head and mesosoma reddish, legs light brown, basal face of first gastral segment light brown and the remaining segment dark brown to black.

##### Distribution and biology.

This new species seems to prefer deciduous forest habitats such as tropical dry forest, gallery forest, spiny forest or thicket, and transitional forest, but is much rarer in humid habitats at lower altitudes (Fig. 72D).

##### Discussion.

*Camponotustsimelahy* may be confused with *C.repens* and *C.ihazofotsy*; all three species share the same sculpturation, form of petiole, and type of suberect setae. However, in profile, the dorsal outline of the mesosoma presents a slightly concave metanotal groove. See the discussion section under *C.ihazofotsy*.

##### Etymology.

The species epithet is in reference to the type locality.

##### Additional material examined.

**Province Toliara**: 18.4 km N Amboasary, –24.87367, 46.39767, 85 m, spiny forest/thicket (BLF) (CAS); Anosy Region, District of Amboasary, Andohaela National Park Parcelle III, Ihazofotsy, 32 km NE Amboasary, –24.83083, 46.53617, 58 m, dry forest, spiny forest (Mike Irwin, Frank Parker, Rin’ha) (CAS); Anosy Region, District of Fort-Dauphin, Andohaela National Park Parcelle II, Tsimelahy, 42 km W of Fort-Dauphin, –24.93683, 46.62667, 176 m, transitional forest (Mike Irwin, Frank Parker, Rin’ha) (CAS); Ifaty 22 km N, –23.18333, 43.61667, 30 m,beach dunes (M.E. Irwin and E.I. Schlinger) (CAS); Ihazofotsy – Parcel III, Andohahela National Park, transitional forest, –24.83483, 46.48683, 80 m, transition between spiny and dry deciduous forests (M.E. Irwin, F.D. Parker, R. Harin’Hala) (CAS); Parc National d’Andohahela, Forêt d’ Ambohibory, 1.7 km 61° ENE Tsimelahy, 36.1 km 308° NW Tolagnaro, –24.93, 46.6455, 300 m, tropical dry forest (FGAT) (CAS); Parc National d’Andohahela, Forêt de Manatalinjo, 33.6 km 63° ENE Amboasary, 7.6 km 99° E Hazofotsy, –24.81694, 46.61, 150 m, spiny forest/thicket (FGAT) (CAS) Tsimelahy – Parcel II, Andohahela National Park, transitional forest, –24.93683, 46.62667, 180 m, transitional forest (M.E. Irwin, F.D. Parker, R. Harin’Hala) (CAS).

**Figure 70.**
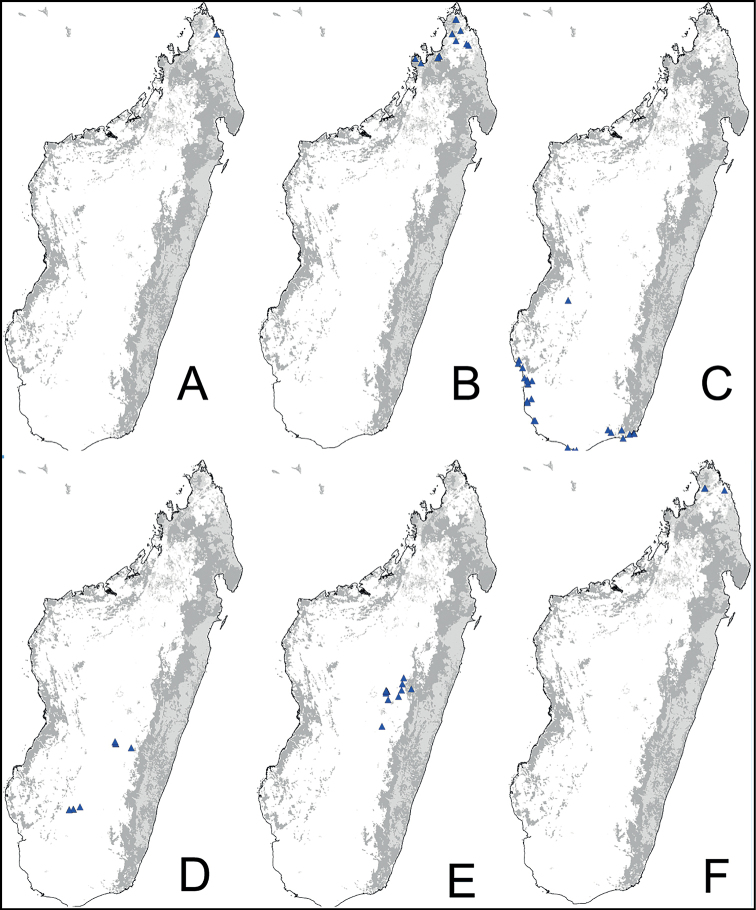
Distribution maps of *C.antsaraingy* species group **A***C.antsaraingy*; *C.efitra* species group **B***C.chrislaini*, *C.ellioti* species group **C***C.ellioti.***D***C.andrianjaka.***E***C.maintilany* and *C.madagascarensis* species group **F***C.ivadia*

### The *Camponotusrobustus* species group

Descriptions of *Camponotusethicus* and *Camponotusrobustus* can be seen in Rakotinirina et al. (2017): *Camponotusethicus* Forel, 1897 on pp. 122–124 and *Camponotusrobustus* Roger, 1863 on pp. 135–137.

#### ﻿ Camponotus zoro sp. nov.

Taxon classificationAnimaliaHymenopteraFormicidae

30EEC29A-33AB-5533-BC30-3620A258F4FA

http://zoobank.org/23F13927-4357-4DBF-9F57-DF1054DFB2A8

Figures 11A, B , 33C, D , 72

##### Holotype worker.

Madagascar, Province Fianarantsoa, Forêt d’ Atsirakambiaty, 7.6 km 285° WNW Itremo, –20.59333, 46.56333, 1550 m, montane rainforest, Malaise trap, 22–26 January 2003 (BLF), collection code: BLF07155, specimen code: CASENT0049853 (CAS). **Paratype.** One worker. Madagascar, Province Antananarivo, 3 km 41° NE Andranomay, 11.5 km 147° SSE Anjozorobe, –18.47333, 47.96, 1300 m, montane rainforest, pitfall trap, 5–13 December 2000 (BLF), collection code: BLF02370, specimen code: CASENT0409150 (CAS).

##### Worker diagnosis.

Integument black, entirely but finely sculptured, appendages dark brown; scape circular; anterior margin of clypeus rounded, not projected; promesonotal suture impressed, dorsolateral margin of propodeal margin.

##### Description of minor worker.

Medium-sized. Absolute cephalic size (CS: 1.02 ± 0.28; 0.92–1.14). In full-face view, head subquadrate (CWb/CL: 0.39±0.01, 0.39–0.40), posterior margin of head broadly convex, lateral margin of head slightly convex. Eyes sub-elliptical, placed laterally, closer to the posterior margin (PoOC/CL: 0.09±0.01; 0.08–0.09). Mandibles triangular with five teeth. Anterior clypeal margin rounded and not produced anteriorly (ClyL/GPD: 0.24±0.01). Antennal scape virtually circular, surpassing posterior cephalic margin by the length of two basal flagella (SL/CS: 0.34±0.04; 0.33–0.36). In lateral view, metanotal groove deeply impressed; pronotum, mesonotum, and propodeum dorsum marginated laterally; mesonotum dorsum inclined posteriorly; propodeum dorsum slightly concave, its lateral carina forming a blunt tubercle with the lateral margin of the declivity (MPD/ML: 0.25±0.02; 0.24–0.25). In lateral view, petiolar node strongly convex anterodorsally and flat posteriorly, node summit convex. Head finely areolate and mesosoma feebly areolate-costulate, gastral tergite finely imbricate. Mandible glabrous. Vertex with three pairs of filiform erect setae; mesonotum and propodeum dorsum with single pair of whitish setae; gastral tergite with short, whitish setae aligned transversally on the middle length of each segment; petiolar node with two or three pairs of whitish setae on its dorsum. Body uniformly dark blackish brown to black, appendages brownish, mandible light brown.

##### Description of major worker.

With characteristics exactly the same as minor worker, but head more trapezoidal (CS: 1.74±0.04, 1.71–1.77).

##### Distribution and biology.

*Camponotuszoro* has been collected by beating low vegetation, sifting leaf litter, and baiting with honey and tuna fish. One worker has been found in Malaise traps from deciduous forest in southern Madagascar (Fig. 72E). In addition, this species has been collected from montane rainforest and Uapaca woodland.

##### Discussion.

*Camponotuszoro* is characterized by a shallow but distinct metanotal groove, and should not be confused with any other species because of the margination of its mesosoma.

**Figure 71.**
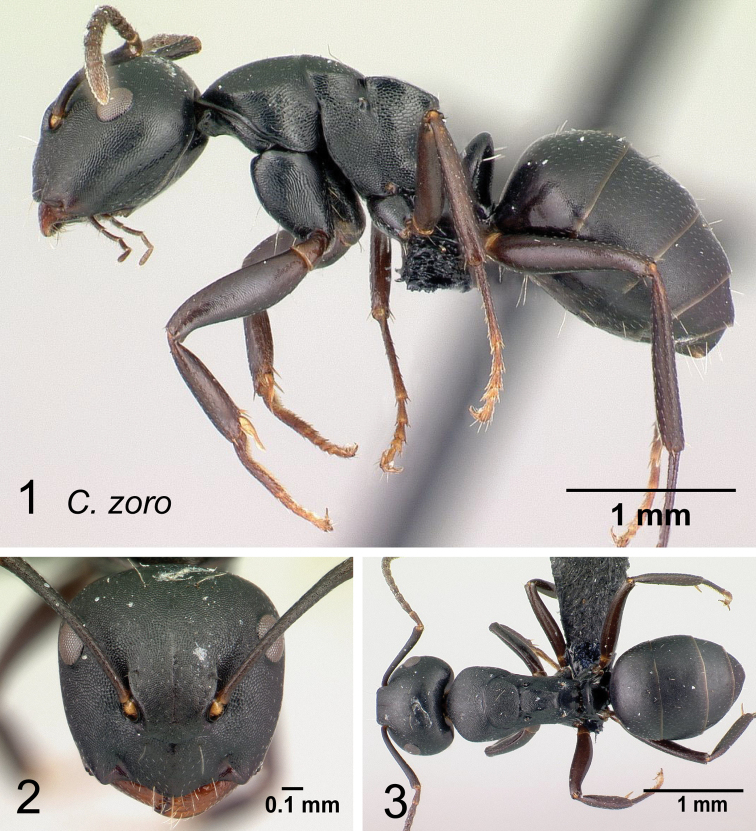
*Camponotuszoro* minor worker (CASENT0049853) **1** body in lateral view **2** head in full-face view **3** body in dorsal view.

##### Etymology.

The new species is named after the structure of the mesosoma; “zoro”means angle.

##### Additional material examined.

**Province Antananarivo**: Navoatra I Non Protected Area, 7.64 km NW Arivonimamo, –18.97806, 47.11929, 1373 m, Uapaca woodland (ARA) (CAS); Ankalalahana, –19.0531, 47.11304, 1314 m, Uapaca woodland (H.E. Marti et al.) (CAS); 3 km 41° NE Andranomay, 11.5 km 147° SSE Anjozorobe, –18.47333, 47.96, 1300 m, montane rainforest (FGAT) (CAS); Reg. Analamanga, St. Forestiѐre Mandraka, –18.9183, 47.91687, 1285 m, montane rainforest (B.L. Fisher, F.A. Esteves et al.) (CAS). **Province Fianarantsoa**: Forêt d’ Atsirakambiaty, 7.6 km 285° WNW Itremo, –20.59333, 46.56333, 1550 m, montane rainforest (FGAT) (CAS). **Province Toamasina**: Réserve Spéciale Ambatovaky, Sandrangato river, –16.7755, 49.26427, 430 m, rainforest (BLF) (CAS).

**Figure 72.**
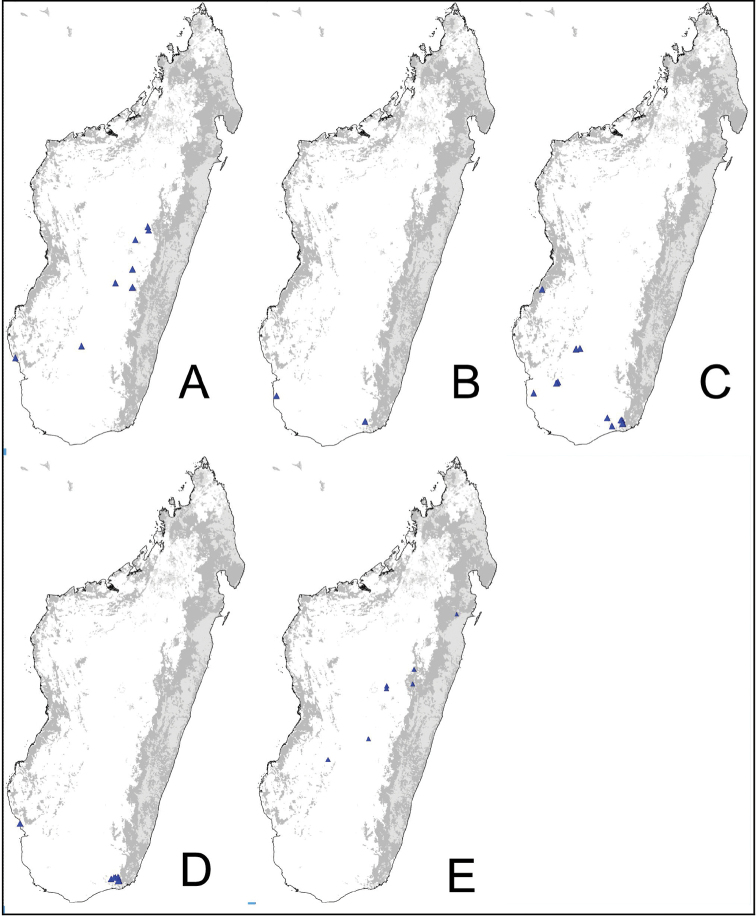
Distribution map of *C.repens* species group **A***C.claveri***B***C.ihazofotsy***C***C.jjacquia***D***C.tsimelahy* and *C.robustus* species group **E***C.zoro*

## ﻿Conclusion

The revision of a large genus such as *Camponotus* requires the integration of multiple lines of taxonomic evidence to delineate species, namely, original morphological descriptions, detailed quantitative morphological measurements, information on ecological distribution, genetic sequence data, and images of type specimens.

## Supplementary Material

XML Treatment for Camponotus antsaraingy

XML Treatment for Camponotus darwinii

XML Treatment for Camponotus norvigi

XML Treatment for Camponotus nossibeensis

XML Treatment for Camponotus radovae

XML Treatment for Camponotus themistocles

XML Treatment for Camponotus ursus

XML Treatment for Camponotus chrislaini

XML Treatment for Camponotus andrianjaka

XML Treatment for Camponotus ellioti

XML Treatment for Camponotus maintilany

XML Treatment for Camponotus ivadia

XML Treatment for Camponotus jjacquia

XML Treatment for Camponotus claveri

XML Treatment for Camponotus ihazofotsy

XML Treatment for Camponotus tsimelahy

XML Treatment for Camponotus zoro

## Supplementary materials

Supplementary material 1 Measurement of individuals specimen Data type COL Explanation note Measurements of minor workers of eleven species of the Malagasy CamponotussubgenusMayria. https://binary.pensoft.net/file/634992This dataset is made available under the Open Daase License (http://opendatacommons.org/licenses/odbl/1.0/). The Open Daase License (ODbL) is a license agreement intended to allow users to freely share, modify, and use this Dataset while maintaining this same freedom for others, provided that the original source and author(s) are credited.Nicole Rasoamanana
